# Biomarkers of moderate alcohol intake and alcoholic beverages: a systematic literature review

**DOI:** 10.1186/s12263-023-00726-1

**Published:** 2023-04-19

**Authors:** Marta Trius-Soler, Giulia Praticò, Gözde Gürdeniz, Mar Garcia-Aloy, Raffaella Canali, Natella Fausta, Elske M. Brouwer-Brolsma, Cristina Andrés-Lacueva, Lars Ove Dragsted

**Affiliations:** 1 Department of Nutrition, Exercise and Sports, Faculty of Science, University of Copenhagen, 1958 Frederiksberg C, Denmark; 2 Polyphenol Research Laboratory, Department of Nutrition, Food Sciences and Gastronomy, XIA School of Pharmacy and Food Sciences, University of Barcelona, 08028 Barcelona, Spain; 3 INSA-UB, Nutrition and Food Safety Research Institute, University of Barcelona, 08921 Santa Coloma de Gramanet, Spain; 4 Centro de Investigación Biomédica en Red de Fisiopatología de La Obesidad Y Nutrición (CIBEROBN), Instituto de Salud Carlos III, 28029 Madrid, Spain; 5 Biomarker & Nutrimetabolomics Laboratory, Department of Nutrition, Food Sciences and Gastronomy, Faculty of Pharmacy and Food Sciences, University of Barcelona, 08028 Barcelona, Spain; 6 Metabolomics Unit, Research and Innovation Centre, Fondazione Edmund Mach, San Michele All’Adige, Italy; 7 Consiglio Per La Ricerca in Agricoltura E L’analisi Dell’economia Agraria (CREA) Research Centre for Food and Nutrition, Rome, Italy; 8 Division of Human Nutrition and Health, Department Agrotechnology and Food Sciences, Wageningen University and Research, P.O. Box 17, 6700 AA Wageningen, The Netherlands; 9 Centro de Investigación Biomédica en Red de Fragilidad Y Envejecimiento Saludable (CIBERFES), Instituto de Salud Carlos III, 28029 Madrid, Spain

**Keywords:** Biomarkers of food intake, Alcohol, Ethanol, Alcoholic beverages

## Abstract

The predominant source of alcohol in the diet is alcoholic beverages, including beer, wine, spirits and liquors, sweet wine, and ciders. Self-reported alcohol intakes are likely to be influenced by measurement error, thus affecting the accuracy and precision of currently established epidemiological associations between alcohol itself, alcoholic beverage consumption, and health or disease. Therefore, a more objective assessment of alcohol intake would be very valuable, which may be established through biomarkers of food intake (BFIs). Several direct and indirect alcohol intake biomarkers have been proposed in forensic and clinical contexts to assess recent or longer-term intakes. Protocols for performing systematic reviews in this field, as well as for assessing the validity of candidate BFIs, have been developed within the Food Biomarker Alliance (FoodBAll) project. The aim of this systematic review is to list and validate biomarkers of ethanol intake per se excluding markers of abuse, but including biomarkers related to common categories of alcoholic beverages. Validation of the proposed candidate biomarker(s) for alcohol itself and for each alcoholic beverage was done according to the published guideline for biomarker reviews. In conclusion, common biomarkers of alcohol intake, e.g., as ethyl glucuronide, ethyl sulfate, fatty acid ethyl esters, and phosphatidyl ethanol, show considerable inter-individual response, especially at low to moderate intakes, and need further development and improved validation, while BFIs for beer and wine are highly promising and may help in more accurate intake assessments for these specific beverages.

**Supplementary Information:**

The online version contains supplementary material available at 10.1186/s12263-023-00726-1.

## Introduction

Ethanol (“alcohol”) intake (“drinking”) has been associated with numerous adverse effects on health and on quality of life, whereas light to moderate drinking, typically 1–2 drinks/day in Western countries, has been associated with beneficial health effects [1, 2]. In most countries, alcohol intake is not recommended, whereas upper limits for moderate alcohol intake have been set at 1 or 2 units a day. The amount of alcohol in a “unit” or a standard “drink” varies from around 8–14 g (10–17.7 mL) between different countries, the lowest currently in the United Kingdom (UK) and the highest in the United States of America (USA) [3, 4]. Assessing alcohol intake is important for health and societal research, but also for forensic and other legal causes to investigate abuse/misuse of alcohol or to monitor abstinence when drinking is prohibited [5–7]. Numerous tools have therefore been developed in order to assess alcohol intake, including questionnaires, physiological measures, and biochemical assays on samples such as blood, urine, or hair [8, 9]. However, the subjective tools (i.e., questionnaires) to assess alcohol intake are known to be biased by social and personal attitudes to drinking [10] and objective measures have therefore been a subject of considerable technical interest [11]. These objective measures may largely be divided into (a) direct measures relating to alcohol metabolites and (b) indirect measures relating more to the physiological and biochemical effects of drinking. Indirect markers are dominating research on risks and abuse of alcohol intake (i.e., longer-term intakes), while direct markers are used most often to measure recent intake.

For the purpose of nutritional assessment, there are interests in biomarkers of both recent and longer-term alcohol intake to study the associated risks and potential benefits [12]. Moreover, there is a considerable interest to discriminate between the different alcoholic beverages: that is, to objectively assess the type of alcoholic beverage consumed. For instance, physiological or health effects specifically related to red wine or beer have recently been reviewed [13–15]. Assessing compliance is also important and demands objective tools to assess alcohol consumption; factors such as the time lapse since the last drink, the frequency of drinking, and the different beverages consumed are also important questions in need of objective biomarker strategies.

The predominant source of alcohol in the diet is alcoholic beverages, including commonly consumed products such as beer, wine, spirits and liquors, sweet wine, ciders, and various niche products, e.g., kombucha. Besides, alcohol is also formed in several food fermentation processes and may exist as residuals in some foods [16] or may even be inhaled from environmental sources or formed to a variable extent in the human body [17]. While oral intake constitutes quantitatively close to 100% of relevant exposures in nutrition, some examples of other routes exist and have been of importance in forensic cases [18]. For the purpose of nutritional intake biomarkers of alcoholic beverages, the source, timing, frequency, and amount are all among the relevant variables to consider when assessing biomarker quality and use [19]. The aims of the current systematic review are (a) to list all putative markers suitable for the measurement of moderate alcohol intakes and (b) to validate these markers according to common guidelines, thereby pointing out what evidence is still missing in the scientific literature. In the following sections, we report a systematic assessment of the literature on the biomarkers of ethanol intake per se and of biomarkers related to most of the categories of alcoholic beverages, which contribute most to the overall alcohol production. The review explicitly excludes biomarkers related only to intakes above moderation but has an additional focus on inter-individual response variability as well as any natural background levels of the biomarkers in subjects with no intake. What constitutes moderate intake is historically and geographically diverse, and we have therefore covered the studies on biomarkers within the ranges reported as common social drinking, thereby excluding chronic abuse. Narrative reviews on alcohol intake biomarkers in relation to forensic and clinical studies have been published recently [15, 18].

## Methods

### Selection of food groups

For the present review, five subgroups of alcoholic beverages including the most widely consumed (beer, cider, wine, sweet wine, and spirits/distillates) were selected. Biomarkers were also assessed for general alcohol/ethanol consumption. A systematic literature search was carried out separately for each alcoholic beverage subgroup and for alcohol/ethanol as detailed below.

### Primary literature search

The reviewing process was performed following the Guidelines for Food Intake Biomarker Reviews (BFIRev) previously proposed by the FoodBAll consortium [20]. Briefly, a primary research was carried out in three databases (PubMed, Scopus, and the ISI Web of Science) using a combination of common search terms: (biomarker* OR marker* OR metabolite* OR biokinetics OR biotransformation) AND (trial OR experiment OR study OR intervention) AND (human* OR men OR women OR patient* OR volunteer* OR participant*) AND (urine OR plasma OR serum OR blood OR hair OR excretion) AND (intake OR meal OR diet OR ingestion OR consumption OR drink* OR administration) along with the specific keywords for each alcoholic beverage subgroup (Supplementary Table S1). The fields used as a default for each of the databases were as follows: all fields for PubMed, article title/abstract/keywords for Scopus, and topic for ISI Web of Science. Breath alcohol was not systematically covered in the primary search, but papers including data on breath ethanol levels were kept.

**Fig. 1:**
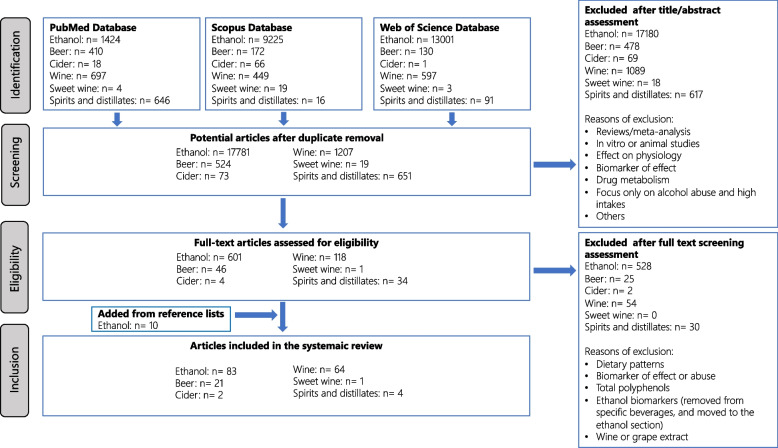
Flow chart of the study selection according to guidelines for biomarker of food intake reviews (BFIRev) procedure

The last search was carried out in March 2022. It was limited to papers in the English language, while no restriction was applied for the publication dates. The research papers identifying or using potential biomarkers of intake for each alcoholic beverage subgroup and for total alcohol consumption were selected according to the process outlined in Fig. 1. Articles showing the use of the markers in human observational or intervention studies were considered eligible. Additional papers were identified from the reference lists of these papers and from reviews or book chapters identified through the literature search. The exclusion criteria for the primary search were articles focused on the following effects of alcoholic beverage subgroups or ethanol/alcohol intake, while not using a biomarker of intake: (1) cholesterol, plasma lipids, inflammatory biomarkers, or blood pressure; (2) cardiovascular diseases, diabetes, or gout; (3) high alcohol consumption in relation to alcoholism; (4) other biomarkers (e.g., contaminants and effect markers), or (5) animal, in vivo and in vitro studies. Papers considering biomarkers of relevance only to alcohol abuse were omitted, except if they provided important information on, e.g., kinetics.

### Secondary literature search

For each identified potential biomarker of food intake (BFI), a second search step was performed to evaluate its specificity using the same databases (PubMed, Scopus, and the ISI Web of Science). The search was conducted with (“the name and synonyms of the compound” OR “the name and synonyms of any parent compound”) AND (biomarker* OR marker* OR metabolite* OR biokinetics OR biotransformation OR pharmacokinetics) in order to identify other potential foods containing the biomarker or its precursor. Specific as well as non-specific biomarkers were selected for discussion in the text, while only the most plausible candidate BFIs have been tabulated, including the information related to the study designs and the analytical methods.

### Marker validation

To evaluate the current status of the validation of candidate BFIs and to suggest the additional steps that are needed to reach the full validation, a set of validation criteria [19] was applied for each candidate BFI. The assessment was performed by answering 8 questions related to the analytical and biological aspects of the validation together with a comment indicating the conditions under which the BFI is valid (see explanation under Table 1). The questions were answered with Y (yes, if questions were fulfilled under any study conditions), N (no, if questions had been investigated but they were not fulfilled under any conditions), or U (unknown, if questions had not been investigated or answers were contradictory) according to the current literature.

**Table 1:** Overview of the current level of validation^a^ of candidate BFIs

Food item	Metabolites	Biofluid locations	Questions^b^							
			**1**	**2**	**3**	**4**	**5**	**6**	**7**	**8**
Alcohol	Ethanol	Breath/blood/urine	Y	Y	Y	Y^c^	Y	Y	Y	Y
	Methanol	Blood	Y	Y^d^	Y	N	N^d^	Y	Y	U
	Acetaldehyde	Blood/urine	Y	U	U	U^c^	U	U	U	U
	Ethyl glucuronide	Blood/urine	Y	Y^d^	Y	Y^c^	Y	Y	Y	Y
		Hair	Y	Y^d^	U	N^c^	Y	Y	Y	U
	Ethyl sulfate	Blood/urine	Y	Y^d^	Y	Y^c^	Y	Y	Y	Y
		Hair	Y	U	U	U	U	U	U	U
	Fatty acid ethyl esters	Blood	Y	Y^d^	Y	Y^c^	Y	N	Y	Y
		Hair	Y	Y^d^	N	N	Y	U	U	U
	Phosphatidylethanols	Blood/erythrocytes	Y	Y^d^	Y	Y	Y	Y	U	N
Beer	Iso-α-acids (IAAs)	Blood/urine	Y	Y	Y	Y	Y	U	Y	Y
	IAAs + reduced IAAs	Blood/urine	Y	U	Y	Y	Y	U	Y	U
	Isoxanthohumol	Urine	Y	Y/N^e^	Y	Y	Y	Y	Y	Y
	Hordenine and its metabolites	Blood/urine	Y	U	Y	U	Y	U	Y	Y
	Combined marker^f^	Urine	Y	U	Y	Y	Y	U	U	U
	Humulinone	Urine	Y	U	U	U	U	U	U	U
Wine	Resveratrol and conjugated metabolites	Blood/urine/LDL	Y	U	Y	Y	Y	Y	Y	Y
	Tartaric acid	Urine	Y^g^	Y	Y	Y	Y	Y	Y	Y
Aniseed spirit	Anethole	Blood	Y	Y	Y	Y	Y	U	Y	U
Peppermint liquor	Menthone	Blood	Y	Y	Y	U	N	U	Y	U
	Isomenthone	Blood	Y	Y	Y	U	N	U	Y	U
	Neomenthol	Blood	Y	Y	Y	U	N	U	Y	U
	Menthol	Blood	Y	Y	Y	U	N	U	Y	U

^a^The answers Y and N in this table mean that in specific situations, the marker has shown validity for the aspect in question. For any specific use, the marker validity has to be reconsidered carefully

^b^(1) Plausibility, (2) dose–response, (3) time-response, (4) robustness, (5) reliability, (6) stability, (7) analytical performance, (8) reproducibility

^c^Unexplained background levels commonly reported

^d^Not well documented at intakes below 5–10 g alcohol

^e^Y is for males, N for females

^f^*N*-methyl tyramine sulfate, iso-α-acids, tricyclohumols, pyro-glutamyl proline, 2-ethyl malate

^g^Not plausible as a unique marker of wine intake but as a general marker of grape products

## Results

### Alcohol/ethanol intake

**Fig. 2:**
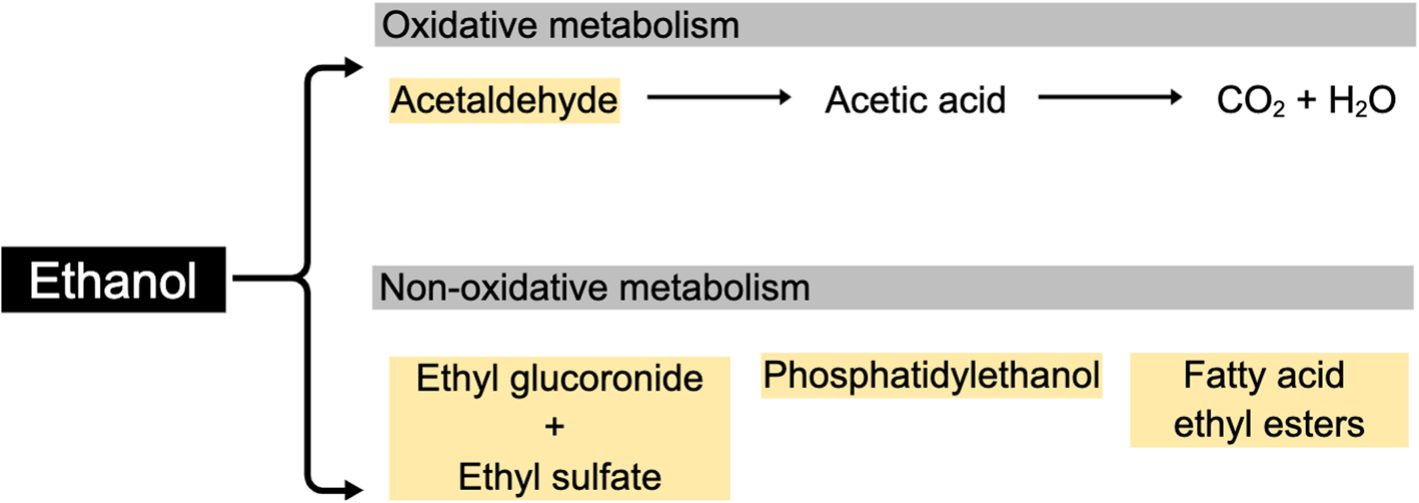
The metabolism excretion of ethanol in the human body

**Fig. 3:**
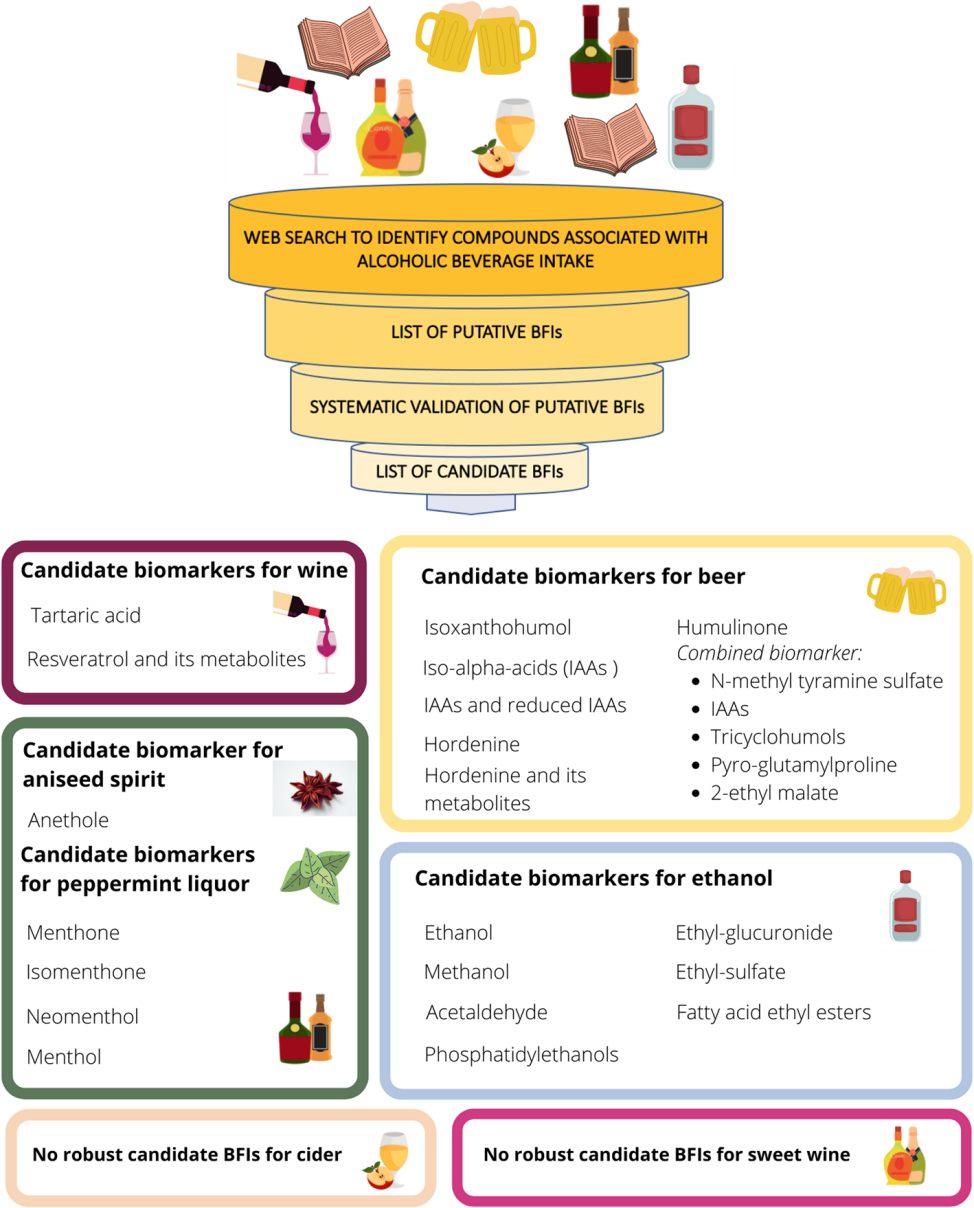
Summary of the candidate biomarkers for alcohol and specific alcoholic beverages

The search for references to alcohol intake biomarkers resulted in 20,255 potentially relevant papers covering intakes of ethanol, beer, wines, spirits, and liqueurs; however, most of these were not related to biomarker development or validation but to many other fields within alcohol research, especially alcoholism (*n* = 19,451), see Fig. 1. In Table 1 there is a list of the candidate biomarkers identified for alcohol intake representing all the identified studies, along with data for their validation as biomarkers at low to moderate alcohol intakes. Table 1 builds upon the identified studies listed in Supplementary Table S2. The samples used include blood, urine, breath, and hair. The direct alcohol intake biomarkers in these various samples are almost all metabolites of alcohol, i.e., ethanol itself, acetaldehyde, or their adducts with other biomolecules (Fig. 2). For some beverages, especially beer and wine, some characteristic components were observed as biomarkers. The proposed candidate biomarkers reflecting alcohol and specific alcoholic beverage intake are shown in Fig. 3.

#### Ethanol and methanol

Ethanol per se can be measured in the breath, blood, serum, and plasma as well as in the hair and urine, and all of these samples are commonly used to assess recent exposure in forensics. The most common marker used to assess recent alcohol intake is ethanol vapor in exhaled air, which is used routinely to test vehicle drivers, pilots, and other machine operators. The concentration of ethanol in the blood, urine, hair, or tissue is used to assess recent exposure in forensics. Within 2–4 h of moderate alcohol intake (1–2 drinks) and around 12 h after high, acute alcohol intake (binge drinking), ethanol itself cannot be measured any longer in the breath, blood, or freshly voided urine [21]. The presence of ethanol in human samples depends to a large extent on the exposure, the time since ingestion, and the genetics and lifestyle of the individual. Ethanol is metabolized by alcohol dehydrogenase (ADH, EC 1.1.1.1) to acetaldehyde and gene variants with very fast clearance result in fast removal, but these variants are rare in subjects of European or African descent but more common in the Middle East and Asia [22]. Most human subjects have zero-order clearance of ethanol from the blood, meaning that the rate of metabolism is independent of the ethanol concentration with clearance at around 0.15 g/L/h after 2 or more drinks, due to saturation of metabolism. Depending on body size and composition, this means burning of around one unit of alcohol (10–15 g, depending on definition) in 1.25 (men) to 1.75 (women) hours, but women may have higher elimination rates than men, partially compensating for the difference in distribution volume [23]. At lower intakes when the major degradation pathway is no longer saturated, the rate gradually approaches first-order kinetics, meaning that elimination becomes slower. High levels of ethanol inhibit the activity of ADH towards other alcohols, thereby causing the accumulation of methanol and propanol. Ethanol is found at low levels in many foods, especially fermented foods, and high endogenous production by fermentation (auto-brewing) is also known in rare cases in children as well as adults [24]. Low steady-state levels in subjects below 0.1 mg/dL have been reported by sensitive analyses (summarized in [25]).

Methanol is slowly formed during several endogenous metabolic processes, and low levels are also coming from foods; the ethanol concentrations necessary for methanol accumulation may be observed already after a few hours of drinking. Therefore, measuring methanol in the blood or urine is a useful marker within a day of alcohol intake to reveal a recent (binge) drinking episode or alcohol dependence (> 5 mg/L/day) [18]. It has recently been shown that methanol and 1-propanol are formed from ethanol in humans after acute intake of 40–90 g ethanol and both compounds may therefore serve as potential markers of binge drinking [26]. The half-life of 1-propanol, which is also a potential microbial metabolite [27], is similar to that of ethanol, while methanol has a longer half-life making it useful for examining high drinking episodes within 1–2 days. However, moderate alcohol intakes may not inhibit ADH sufficiently to increase methanol levels, and none of the alcohol congeners is therefore useful biomarkers of social (moderate) drinking.

The distribution volume for ethanol is mainly the water phase, meaning that subjects with a similar body weight will differ in blood ethanol concentration after exposure, depending on their fat mass. Thus, ethanol in the blood, plasma, and serum is a useful biomarker that will in most cases reflect recent intake in a dose-related manner. The concentration in the breath is directly proportional to the concentration in the blood at moderate intakes, so it will also reflect both dose and distribution volume. However, the breath test has limitations and must be confirmed by other biomarkers, especially in heavy drinkers [28, 29].

#### Acetaldehyde

The primary metabolic product of ethanol is acetaldehyde formed by ADH [30], which may also be directly quantified in blood and urine samples. However, due to its reactivity with amino groups in proteins, acetaldehyde is reversibly or irreversibly bound to proteins. Acetaldehyde is further metabolized to acetate by aldehyde dehydrogenase (ALDH, EC 1.2.1.3), which is also polymorphic. In a recent study, acetaldehyde in the whole blood was measured in wild-type homozygous and ALDH-heterozygous Koreans by dinitrophenylhydrazine derivatization and liquid chromatography-mass spectrometry (LC–MS/MS) after a single challenge (0.8 g/kg body weight) with approximately 4 units of vodka [31]. No background was observed before the challenge, and blood levels were low in wild-type homozygous volunteers, but peaked at 15 times higher levels in the heterozygotes ½–1 h after the drink, and were still detectable at 6 h. Further validation of the method was not reported. Blood alcohol concentration (BAC) was higher in the heterozygotes, indicating that there may be feedback inhibition of ADH by acetaldehyde [31]. In a recent paper on the carbonyl metabolome, no acetaldehyde was reported in the urine after derivatization with danzyl hydrazine [32]. No information was provided on the human donor or the collection of the urine sample analyzed in this methods paper.

Protein adducts of acetaldehyde have been used to assess the average alcohol intake over the lifetime of the protein or cellular structure used for the assessment. For instance, acetaldehyde adducts in erythrocytes could theoretically be used to estimate intakes over its lifetime of around 120 days, while acetaldehyde in each centimeter of hair, starting from the scalp, might become a future method to measure average exposures per month [33].

Acetaldehyde binding to amino groups in proteins results in the formation of Schiff bases. As long as these bases are not reduced, acetaldehyde can be released, and this is accelerated by acid and heat; this procedure was used already in 1987 to design a highly sensitive assay using plasma proteins or hemoglobin, and the method was later validated and widely used by insurance companies in the USA to identify subjects at high risk of being alcohol abusers [34, 35]. The method has a relatively high background in teetotalers for both plasma protein and hemoglobin adducts of acetaldehyde, overlapping with levels observed in alcoholics [34]. This would indicate that background metabolic processes leading to acetaldehyde formation are quite common and active. These methods have so far not been used to report levels in low or moderate alcohol users. Other methods to determine acetaldehyde have been developed using capillary electrophoresis (CE) or gas chromatography (GC) coupled with MS to identify acetaldehyde-protein adducts [36, 37]. In the CE-based study, an investigation of levels in three moderate drinkers (< 2 units/day) and one non-drinker were compared, showing apparent acetaldehyde-hemoglobin peaks only in the three drinkers [36]. In the GC–MS-based study, 20 human samples were also analyzed, and in this case, no overlap between the levels in 10 non-drinkers and 10 alcoholics was observed. However, background levels in non-drinkers were quite high and variable. The levels observed in this small sample set were apparently independent of age, smoking, ADH and ALDH genotypes, or body mass index [37]. Larger studies are needed to confirm this and to address other aspects of method validation (Table 1). Additional methods have been proposed, e.g., the formation of a cysteinyl-glycine adduct measurable in rat urine has been reported [38]. A new method for measuring free cysteine- and cysteinyl-glycine adducts of acetaldehyde in urine and plasma has recently been published, but adducts were not found in humans after acetaldehyde exposure due to too high background levels [39]. However, these adducts are not stable over time in serum and were found to be destabilized in the presence of strong nucleophiles [40].

Acetaldehyde is genotoxic and reacts directly with DNA bases, to form, e.g., N^2^-ethyl-deoxyguanosine residues and several other DNA adducts [41, 42]. These may be measured directly in tissue DNA, or they may be repaired, forming excretion products to be measured in urine. The adducts measured in DNA have been used as markers of alcohol dose in investigations on ethanol intake and show dose dependence and time course of repair and elimination in oral cavity exfoliated epithelium. Single moderate alcohol doses lead to measurable acetaldehyde in the saliva and in exfoliated oral cells [43]. The oral cavity adducts may therefore be candidate biomarkers of recent alcohol intake, especially for liqueurs providing high local concentrations. However, the effect was only observed locally; acetaldehyde adduct formation in lymphocytes and granulocytes was not affected by three single moderate doses provided in the same pilot study [41]. In conclusion, acetaldehyde forms adducts with proteins and DNA, and moderate exposures may lead to increases; however, relatively high background levels are often observed potentially limiting usefulness and thorough validation will be needed for these methods to translate into useful biomarkers of moderate alcohol intake.

#### Ethyl glucuronide

Ethanol is conjugated by UDP-glucuronosyl transferases (UDPGT; EC 2.4.1.17) to a low extent by phase II metabolism into ethyl glucuronide (EtG). EtG was first observed and later isolated from the urine of ethanol-exposed rabbits [44, 45]. The first quantification in human urine was not performed until 1995 [46], and soon after, it was suggested as a biomarker of alcohol intake in forensics [47]. For about 20 years now, EtG has become widely used in forensic studies due to its sensitivity and reliability. However, most studies are related to abuse and therefore beyond the scope of this review. Ever since the earliest findings in animal studies, it is clear that several UDPGT isozymes in rabbits and in rodents [48] can conjugate ethanol. The Km for the most active human UDPGT isozymes is on the order of 8 mM [49]. This corresponds to the peak blood alcohol concentration after intake of around 10 g alcohol, and the rate of formation of EtG is therefore expected to be lower at low intakes and to increase at higher intakes. This has been confirmed in several studies in humans, where non-linear dose-concentration and dose-excretion curves for EtG have been observed showing increased fractional levels with the administered dose [25, 50, 51]. Measurements of EtG during pregnancy to reveal sporadic social drinking have also been investigated in forensics showing variable frequencies of positive samples in different populations, with many cases among women reporting total abstinence [52, 53]. Characteristic individual EtG background levels in urine have been observed in alcoholics housed for weeks in a closed ward by repeated daily sampling during abstinence [25]. These results indicate that low levels of EtG may occur even without alcohol intake, but long-term fully controlled studies to confirm this are needed.

EtG is quite water soluble and is therefore often assessed by GC–MS or LC–MS/MS in blood or urine samples collected within hours of exposure. The elimination kinetics are slower than for ethanol itself, and the ability to measure recent alcohol exposure by this marker may therefore extend beyond 12 h in the blood [54] and 24 h or more in the urine, depending on the dose and the sensitivity of the analysis [46]. The time windows for measuring blood EtG and excretion of EtG in urine are important for assessing recent intakes based on spot samples. In several studies, serial blood samples have been collected to compare EtG with BAC or controlled alcohol intakes (*n* = 1–54) [55–57]. The useful time window for EtG measurement after a single ethanol dose was reported to be at least 10 h in the blood and 24 h in the urine after a peak BAC of 0.12 g/L (*n* = 10) [55]. In another study showing dose–response, the apparent time window for EtG in the serum was 25–50 h, depending on alcohol dose, with the lowest dose tested being ~ 25 g (2–2½ units) [57]; no background level above the method cutoff for EtG could be measured after 1 week of abstaining. In a recent study, there was a high variability in the peak level and total EtG excretion in 24 volunteers after drinking 48 g of alcohol as beer [58]. Inter-individual variation in peak serum levels of EtG (at 10–20 h) and time to reach plasma levels below LOQ (range: 35–100 h) has been reported after binge-drinking of 64–172 g alcohol within 6 h [59, 60]. Since EtG in urine depends on the diuresis, it is often recommended to correct EtG for creatinine excretion; this method improves analyses of excretion kinetics [56]. The limit of quantification (LOQ) for EtG has been reported to be as low as 0.02 mg/L [61], well below the widely accepted cutoff at 0.1 mg/L, which corresponds to a level typically observed in a spot urine sample collected around 24 h after intake of 10 g of alcohol. A few documented cases exist of measurable EtG in urine above this level from non-drinkers, including pregnant women and children, indicating that sources of alcohol or EtG exposure are likely to exist in non-drinkers; these sources may include the use of hand sanitizers, gut microbial fermentation, and possibly consumption of fermented foods [25, 53]. EtG is stable in the autoanalyzer at 4 °C for up to 96 h [62]. In a study of EtG-free blood samples spiked with ethanol, EtG formation was observed at 37 °C after 3 days; degradation of the EtG in positive blood samples was observed during storage at 25 °C for > 3 days or at 37 °C for > 1 day, but EtG was stable at 4 °C or − 20 °C [63]. Measurement of EtG with a dipstick has been shown to be insufficiently sensitive for routine use [54], but in a prospective cohort study among subjects with mild symptoms of kidney disease values measured by dip-sticks correlated well with self-reported alcohol intake (*r* = 0.68, *p-*value < 0.001) [64]. However, a large part of the subjects reporting no intake (~ 50%) exhibited EtG values above the 0.1 mg/L cutoff, suggesting potential effects of kidney disease or its etiological factors on EtG formation or excretion.

EtG also accumulates in the hair, making hair samples an attractive means of potentially assessing past exposures [65, 66]; the method seems specific for heavy drinking, but sensitivity issues and possibly also inter-individual variation may render it less useful for the determination of intake levels within light to moderate drinking [67, 68]. Improved methods for extraction and milling of the hair samples increase the sensitivity [69], but only few studies have experimentally investigated relevant hair EtG levels at different levels of social drinking. In a study of 15 students, excessive drinkers were clearly identified while there was an overlap between levels observed in students reporting moderate intakes or abstinence and only one of five abstainers had levels below detection [62]. In a study of a few teetotalers (children) and social drinkers (up to 20 g/day), all samples were negative (< LOD of 2 pg/mg hair) [70]. At intakes of 0, 1, or 2 drinks/day for 12 weeks, both dose–response and time response were observed at the group level using standardized protocols for hair analysis [71]; these protocols have been debated and could possibly be improved [72, 73]. Standardized cutoffs for very low or no drinking and for heavy drinking have been agreed upon at 7 and 30 pg/mg hair, respectively [74]. Background levels are still occasionally found in abstainers [75], and levels tend in general to be higher with high body mass index or in subjects with kidney damage [75, 76]. Hair EtG measurements may also be less sensitive at low alcohol intakes (≤ one drink per day) [77]

In conclusion, EtG measured by LC–MS/MS in the blood or urine are short-term markers of alcohol intake with a time window exceeding that of BAC, with well-known time- and dose–response, and with legal cutoff levels for background exposures that are rarely exceeded in non-drinkers. However, levels between the suggested cutoffs of 0.1 and 0.5 mg/L have been observed repeatedly in non-drinkers, and intakes below 10 g alcohol may occasionally overlap with background levels in a time window of 24 h. In hair, EtG by LC–MS/MS is a well-validated marker for high alcohol consumption; however, it is highly variable and less sensitive in subjects with lower intakes.

#### Ethyl sulfate

Ethyl sulfate (EtS) is another common, low-abundance phase II metabolite of ethanol with characteristics very similar to EtG. The first data on its formation also came more than 70 years ago from animal studies (i.e., rats) [78] and the first human urine identification and first legal method were published during 2004 [6, 79]. Already some of the earliest studies confirm EtS as a plausible marker since several aliphatic alcohols were substrates of mammalian sulfotransferases (EC 2.8.2.2) [78]. Several human isoenzymes can perform the sulfation of ethanol in vitro with quite variable conjugation rates as already shown in 2004, but in 10 volunteers provided with 0.1 or 0.5 g ethanol per kg body weight (3–27 g), the excreted amount varied only by a factor of 3 within as well as between subjects, independent of sex [80]. Variability in human absorption and excretion kinetic constants in 13 male volunteers after a dose of 30–60 g ethanol was also reported to be only around 2 for each [81]. The time-response in 13 volunteers was also investigated after consumption of a low alcohol dose (0.1 g/kg body weight) showing a peak at 2–5 h and a time window of detection of 6–10 h; preliminary indication was also shown of a higher fractional as well as total excretion at a 5 times higher dose (time window ≥ 24 h) [80]. In a recent study, in 24 male and female volunteers provided with 47.5 g alcohol (beer) within 15 min, the inter-individual variability in EtS excreted over 10.5 h was more than 100-fold at the excretion peak apex and with a variable peak time of 2.5–8.5 h [58]. EtS showed considerable correlation with measured levels of EtG before as well as after the drink. In analogy with EtG, background levels of EtS are only observed by more recent, sensitive analyses [57]. Background levels in most volunteers after 3 days of abstaining were high (> 1 mg/L for EtS and 1.8 mg/L for EtG) with a reasonable correlation between markers (*r*^2^ = 0.56). In this study, one of the volunteers hardly produced any EtS or EtG after drinking 47 g of alcohol in 15 min, while a few others only showed very low levels, indicating that these markers may miss a small percentage of drinkers. ADH genotyping was not provided, but the authors suggest polymorphic phase 2 enzymes to be the main cause of this variability [58]. However, this is less likely considering the high correlation between the EtS and EtG markers. BAC at 30 min after the drink was apparently not associated with low EtG or EtS excretion, and further investigation to identify the causes of such marker variability is needed in order to use EtS (and EtG) in routine analysis at low intakes. The higher fractional excretion of EtS at higher doses indicates a relatively high Km in analogy with EtG [80]. A 25-fold higher Km was reported for the formation of human EtS than for EtG in vitro [49] but this does not seem to correspond with the observed EtS and EtG formation in humans showing similar dose- and time-response compared with EtG [57]. Additional study of Km for the human sulfotransferases forming EtS is therefore needed. In a study of human blood samples that were blank, alcohol-spiked, or positive for EtS no formation or degradation of EtS was observed over 7 days in any samples at temperatures from − 20 to 37 °C [63]. EtS is also stable in a standardized anaerobic bacterial incubation while < 20% were lost under aerobic conditions over 28 days at 20 °C in the dark [82].

Only a single publication has so far evaluated EtS in hair as a marker of alcohol intake, and it was reported that it may actually compare favorably with hair EtG; however, more studies are needed before it can be validated as a biomarker of low or moderate alcohol intakes [83].

In conclusion, EtS in the serum or urine is a well-validated biomarker of recent alcohol intake, comparable with EtG. Likewise, EtS measurements are accurate and precise and show dose- and time-response even at quite low intakes, but some subjects produce very little while others have measurable background levels after abstinence. Care must therefore be exercised in the interpretation of individual levels in the lower range. Hair EtS has not been extensively validated and needs further investigation.

#### Phosphatidylethanols

Phosphatidylethanols (PEths) are polar fatty acid esters, known to be formed enzymatically by phospholipase D in red blood cells, especially at high blood alcohol levels [84]. In vitro studies also indicate that relatively high blood ethanol concentrations are needed for PEth formation, with PEth 16:0/18:1 as the most abundant species [85]. PEth has therefore been historically regarded as a useful marker of high alcohol intake, e.g., in forensics [86]. However, the levels observed at lower intakes have not been well studied until recently; studies on alcoholics have indicated variable levels even at intakes below 40 g/day during less intense drinking periods, overlapping with levels observed at much higher intakes [86]. PEth levels in dried blood spots were shown not to differ from those in fresh blood samples in a group of 40 alcohol detoxification patients attending a ward; all patients had levels indicating problem drinking, but the levels varied approximately 100-fold [87].

Some studies have investigated the PEth blood levels over time in abstainers, after withdrawal from heavy intakes, or during experimentally controlled multiple or single moderate alcohol doses or abstaining [88–90]. One study investigated PEth over time during abstention [89]; in this study of 56 alcoholic withdrawal patients and 35 non-drinking in-patients, PEth was measured after 4 weeks without alcohol intake. The non-drinkers had blood PEth < 0.3 µM (LOQ for detection by an older light-scattering technique) throughout, and the two groups were easily differentiated with 100% specificity (the area under the receiver operating characteristics curve (AUROC) = 0.97) using a cutoff at 0.36 µM. Some withdrawal patients had levels below the cutoff despite measurable BAC at admission. This study demonstrates that abstainers and heavy abusers can mostly be discriminated by PEth after 1–4 weeks [89] but that inter-individual differences in formation and response levels exist and may complicate judgment in individual cases [84, 85]. Another study that included 36 subjects (32–83 years old) evaluated the change in PEth levels at 3–4 weeks intervals in subjects attending outpatient treatment to reduce drinking. Comparison of individual changes in PEth concentration vs. past 2-week alcohol consumption between two successive tests revealed that an increased ethanol intake by ∼ 20 g/day (1–2 drinks) elevated the PEth concentration by on average ∼ 0.10 μM, and vice versa for decreased drinking [91]. The elimination characteristics of three PEth homologs have been studied in 47 heavy drinkers during approximately 2 weeks of alcohol detoxification at the hospital. During abstinence, the elimination half-life values ranged between 3.5–9.8 days for total PEth, 3.7–10.4 days for PEth 16:0/18:1, 2.7–8.5 days for PEth 16:0/18:2, and 2.3–8.4 days for PEth 16:0/20:4. Individual significant difference in the elimination rates between different PEth forms was also found, indicating that the sum may be the best biomarker [92].

In a randomized parallel intervention study, PEth during abstention or moderate alcohol intakes (16 g/day for women and 32 g/day for men) were compared in 44 volunteers over a period of 3 months [88]. In the abstaining group, PEth decreased on average to below LOQ for the sensitive method applied (0.005 µM), and only 6 of 23 subjects still had measurable levels (all < 0.04 µM). In the group randomized to drinking, all subjects had levels > LOQ after 3 months but average PEth did not change despite higher intakes by a factor of 1.6–56 according to baseline interviews. AUROC for qualitatively discriminating between the two groups at 3 months was 92% (82–100%). This study shows that PEth has a good ability to discriminate abstainers from moderate drinkers and that 0.05 µM is a reasonable cutoff although larger studies would be needed to ascertain that higher levels are not observed in a small minority of abstainers [88], especially among subjects with reduced kidney function. Along the same line, studies from Sweden categorize subjects with levels below 0.05 µM in blood as “abstainer” 0.05–0.3 µM as “moderate drinkers” and > 0.3 µM as “overconsumer” [93, 94]. Current evidence does not indicate that PEth is formed at different rates in men and women [95, 96].

In a recent randomized and highly controlled experimental study, healthy volunteers were provided with either 0.25 or 0.5 g ethanol/kg body weight (1–3 drinks in 15 min) after only 1 week of abstaining; measurable levels in the whole blood were evident in all volunteers after alcohol intake and was observable until 14 days later in most subjects [90]. In a similar study done by the same research group, doses of 0.4 or 0.8 g ethanol/kg body weight were administered (2–5 drinks in 15 min) [95]. Background levels and a proportional dose–response increase were observed, no sex difference in PEth homolog pharmacokinetics were found, and PEth 16:0/18:2 synthesis was higher than PEth 16.0/18.1 at both doses; however, the mean half-life of PEth 16.0/18.1 was longer than that of 16.0/18.2 (7.8 ± 3.3 days and 6.4 ± 5.0 days, respectively) [95]. These studies indicate that moderate alcohol intakes over a short period affect PEth in all subjects but with large variations between individuals, especially at higher doses. This was also reported previously by others [97] and has even been observed experimentally in primates [98]. Individual measurements may therefore not accurately reflect the consumed amount of alcohol, even in a very controlled setting of high intakes over a limited time span.

Quantitation of PEth has improved much in sensitivity in recent years, and several studies have investigated levels even in pregnant women. In three studies, 1.4–40% may not be abstinent as determined by PEth at the end of the first trimester, depending on the population and analytical sensitivity [99–101]. Few studies exist at low to moderate consumption levels using high-sensitivity analytics, but subject-reported intakes correlate with blood PEth [90, 100]. In a study using a new highly efficient ultrasound-assisted dispersive liquid–liquid microextraction procedure, PEth dose–response was observed in groups reporting alcohol intake levels from 14–98 g/week, 98–210 g/week, or > 210 g/week. Dose–response was presented as differences between the three group averages and indicates considerable overlap between individual levels at these three intake levels [102]. While abstainers are often below the detection or cutoff level for PEth [103], and many social drinkers have non-detectable PEth with current methods [97, 103] up to a few percent of subjects reporting to be abstaining seem to have low but measurable levels of PEth in their samples [99]. This is likely due to incorrect reporting of intakes. Recent PEth measurements have a good concordance with other biomarkers at chronic high alcohol intakes and seem more sensitive than older methods [86, 89]. High PEth (> 0.3 µM), indicating heavy alcohol consumption, is also 95% concordant with blood EtG > 100 ng/mL; however, at PEth levels indicating moderate alcohol intakes (0.05–0.3 µM), concordance with EtG (> 1 mg/L) is only 56% [104]. Formation and degradation of PEth have been investigated over 7 days with blood samples that were either negative for PEth, added with ethanol, or positive for PEth [63]. Formation of PEth was observed at 37 °C and − 20 °C, peaking after 4 days and then decreasing, while a linear loss of PEth with time was observed at 25 °C, reaching approximately 40% at 7 days. Stable levels over 7 days were observed at 4 °C. Further studies are needed to investigate the potential loss of PEth during long-term sample storage at − 20 °C or − 80 °C.

In conclusion, with highly sensitive analytical methods PEth is a sensitive and specific marker of ethanol intake at levels as low as a single alcoholic drink with an extended time window of days or weeks after intake, but inter-individual variations are high after single as well as repeated doses. PEth seems useful in studies of high drinking levels but may also prove useful for estimating the average intakes in groups of social drinkers; further studies to verify this should include additional repeated sampling in a controlled study of low-responders to PEth and of reported alcohol abstainers having positive blood PEth.

#### Fatty acid ethyl esters

Alcohol also interferes with lipase activity, substituting for aliphatic alcohols that esterify fatty acids. This results in the formation of fatty acid ethyl esters (FAEEs), i.e., a class of neutral lipid products [105]. FAEEs are formed by cellular synthesis, e.g., by mononuclear blood cells, directly from ethanol at physiological doses [106], and formation is likely to be directly proportional to the individual total BAC over time, given by the area under the BAC curve (AUC) [107]. FAEEs are stable at 4 °C or below for at least 48 h [107]. FAEE stability has been investigated in a 7-day storage experiment with blood samples that were either negative for FAEE, negative but added with ethanol, or positive for FAEE [63]. In the negative samples, FAEE was formed at 25 °C and 37 °C. Addition of ethanol to negative samples strongly increased FAEE formation at these temperatures. Formation of FAEE was also observed in the positive samples where FAEE increased at 37 °C up to 5 days, followed by degradation. Formation increased also up to 4 days at 25 °C and remained stable until 7 days, while FAEE in the positive samples was stable at 4 °C and − 20 °C for 7 days. These results indicate that sampling and storage are crucial for the analysis of FAEEs and that formation as well as degradation may distort results.

Peak serum FAEE concentrations may be around twice as high in men compared to women at the same blood alcohol concentration, indicating that the AUC for BAC rather than peak BAC reflects FAEE formation, while dosing rates (drinking within 2–90 min) had little effect on kinetics [108, 109]. In a single-dose study with alcohol doses from 6 to 42 g in healthy young men, the characteristics of the most abundant FAEEs (palmitic, oleic, and stearic acid ethyl esters) were showing initial kinetic properties similar to plasma EtG with peak formation within 30–60 min, clear time- and dose–response relationships, and a time window for detection in the blood plasma of 3–6 h [45]. The fractional formation (or rate of degradation) of FAEEs was dependent on the dose, indicating non-saturated kinetics for the enzymes involved in FAEE metabolism; while *C*_max_ for FAEE was almost linear after single doses of 6–42 g alcohol, the AUC was almost fourfold higher on average at the highest compared with the lowest dose, and inter-individual variation also increased with dose [51]. These results would indicate that FAEE degradation rather than its formation may be affected by saturation kinetics. After binge-drinking 64–172 g alcohol, background serum FAEE was reached 15–40 h later [59, 60]. Again, inter-individual variation was large [59]. After chronic high intakes, FAEEs can be observed in the blood for a much more extended period [110], even up to 99 h [60]. This may be seen as additional evidence that FAEE elimination or excretion may show saturation kinetics, being compromised in alcoholics; this might be due to the alcohol-induced effect on blood lipids, but studies differ on whether other blood lipids do [108] or do not [60] affect FAEE. Serum albumin has been shown to affect FAEE levels significantly, possibly by affecting FAEE transport [111]. FAEE above background levels may also be measured in dried blood spots collected up to 6 h after high doses of alcohol [112]; however, this technique has not been investigated at moderate or low doses.

FAEE in hair has been investigated to a considerable extent. Levels increase with chronic intake levels [70, 113]; however, individual variation in hair FAEE is considerable with a large overlap between subjects claiming no, moderate, or high habitual intakes [70, 113, 114]. This variability includes null as well as high levels in hair from some subjects in all three groups. Analysis of hair segments indicates similar but highly individual profiles; further comparison of FAEEs on the hair surface or the inner parts of hair indicates that FAEE enter into the hair from hair sebum [113]. FAEE in hair from different body locations has been shown to correlate, albeit with large variations within and between subjects [115]. In one study, the authors found no correlation between FAEE and EtG in hair [70], indicating that the incorporation of these compounds may be affected by different biochemical or physiological processes. FAEE was measurable in all hair samples using sensitive analytical techniques, even in children’s hair [70]. FAEE has also been detected in sebum collected by skin wipe tests showing that teetotalers and social drinkers were not different; however, heavy drinking affected skin sebum levels [116]. These findings indicate that endogenous formation pathways for FAEE may potentially exist.

FAEEs are sensitive to hair products containing alcohol [117], and a negative test for FAEE in the serum or EtG in serum or urine along with positive FAEE or EtG in hair is regarded as reflective of hair product use [114]. In 8% of cases negative for FAEE, EtG may be measured in hair, which is likely to reflect the potential presence also of EtG in some hair products [114]; this might indicate that a non-trivial percentage of cases positive for both EtG and FAEE in hair might be artifacts due to the use of several hair products and hence not reflective of alcohol use. Hair FAEE may also be affected negatively by shampooing and potentially by other hair products, which could potentially extract FAEE from the hair [117]. However, in large cross-sectional studies among forensic cases, neither body composition nor any use of hair wax, grease, oil, gel, or spray had any major effects on hair FAEE [118, 119]; instead, bleaching and/or dyeing reduced hair FAEE. Higher levels of FAEE as well as EtG were observed in abstainers than in moderate drinkers within this target group; this observation was ascribed to misreporting [119].

In conclusion, FAEE is formed readily from ethanol by lipases, apparently in a dose–response fashion related to the area under the BAC curve; this curve is known to vary between individuals, but transport, degradation, and excretion of FAEE may also depend on blood levels and on drinking habits, leading to large inter-individual differences in the kinetic behavior of FAEE measurements. Heavy drinking leads to delayed FAEE clearance; however, in moderate drinkers, plasma or serum FAEE levels decrease to baseline at a time point between those of BAC and EtG. Hair FAEE seems to be observed at levels above LOQ more readily than hair EtG and is practically always detected by sensitive methods, even for teetotalers, including children. This might indicate the presence of external or endogenous sources or of measurement errors that are still not explained. However, a large, strictly controlled study is still missing on FAEE in the blood as well as hair, especially investigating the levels in teetotalers and light to moderate drinkers.

#### 5-Hydroxytryptophol and related metabolites

A few other markers should be mentioned here since they have been applied for the “direct” measurement of steady-state alcohol intake. These are metabolites formed at an altered rate following high ethanol intake, namely a decrease in 5-hydroxyindole-3-acetate (5-HIAA) and an increase in 5-hydroxytryptophol (5-HTOL); the latter is measured in more recent studies as its glucuronide (5-HTOLG), which is more abundant [120, 121] in the urine. The ratios of 5-HTOL:5-HIAA or 5-HTOLG:5-HIAA as well as the ratio 5-HTOL to creatinine in urine have been shown to peak 4–6 h after a single dose of 0.8 g/kg alcohol (high intake). The ratios stayed above baseline until 16–26 h later [122] thereby forming a marker of recent high alcohol intake with an excretion time window of urine ranging between that of ethanol and of EtS or EtG [50]. Little investigation has been done on 5-HTOL at low to moderate intakes of alcohol or on the detailed kinetics of the marker at single or chronic intakes. The markers can therefore not be validated at moderate alcohol intakes.

#### Metabolomics investigations

Several studies have applied untargeted metabolomics (metabolite profiling) to discover and validate biomarkers of general alcohol intake by comparison with dietary instruments such as food frequency questionnaires [123]. In a study of 3559 female twins from the UK, who reported their alcohol intake by food frequency questionnaire (FFQ), increased levels of hydroxyvalerate, androgen sulfate metabolites, and several other endogenous metabolites were associated with alcohol, but no direct markers of alcohol intake were observed by the profiling technique [124]. In an NMR metabolomics study from Finland, 9778 young adults (53% women) with moderate alcohol intakes according to questionnaires were investigated; no direct markers of alcohol intake were observable but lipoprotein markers (e.g., HDL), phospholipids, androgens, and branched-chain amino acids associated with alcohol intake corroborating findings in other studies [125].

In other observational studies using metabolic profiling to investigate alcohol intake, EtG is frequently observed along with other metabolites associated with alcohol intake. In the Lung, Colorectal, and Ovarian Cancer Screening Trial, FFQ data from 1127 postmenopausal women (50% having breast cancer) were used to find serum metabolites associated with alcohol intake [126]; these included EtG and a large number of androgen steroid hormone metabolites as well as hydroxyisovalerate and 3-carboxy-4-methyl-5-propyl-2-furanpropanoic acid (CMPF) (a fish intake marker). A metabolite profiling study of 849 males and females from the PopGen study in Kiel, Germany, confirmed most findings from previous studies in the UK and the USA, showing EtG along with hydroxyvalerates, androgenic metabolites, and CMPF to be significantly associated with alcohol intake [127].

Some of the associations with alcoholic beverage intake may reflect the biological effects of alcohol, e.g., on lipoproteins and several lipid classes [128–130] or on steroid metabolism affecting androgens and estrogens [125–127, 131]. The associations may also reflect apparent confounders of alcohol intake such as fish [127, 129] coffee [129] or tobacco [132] related metabolites, or with specific alcoholic beverages (covered later in this review), but few besides EtG are likely to directly reflect alcohol intake. This is supported by the country- or sex-specific nature of the associations, for instance, none of the previously mentioned metabolite associations was observed in Japanese cohorts, where only men were included in the analysis [133, 134].

Mono- and dihydroxy-valeric acids have been observed in several studies [127, 129]; however, the cause of their association with alcohol has not been investigated extensively. Two reasonable explanations may be proposed: (a) some shorter- or branched-chain hydroxylated and branched-chained acids are oxidized metabolites of the side products (fusel) commonly formed during alcoholic fermentations or (b) alcohol intake affects branched-chain amino acid metabolism [135], leading to higher postprandial plasma levels and increased degradation into hydroxyvalerates. Further studies are needed in order to investigate these possibilities; if hydroxy-valerates result from fusel, they may prove useful in future combined markers to estimate intakes of specific alcoholic beverages.

#### Indirect measures of alcohol intake

Although these markers are not the primary subject of this review, they are shortly mentioned here because they are often used in the assessment of alcohol intake. Some indirect markers are in reality efficacy markers that may be affected by high, chronic alcohol intake.

Alcohol is acutely as well as chronically toxic to the liver, and hepatic enzymes such as gamma-glutamyl transferase (GGT), alanine aminotransferase (ALT), and aspartate transaminase (AST) therefore leak into the blood as part of the toxic response to high alcohol intakes [18]. This toxic response is useful to assess whether hepatic effects are found in association with alcohol intake, but the tests are not specific to alcohol since most other liver conditions also increase GGT, ALT, and AST [136].

Three markers of common use in alcohol research are the mean corpuscular volume of the erythrocyte (MCV), carbohydrate-deficient transferrin (CDT), and plasma sialic acid index of apolipoprotein J, all measured in the blood. Among these, the sialic acid seems to compare with liver enzymes [137, 138] while MCV is related to nutritional status [136], but none of them is relevant at moderate intake levels.

Daily use of alcohol is also associated with a number of more general biochemical and physiological effects even at light to moderate intakes (< 20 g/day), including an increase in high-density lipoproteins (HDL) and adiponectin, and at high doses also increased heart rate and higher blood pressure [139]. The most widely used marker among these is the increase in HDL cholesterol with alcohol intake, and this marker as well as its main apolipoprotein A1 (ApoA1) seem sufficiently sensitive at the group level to pick up contrasts of a single drink a day versus abstaining [140]. However, since not all subjects may react by increasing their HDL and since many other factors affect the level of this lipoprotein, the marker is most useful at the group level, i.e., to assess whether a change in alcohol intake is taking place in a group of subjects. None of the HDL subfractions seems to respond differently compared with total HDL or total ApoA1 [140].

While none of the indirect measures of alcohol intake is specific or very sensitive, attempts have been made to combine them into a multivariate model to predict moderate vs. high intakes of alcohol. The so far best-investigated model is the Early Detection of Alcohol Consumption (EDAC) score combining 36 routine clinical chemistry and hematology markers that may to some extent be affected by daily alcohol intake. The specificity for detecting problematic daily alcohol intake levels was found to be above 90% for both males and females by EDAC; however, the sensitivity in the first published study was quite low, below 50% [141]. Subsequent testing in much larger sample materials has confirmed higher specificity and reported sensitivities of 70–85%, resulting in overall AUROC values ranging from 80 to 95% [142, 143]. The EDAC score is well validated with receiver operating characteristics (ROC) of around 0.95 for identifying heavy drinkers [35]. However, this categorical tool cannot be used for a more accurate assessment of recent or longer-term light or moderate alcohol intake and is not useful for alcohol intake assessment in nutrition studies.

In conclusion, these markers and classification tools are not tabulated as valid biomarkers within moderate intakes in Table 1 but are listed among disregarded markers in Supplementary Table S3.

#### Marker validation

Candidate and established markers of moderate alcohol intake are listed in Table 1 along with their validation by eight validation criteria, while markers that are not able to reflect such intakes are listed in Supplementary Table S3. Among ethanol/alcohol biomarkers, ethanol has been validated for dose- and time response and is also broadly used due to good analytical performance, robustness, reproducibility, reasonable stability, and reliability. The drawbacks are considerable inter-individual variability in response after a given dose, and a short half-life resulting in a narrow time window of detection. Methanol is formed by several endogenous processes and degradation is inhibited by ethanol at higher doses. Dose- and time-response is therefore only seen at higher chronic intake levels or after binge drinking, and methanol is not a valid marker for moderate doses of ethanol. The robustness is weak due to variable other sources of exposure but the analytical performance by GC is well-established and reproducible.

Acetaldehyde might potentially be an ideal marker of long-term intake but is not extensively investigated. As a primary metabolite of ethanol it is plausible but there are no established and validated analytical methods, dose- and time-responses are not well known, and robustness is challenged by exposures from other sources, including endogenous formation; moreover, acetaldehyde stability, reliability, and reproducibility seem to depend on the analytical approach, or are simply unknown.

EtG in the blood or urine is analytically well established, quite reliable, and reproducible; however, formation kinetics varies between individuals. It is stable at low temperatures, robust, and dose- and time-response are well validated at moderate and high single or repeated doses. The major weakness of this marker is the large variability in response at low alcohol intakes and an unknown source of background in some subjects. EtG in hair is more variable between subjects having similar intakes than blood or urine EtG, and its robustness is affected by hair products; dose–response seems fair at higher intakes, but time-response is complex due to hair growth and loss of EtG due to wear and tear, including hair wash. The analytical performance is well documented.

EtS is another direct phase 2 metabolite of ethanol (hence plausible) and very similar to EtG in terms of all performance parameters but causes of low formation in some subjects is unexplained. EtS may be observed at slightly lower alcohol intakes compared to EtG, but this needs further verification. EtS in hair is not yet well documented.

FAEEs in the blood are apparently proportional to the AUC for alcohol in blood; however, formation seems higher in men than in women. The rate of FAEE degradation in the blood varies between individuals, and FAEE is also unstable in blood samples at temperatures above 4 °C. Biological degradation is much delayed in heavy drinkers, strongly distorting the time-response curve at higher regular intakes; this may be used to identify problem drinking but reduces the applicability of the marker as a BFI for alcohol intake in studies where alcohol abusers may be among participants. Due to the high inter-individual variation, FAEE dose–response only gives a rough estimate of the intake level with considerable misclassification at the individual level. FAEE in hair is a promising marker for the estimation of longer-term intake levels; however, the variation between individuals seems even larger, and background levels are therefore highly variable, so more investigation will be needed in order to understand the biology behind high variability and background levels to further develop and evaluate the appropriate use of this marker.

Blood and dried blood spot PEth are still methods under development, resulting in some heterogeneity in the literature regarding the levels observed [144, 145]. PEth is clearly dependent on the activity of phospholipase D, leading to considerable inter-individual variation. PEth stability and formation in the samples may be an issue, and so are the effects of drying the blood and keeping the blood spots at room temperature [63, 146, 147]. The most sensitive methods for PEth analysis also reveal individual variability but at the same time indicate that background levels are low for the majority of subjects. Individual levels after extended periods of abstaining or low intakes are still missing in the literature and reliability in terms of relationships with actual doses are not sufficiently investigated at lower doses.

### Beer

Beer is one of the world’s oldest drinks [148] and the most widely consumed alcoholic beverage [149]. It is a very complex beverage comprised of thousands of compounds such as oligosaccharides, amino acids, nucleotides, fatty acids, and phenolic compounds [150, 151]. Traditionally, the basic ingredients of beer are water, sprouting cereal grains, yeast, and boiled hops (wort) as raw materials; their transformation products formed during malting and fermentation are suggested as a source of potential candidate beer intake biomarkers. Barley is the most commonly used cereal, though wheat, maize, and rice are also used, mainly as an addition to barley. The appearance and flavor of the beer are affected not only by the type of cereal grain but also by many other parameters such as the type of malting process, temperature, fermentation type, mashing, and the variety of hops used for the wort. The wort provides highly characteristic components to the beer imparting bitterness, odor, and aroma. Some of the characteristic phytochemical constituents of hops are α-acids, β-acids, and prenylated chalcones such as xanthohumol (XN) [152]. These compounds may not be specific to beer intake since hop products are also consumed as herbal remedies; however, upon boiling of the wort, the α-acids are isomerized and degraded forming other chemical structures, iso-α-acids (IAAs), that are only found in beer. Therefore, compounds produced from the rearrangement of hop constituents can be suggested as plausible candidate beer intake biomarkers.

#### Iso-α-acids and reduced iso-α-acids

IAAs exist in three predominant analog forms (isohumulones, isocohumulones, and isoadhumulones), and each of them is also present as diastereoisomers [153]. The *cis:trans* ratios of IAA (usually ~ 2.2:1) is influencing beer bitterness [154, 155]. Rodda et al. (2013) suggested IAAs and reduced IAAs as biomarkers of beer intake [153]. They could quantify *trans*-IAAs and qualitatively monitor *cis*-IAAs in plasma at 0.5 h and up to 2 h after beer intake in a pilot study with one subject [153]. Postprandial studies investigating the excretion profile of IAAs after beer intake revealed a rapid absorption of IAAs into plasma (*T*_max_ 30–45 min), compared to the excretion profile in urine that typically shows a peak between 90 min and 3 h [156, 157].

Despite their specificity for beer, the potential applicability of IAAs as quantitative biomarkers of beer intake is limited by their instability since their quantity varies during storage [152, 158]. The degradation is strongly dependent on the stereochemistry of the IAAs. *Trans*-IAAs are degrading faster than *cis*-IAAs, leading to the formation of tri- and tetra-cyclic compounds during storage. In urine, oxidized degradation products such as mono- and di-hydroxylated humulones have been observed both for *cis*- and *trans*-IAAs [159]. An untargeted LC–MS-based metabolomics study revealed many of the oxidized excretion products in urine following a single drink of alcoholic or non-alcoholic beer in a cross-over design. None of the IAAs were detected in a pilot validation study with a low-hopped beer variety, underpinning the limitation of IAA metabolites as a reliable marker only for hopped beer intake [156]. This suggests that the IAAs in low-hopped beers are completely degraded or present at too low levels for detection and use as BFI.

Reduced IAAs, namely rho-IAA, tetrahydro-IAA, and hexahydro-IAA, have also been proposed as promising beer biomarkers. Reduced IAAs are light-stable synthetic derivatives of IAAs; they are usually added to hops to avoid light-induced degradation of IAAs resulting in undesirable (stall) aroma of beers bottled in clear or green bottles and hence, subject to light exposure [160]. In one study, the levels of IAAs were found to be lower or insignificant for clear (or green) bottled beers [161]; measures to stabilize their flavor and bitterness can therefore be taken, such as the addition of reduced IAAs or a high content of isocohumulone [162]. The total level of IAAs together with reduced IAAs has been suggested as a combined qualitative beer intake biomarker with a specificity of 86% in the plasma of post-mortem specimens [161]. However, further validation studies are needed for more general use.

In addition to IAAs and reduced IAAs, an oxidation product of α-acids called humulinone has been proposed as a biomarker of beer intake based on LC–MS profiles of urine collected after 4 weeks of beer consumption in an intervention study [163]. Even so, humulinones are not only minor biotransformation products of α-acids but their concentration in beer is also shown to be diminished with longer-term storage, leading to the formation of other compounds [152, 164]. This might reduce the potential usefulness of these compounds as biomarkers.

In terms of bioavailability, oral administration of IAAs to rabbits leads to recovery of less than 6% of the dose in urine and feces, suggesting that their metabolism potentially goes through phase I and II reactions [165]. Incubation of IAA with rabbit microsomes demonstrated cytochrome P450 catalyzed oxidation and transformations of IAA with the formation of many compounds. Oral administration of IAAs to rabbits did not show any indication of direct glucuronidation or sulfation [166], yet phase II metabolism takes place through cysteine and methyl conjugation of oxygenated IAAs as demonstrated in urine metabolic profiles following beer consumption [156].

#### Isoxanthohumol

Other hop components, named prenylated phenols (isoxanthohumol (IX), 6-prenylnaringenin (6-PN), and 8-prenylnaringenin (8-PN), and XN), have been widely investigated due to their biological activity and potential health effects [167–169]. In line with the formation of IAAs, IX is formed through the cyclization of XN during wort boiling. The most abundant prenylated flavonoid in beer is IX (3–6 µmol/L) whereas XN, 6-PN, and 8-PN are comparably minor constituents (~ 0.03 µmol/L) [152]. More importantly, 8-PN is also formed through the conversion of IX by the intestinal microbiota [170] or through the cytochrome P450-catalyzed O-demethylation [171]. Therefore, the concentrations of 8-PN and IX in body fluids depend not only on their amount in beer consumed but also on host factors, i.e., their potential biotransformation [172, 173].

IX is not yet documented to come from any other dietary source than beer or hop extracts. Quifer-Rada et al. (2013) developed a LC–MS method for the analysis of IX, XN, and 8-PN to qualify beer consumption in a single-dose drinking study with 10 subjects [174]. Eight hours after the consumption of a single moderate dose of beer, spot urine samples showed a significant increase only for the IX concentration in all subjects. Surprisingly, 8-PN was also detected in a spot urine after 4 days of a wash-out period in all subjects. Therefore, a delayed conversion of IX to 8-PN has been proposed [18, 19] and may indicate the usefulness of these compounds to assess either very recent (IX) or past intakes (8-PN) within several days; further studies are needed to investigate the kinetics of 8-PN excretion.

IX has also been evaluated as a urinary BFI for beer in three different trials [175]. In a dose–response, randomized, cross-over clinical trial a linear association between beer dose and IX was observed in male volunteers, while IX among females showed individual saturation kinetics of excretion. Inter-individual differences in the conversion of IX to 8-PN by the intestinal microbiota have been previously reported [169] and could be an influencing factor contributing to the saturation kinetics in females. In a second randomized cross-over intervention trial with 33 males consuming beer, non-alcoholic beer, or gin for 4 weeks, suitability of IX as a qualitative biomarker of beer intake in men was evaluated. The prediction of beer intake (beer and non-alcoholic beer vs. gin) achieved a sensitivity and a specificity of 98% and 96%, respectively. Lastly, beer intake data, recorded by a validated food frequency questionnaire, from a randomly selected subgroup of 46 volunteers participating in the PREDIMED cohort was assessed resulting in a 67% sensitivity and a 100% specificity. The low sensitivity was justified by the large range of beer intakes (22–825 mL/day), although some low-volume drinkers in the group could also have been misclassified as non-beer drinkers. The analytical method has subsequently been used to assess volunteer’s compliance in two additional beer interventions [163, 168]. The authors reported an increase of IX in 93.5% of collected urine samples from both intervention groups, drinking beer or non-alcoholic beer, respectively [168].

In a subsequent paper, Quifer-Rada et al. (2014) concluded that IX is a specific and accurate biomarker of beer intake [175]; however, others have pointed out that this result did not take into account the previously demonstrated extensive glucuronidation of prenylflavanoids [176]; other authors applied hydrolysis of glucuronides in the urine prior to analysis to calculate the total prenylflavanoids excreted [170, 177]. Recently, Daimiel et al. (2021) measured plasma and urinary levels of IX and 8-PN by treating the samples with a mixture of β-glucuronidase and arylsulfatase to liberate any conjugated IX and 8-PN [178]. As expected, urine IX concentration was higher after beer and non-alcoholic beer intake compared with both washout periods, while an increase in plasma IX was only found after alcoholic beer intake. Furthermore, the stability of 8-PN in urine after beer consumption and in plasma after beer and non-alcoholic beer interventions suggests that the compound is useful as a beer intake biomarker. Breemen et al. (2014) studied the profiles of 8-PN, 6-PN, IX, and XN and their conjugates in serum and in 24 h urine samples from 5 women following a boiled spent hops extract intake [176]. In serum, the half-life of IX and 8-PN (free and glucuronide conjugated) are up to 24 h and > 24 h, respectively in different individuals [176]. One of the findings was a large inter-individual variability in the excretion profiles related to the conversion of IX to 8-PN. This may complicate the applicability of IX as a single quantitative biomarker of beer intake for both men and women. Furthermore, prenylflavanoids behave differently from most polyphenols since they are unstable at acidic pH. Therefore, a specific analytical method must be applied to determine them in biofluid samples after beer consumption [174], potentially complicating the use of these markers in multi-marker methods.

#### Hordenine and its conjugates

Besides compounds originating from hops, germinated barley contains hordenine (*N*,*N*-dimethyltyramine), which has also been suggested as a biomarker of beer intake [179]. Hordenine is produced during the germination of barley and transferred to beer from malted barley. The appearance of tyramine methyltransferase activity during germination associates with the accumulation of N-methyltyramine, a precursor of hordenine [180]. Thus, products made with ungerminated barley such as barley bread do not contain hordenine. Steiner et al. [179] developed a LC–MS method for the quantification of hordenine in a drinking study with 10 subjects drinking either beer or wine. The results demonstrated the detection of hordenine in serum samples only after beer consumption. Hordenine concentration in serum varied according to the type of beer consumed and its hordenine content. After beer intake, the serum profile implied total removal of hordenine by 2.5 h, but only one subject was profiled [179]. Sommer et al. also evaluated free hordenine and its conjugates in plasma as beer intake biomarkers [181]. The concentration of free hordenine reached its peak 30–90 min after the beginning of the exposure and then rapidly decreased. Part of the free hordenine was biotransformed into glucuronide and sulfate conjugates immediately after its absorption. Hordenine sulfate *T*_max_ was between 90 and 150 min, while hordenine glucuronide *T*_max_ was 150–210 min in plasma. Urinary excretion peaked at 2–3.5 h after beer consumption but was still detected after 24 h [181]. In another study, hordenine in urine reached its maximum excretion into urine already at 0–1.5 h following beer intake [156]. However, hordenine was also detected prior to beer intake in some subjects, albeit at lower levels, indicating non-compliance, very long excretion half-life for some subjects, or intake of hordenine through consumption of other barley germ-containing foods or other food sources [156, 182]. Further studies are needed to evaluate the potential use of hordenine as a biomarker of beer intake. In particular, it should be assessed whether the concentration is sufficiently high for beer intake compared to the consumption of other foods, potentially containing barley germs or other confounding food sources, such as bitter orange or certain dietary supplements [5, 183].

#### Metabolomics investigations

Quifer-Rada et al. [163] investigated urinary metabolomics profiles following 4 weeks of intervention with beer, non-alcoholic beer, or gin. The authors proposed humulinone and 2,3-dihydroxy-3-methylvaleric acid as potential novel biomarkers. However, based on the established standard procedure for the identifications of metabolites in untargeted metabolomic studies [184], the identification of the latter was at level 2. The authors suggested that 2,3-dihydroxy-3-methylvaleric acid may be a product of fermentation, i.e., a *Saccharomyces cerevisiae* metabolite, and this is corroborated by several observational metabolomics profiling studies; however, also wine is fermented by *Saccharomyces cerevisiae* and several hydroxy-valerates have been found to associate with intakes of beer, wines, and total alcohol [127, 129]. Therefore, further studies are needed to confirm the specificity of 2,3-dihydroxy-3-methylvalerate as a biomarker of beer intake.

Another untargeted metabolomic study investigated the immediate effect of beer intake on urinary and plasma LC–MS profiles [156]. Many of the compounds associated with beer were originating from hops, yet those were either oxidation products or IAAs and as mentioned previously their level may change with storage. Other compounds were originating from wort, fermentation, or human metabolism of IAAs. Although those were clearly upregulated with beer intake, they were also present at least in some of the baseline samples. Therefore, a combined biomarker model was proposed [156]. For the aggregated beer intake biomarker, IAAs, and their major degradation products, tricyclohumols, were selected as hop metabolites, a sulfate conjugate of N-methyl tyramine (a hordenine precursor) as a barley metabolite, pyro-glutamyl proline as a product from the malting process and a compound putatively identified as 2-ethyl malate, as a known product from the fermentation. The combined biomarker model from 24 h pooled urine samples of 19 subjects was validated against an independent study with four subjects in which they consumed two different types of beer. The biomarker model predicted all the samples collected up to 12 h correctly (AUC = 1). This proposed biomarker model still needs to be validated in other studies with an observational setting to confirm robustness.

#### Marker validation

Among the beer biomarker candidates, IX has been investigated for many different aspects of validation. The major issues for the potential application of IX as a biomarker of beer intake are its conversion to 8-PN in the gut, the extensive glucuronidation, and the interindividual and potentially sex-dependent variation in excretion kinetics. Instead of using only IX, a combination of IX, 8-PN and their conjugates might be a promising approach as a qualitative biomarker of beer intake. Stability is the main concern for IAAs, therefore their combination with the reduced IAAs is also promising. Hordenine may not be specific to beer, thus further studies are required to evaluate its excretion in relation to other foods. The combined biomarkers approach is a highly promising tool for beer intake but still needs validation in observational studies. The assessment of the candidate beer intake biomarkers by the full set of validation criteria can be found in Table 1.

### Cider

Cider is a beverage obtained from the alcoholic fermentation of apples or pears. It is very popular in the UK, which is also the largest producer and consumer in the world. Cider is also consumed in other European countries, such as Spain, France, Ireland, and Germany, and low- or non-alcoholic versions are common soft drinks in some countries, including Sweden. According to the European Cider Trends 2020, cider consumption in Europe from 2015 to 2019 is roughly 4 L/capita/year (from 0.15 L/capita/year in Russia to 14 in the UK) [185]. In recent years there is a gradual but constant increase in cider consumption [185], probably due to the consumers’ appreciation of its low alcoholic content and because it is perceived as natural, genuine, and healthy.

To date, there are no untargeted metabolomics studies investigating the metabolic effect of cider consumption, while two pilot studies have used targeted approaches to identify specific cider polyphenolic metabolites [186, 187]. In the first study, 6 human subjects consumed a high single dose of cider (1.1 L), and polyphenolic metabolites were searched in plasma and urine samples after β-glucuronidase and sulfatase treatment [186]. Low levels of isorhamnetin (3′-methyl quercetin), tamarixetin (4′-methyl quercetin), and caffeic acid derivatives were found in human plasma after hydrolysis of conjugates, while hippuric acid and phloretin were found in urine. The second study was focused on the metabolism of dihydrochalcones, which are phenolic compounds distinctive of apple and apple products [187]. In this study, 9 healthy subjects (21–42 years old) and 5 subjects with ileostomy (40–54 years old) received a single dose of cider (500 mL), and the main metabolites found in plasma, urine, and ileal fluid were phloretin-glucuronides and phloretin-glucuronide-sulfates. The main metabolite in all biological samples was phloretin-2′-*O*-glucuronide, having a *T*_max_ in plasma of 0.6 h and accounting for 84% of the cider-related metabolites found in the urine of the volunteers [187].

With the exception of phloretin derivatives, which are specific to apple products, other putative biomarkers identified such as hippuric acid and quercetin metabolites are unspecific and relate to almost any intake of fruit or vegetables. In fact, they have been already identified after consumption of other foods rich in polyphenols [188–191] and have been suggested as possible dietary biomarkers of total fruit and/or vegetable consumption [192, 193]. Phloretin and phloretin conjugates are found in urine after consumption of apples and apple products [194], including cider [195]. Human supplementation studies demonstrate that single doses of apple or apple juice, as well as cider, determine the appearance of phloretin derivatives in plasma and urine [196]. However, phloretin derivatives have been detected in human urine also after grapefruit juice and orange juice consumption, either as a result of naringenin metabolism or adulteration [197]. Moreover, phloretin excretion determined in 24 h, in overnight or in morning spot urine samples, has been suggested as a short-term dietary biomarker of all fruits, of fruit juice consumption, and/or apple consumption [198–202]. Without additional markers representing the apple fermentation or the ethanol content to form a combined biomarker, the phloretin metabolites would not seem generally suitable as biomarkers of cider intake. In conclusion, there are not many studies investigating biomarkers of cider intake and none of the suggested biomarkers appear to be adequate or specific to cider intake.

### Wine

Wine is a common beverage consumed in Mediterranean countries, obtained through the fermentation of grape must. Mediterranean diet has been defined by low to moderate amounts of red wine often accompanying main meals, among other dietary factors [203, 204]. The basic ingredients of wine are water, grapes, and yeast as raw materials and their transformation products formed during maceration and fermentation [205]. Generally, the ethanol concentration in wine ranges between 10 and 13%. More than 500 compounds have been found in wine, derived primarily from the few compounds that occur individually at high concentrations. The main compounds that occur at high concentrations are water, ethanol, organic acids, sugars, and glycerol. Those are primarily responsible for the taste and mouthfeel. Besides, phenolic compounds are an additional large and complex group of compounds of particular importance to the characteristics and quality of wine [206]. Polyphenols from wine can be divided into two primary groups: flavonoids and non-flavonoids. Red wine is around tenfold higher in polyphenolic content than white wine [204]. Due to the maceration during red wine production, extraction of color and other substances from grape skin and seed occurs, so that polyphenolic compounds in red wine increase. Colorless and filtered grape juice is used during white wine alcoholic fermentation, so that contact with grape skin is avoided [205].

#### Resveratrol and its conjugates

3,4′,5-Trihydroxystilbene, commonly known as resveratrol (RV), is a natural stilbene present in grape and grape products. They are the primary sources of dietary stilbenes, especially in red wine [191]. During the red wine-making process, skin and seeds, which are the RV richest parts of the grape, are macerated and stay in contact with the alcohol formed during the fermentation. Both processes facilitate the extraction of RV and explain why red wine contains more stilbenes and other polyphenols than white wine [207]. RV and its derivatives can also be found in minor concentrations in some nuts (e.g., peanuts, pistachios), berries, and dark chocolate [208].

RV can be found as diastereoisomers that coexist in plants as well as in wine, although the *trans* isomer appears to be the more predominant and stable natural form [207, 209]. RV has been widely studied for being a biologically active molecule; however, its bioavailability is limited due to rapid metabolism after absorption [210]. Indeed, metabolites are the primary circulating forms [211]. Metabolism of RV in humans involves the formation of glucuronides and sulfate conjugates of the RV absorbed in the small intestine [210, 212]. The unabsorbed RV reaches the colon and is converted into dehydroresveratrol (DHRV) by the microbiota [213]. Total RV glucuronides have been reported to be a putative intake biomarker of wine consumption [214], but ignoring part of RV metabolism with this approach may limit its applicability. Other authors have used enzymatic hydrolysis of conjugates to liberate RV as a wine intake biomarker [215–217].

Strategies to increase RV bioavailability have been evaluated in several single-dose studies [212, 214, 215, 218, 219]. In these studies, RV conjugates have been confirmed in plasma, serum, and urine after wine consumption [212, 214, 215, 218–220]. Rotches et al. (2012) reported 17 metabolites including conjugates of RV, piceid, and DHRV in human biological samples after red wine intake [221]. The main RV phase II metabolite found in plasma and urine was *cis*-RV-O-glucuronide, with a *C*_max_ ~ 2–6 times higher than the other glucuronides at 2–2.5 h after the wine consumption. RV glucosides were rapidly absorbed and appeared around 1 h after the intervention, while phase II and microbial metabolites appeared between 0–8 h and 4–12 h, respectively [219]. Additionally, a high inter-individual variability was found in *C*_max_ and AUC of DHRV glucuronides, most likely due to a high heterogeneity in the microbiota between the participants [219]. RV metabolites have also been observed in human LDL particles after a single dose of 250 mL of red wine, indicating an affinity for lipoprotein particles [221].

Randomized, controlled, cross-over intervention trials over periods of 3–4 weeks have been performed to compare the effects of red wine, dealcoholized red wine, and gin [211, 217, 222]. Phase II derivatives of RV and microbiota-derived DHRV metabolites in 24-h urine samples were sensitive and specific to wine consumption, being a useful tool to evaluate compliance in the clinical studies thereby having a potential applicability for making associations between the intake of wine and biological effects [211, 217, 222]. In a comparative study between the 4 weeks of consumption of red wine or dealcoholized red wine, no differences between the interventions were observed in terms of concentrations of RV metabolites excreted [211]. More precisely, several combinations of different phenolic metabolites (mainly gallates) and RV metabolites (host and microbial) were shown to predict wine consumption with an AUC of up to 98% for urine samples and 91% for plasma samples with 4 weeks of red wine, gin or dealcoholized red wine intake [217]. However, the combined biomarkers have not been evaluated for robustness or for high and low red wine intake levels in cross-sectional studies, so further validation is needed. The marker combinations are independent of alcohol since dealcoholized red wine was detected just as well as the alcohol-containing wine.

Recently, González-Domínguez et al. (2020) optimized a multi-targeted metabolomic platform for the quantitative analysis of 450 food-derived metabolites by ultra-high performance liquid chromatography-tandem mass spectrometry (UHPLC-MS/MS) [223]. The putative biomarkers were validated by a 1-month intervention trial with a Mediterranean diet supplemented with 270 mL/day of red wine. The consumption of red wine was reflected by the detection of a significant increase in plasma of *cis*-RV-4′-sulfate, DHRV-3-sulfate, and ethyl sulfate [223]. Furthermore, differences between the changes observed in urinary RV concentrations after intake of red and white wines have been the subject of several studies [224–226]. The biomarkers were significantly better at detecting red than white wines, showing a limitation in the combined marker applicability for general wine consumption [224–226]. Additionally, urinary anthocyanin concentrations significantly increased after red wine but remained practically unchanged after white wine intake, being a specific measure of red wine intake and a promising group of biomarkers to differentiate red and white wine consumption [224]. However, anthocyanins are also found in other foods, particularly red and blue berries and intake of these foods may affect the specificity of combined measurements of RV metabolites and anthocyanins.

RV metabolites have also been tested as wine biomarkers in two large cohorts [226–229]. In the EPIC cohort, dietary RV and RV-3-O-glucoside intakes were estimated based on 24 h dietary recalls using the food content values of these two compounds reported in the Phenol-Explorer database [191], and compared to the measured levels in 24 h-urine samples collected on the recall days. Urinary excretion of RV was significantly and positively associated with wine intake [227]. In addition, using a metabolomic approach, red wine consumption was predicted with an AUC of 86.9% for DHRV glucuronide among a sub-sample of 418 subjects from the EPIC study [229]. As another example of a cross-sectional study, the correlation between a 137-item validated FFQ in 1000 subjects from the PREDIMED study and the concentration of RV metabolites excreted in morning urine has been studied. Drinkers and non-drinkers could be discriminated with a sensitivity of 93.3% and a specificity of 92.1%, and one drink of wine per week could be detected. Moreover, the concentrations of urinary RV metabolites of consumers of 3 glasses of wine/week were higher than those of the 1 glass/week consumers [228] at the group level. In a smaller study with 52 participants from the same cohort, those who reported wine consumption had significantly higher urinary concentrations of *trans*- and *cis*-RV-3-O-glucuronide than those who did not consume wine, and wine intake was predicted based on this marker with a sensitivity of 72% and a specificity of 94%. The percentage of false negatives was higher in those consuming wine intermittently than in those consuming it daily (43% and 24%, respectively) [226]. In another study, no correlation was found between data from a FFQ and the determination of free RV in plasma in a cross-sectional study with only 25 volunteers [230]. However, free RV is known to be rapidly absorbed and biotransformed. Therefore, RV metabolites seem to be a more precise objective measure of wine consumption in epidemiologic studies.

#### Tartaric acid

Tartaric acid or tartrate is one of the major components of red and white wine and the main component responsible for wine acidity [231]. Although it can be also found in other fruits, tartrate concentration is much higher in grapes or wine [232]. Indeed, tartaric acid has been proposed as candidate BFIs of grapes [233]. The only food source that presents similar amounts of tartaric acid is tamarind, a tropical sour fruit not commonly consumed in Western countries [234]. Tartrate is mainly found in the grape pulp and in much higher concentration compared to RV, leading to 14–20% of the ingested dose of tartrate excreted unchanged [235]. The applicability of tartrate as a BFI for wine consumption has therefore been assessed in wine interventions and observational studies.

Regueiro et al. (2013) developed a LC–MS method for the analysis of wine organic acids to qualify wine consumption in a single-dose drinking study with 5 subjects [236]. Ten hours after the consumption of 200 mL of red wine, spot urine samples showed a significant increase in tartaric acid concentration in all subjects [236]. Furthermore, a dose–response study has been conducted, showing that urinary tartaric acid concentration reflects the amount of wine consumed, and therefore allows to discriminate among levels of consumption in a male population [237].

Tartaric acid has also been evaluated by ^1^H-NMR, showing that it is the most discriminating metabolite in urine after dealcoholized wine as well as regular wine consumption in the setting of a prospective, randomized, controlled, cross-over trial [238, 239]. Additionally, 24-h urine excretion of tartaric acid after white wine consumption has been reported as useful in an intervention to evaluate compliance [240].

Recently, tartaric acid has been applied as an objective measure for wine consumption in a cross-sectional study of a sub-sample of postmenopausal women (60–80 years old) from the PREDIMED study [241]. After adjustments for several covariates (e.g., consumption of fruits, raisins), women who consumed more wine presented higher concentrations of tartaric acid in their urine [241]. Those who reported not consuming wine were excluded from the analysis, so background levels of tartaric acid were not reported [241]. However, a certain background of tartrate is commonly seen in a method validation study, 80 urine samples from 4 different subjects were analyzed in order to test the method. Tartaric acid was detected in 71 samples (67 above the limit of quantification, 68 ng/mL, but still very low) after a beer intervention study during which the volunteers were asked to abstain from other alcoholic beverages [242]. The background levels observed were only 0.1% of the average excretion seen in the previous studies after the intake of 300 mL of aged white wine [243]. In contrast, RV was not detected in any urine sample after the beer intervention [242]. Therefore, tartaric acid seems to be a promising quantitative biomarker of wine intake in epidemiological studies, although some noise can be expected due to the ingestion of low doses of this compound from grapes, raisins and other food sources.

Other authors proposed hydroxycinnamic acids that occur in white wine conjugated with tartaric acid (e.g., caftaric, fertaric) as putative BFIs. However, those compounds were detected in very low or undetectable levels in plasma [244], possibly due to fast hydrolysis in the human gastrointestinal tract.

#### Metabolomic investigations

Some studies have applied an untargeted metabolomic approach to obtain a holistic view of the metabolites associated with the intake of wine [127, 229, 238, 239, 245–247]. Other authors have opted for a targeted analysis to detect precursor wine compounds, intermediate metabolites, and end products [216, 217, 248, 249]. Those studies reported a wide urinary and blood metabolomic fingerprint of anthocyanins (e.g., malvidin glucoside), phenolic acids (e.g., gallic acid sulfate), hydroxybenzoic acids (e.g., methylgallic sulfate), stilbenes (e.g., RV metabolites), flavan-3-ols (e.g., epicatechin glucuronide), phenyl alcohol (e.g., hydroxytyrosol), or hydroxyphenylvalerolactones after wine consumption. In addition, syringic acid and 3-hydroxyphenylacetic acid in feces were correlated with red wine intake by a UPLC-ESI–MS/MS analysis in samples from 74 volunteers [249]. None of these fecal markers are regarded as promising biomarkers, see Supplementary Table S3.

Vázquez-Fresno et al. investigated urinary metabolomics profiles following a wine intervention study and also evaluated urinary metabolomics profiles associated with wine consumption in a free-living population [245]. A combined biomarker model using tartaric acid and EtG, showed an AUC of 90.7% and 92.4% in the intervention and in the observational study, respectively. Moreover, this combined wine biomarker model was applied to assess the time-response criterion, defining a timeframe of 2–3 days after the last glass of wine consumed to detect significantly higher amounts of both markers in wine drinkers in comparison to non-wine consumers [245]. This would indicate that tartrate together with EtG may be seen as an intermediate-term biomarker of wine intake, with good prospects for use in observational studies.

To determine the impact of moderate wine consumption on the overall urinary metabolome, samples from a red-wine intervention study (250 mL/day, 28 days) were also investigated by Esteban-Fernández et al. [246]. The 24-h urine was collected before and after intervention and analyzed by an untargeted UHPLC-QTOF-MS metabolomics approach. A total of 94 compounds linked to wine consumption, including specific wine components (tartaric acid), microbial-derived phenolic metabolites (5-(dihydroxyphenyl)-γ-valerolactones and 4-hydroxyl-5-(phenyl)-valeric acids), and several endogenous compounds with changed excretion levels in the urine [246].

#### Marker validation

Among the wine biomarker candidates, RV conjugates and tartaric acid have been investigated for many different aspects of validation. The major issues for the application of RV and its conjugates as wine intake biomarkers are that the content in wine is subject is highly variable and that human metabolism is showing inter-individual differences. However, dose–response in agreement with dietary instruments has been observed in observational studies indicating the validity of RV. Combining RV with anthocyanins might improve specificity for red wine but this needs further study. Tartaric acid seems to fulfill all the criteria for full validation, although a cutoff or correction may be needed for studies in subjects consuming other grape products, including raisins and fresh grapes. Both tartrate and RV metabolites may also be applied as grape and grape product BFIs and therefore will inherently lead to some misclassification when used as wine intake biomarkers. Another concern might be the presence of tartaric acid in some processed foods at relatively modest concentrations, due to its addition as an acidifying agent, and in high amounts in tamarind. Thus, its applicability might be limited to those countries where tamarind is not a commonly eaten food; the ratio of tartaric acid to RV might be an approach worth pursuing in future studies to separate alcoholic wine consumption from intakes of potentially interfering foods. For regular wine, the addition of alcohol biomarkers would further help discrimination from other grape products. Both RV and tartaric acid have been validated in wine interventions and in observational studies. The full validation criteria can be found in Table 1.

### Sweet wine

Dessert wine is any sweet wine, which is made from naturally fermented juice from fruit, generally grapes, and usually fortified with alcohol [250]. Sweet wine is often served with dessert or is the dessert itself. Some examples of dessert wines are sherry, port wine, some sweet sparkling wines, and sweet wines from Riesling grapes, picked late in the season to increase their sugar content. The percentage of alcohol is between 10 and 20% [251]. The higher levels of sugar and alcohol are obtained by different ways: (a) some grape varieties naturally produce high amounts of sugar; (b) by directly adding sugar or honey; (c) by adding alcohol, a process known as fortification; or (d) by removing water to concentrate the sugar [250].

Only three papers related to dessert wine intake biomarkers were found. In two studies, a sweet sparkling wine was used with the aim to identify general markers of alcohol consumption (i.e., EtG, EtS) and not specifically to find markers for the intake of sparkling wines [6, 252]. In order to determine biomarkers of wine consumption, including sparkling wine, measurement of *cis* and *trans*-RV-3-*O*-glucuronides was performed in urine and serum after supplementation of 10 healthy young men with 300 mL/day of sparkling wine for 4 weeks [226]. A significant increase of both isomers and of total RV metabolites was observed in urine (but not in serum), while RV aglycone, RV-3-*O*-glucoside and sulfate conjugates were undetected. The presence of *cis-* and *trans*-RV-3-O-glucuronides was also found after supplementation of white and red wine (200 mL/day) but not after gin supplementation (100 mL/day) suggesting that these metabolites can be considered specific biomarkers of grape wine intake in general, including the sweet grape wines.

In conclusion, the compounds so far identified represent very unspecific biomarkers. EtG and EtS are general biomarkers of alcohol intake [18] while RV is a biomarker of wine consumption [211, 217, 219, 225, 248] or of grape juice intake [253].

### Distillates and spirits

Spirit-based beverages are alcoholic drinks that contain at least 15% of alcohol. Such drinks can be produced directly by distillation of naturally fermented products with or without aromatizing substances; or indirectly by the addition of other alcoholic beverages, ethyl alcohol, or a non-alcoholic drink to the spirit-based beverage. Many categories of spirit-based beverages with clearly defined characteristics exist, as well as a classification based on their geographical origin. Most are distillates based on fermentation products of almost any carbohydrate-rich crop, including brandies (cognac and fruit brandies), vodka (originally from distilled beer), aquavit or schnapps (based on fermented potato), whisky (from fermented roasted barley), rum (from sugar cane), gin (from a re-distilled grain mash and juniper), and tequilas (from agave cactus). In addition, some distillates exist as sugar-sweetened liquors; alcoholic spirits exist both unsweetened and as sweetened products often spiced with anise (ouzo, pastis, etc.), or with alcoholic extracts of fruit (fruit liquors) [254].

Although these kinds of alcoholic beverages are commonly used as alcohol control beverages in biomarker studies on wine and beer [179], and in clinical trials [175, 240, 246, 255] only few studies have aimed to identify candidate biomarkers of intake of distillates and spirits. Only two studies have investigated plausible intake biomarkers of aniseed spirit and peppermint liquor, respectively [256, 257].

Ouzo, raki, pastis, sambuca, and mistra are alcoholic beverages with a relatively high concentration of anethole [258]. Furthermore, anethole is also present in anise and fennel tea, as well as in some drugs (e.g., expectorants, antitussive, antispasmodic) and in perfumery, although their dosages are much lower than what results from moderate consumption of anise spiced spirits [256, 259]. Anethole has therefore been described as a characteristic marker for the consumption of aniseed spirits. This compound has serum pharmacokinetics being useful in verifications of post-offense drinking claims. Three hours after drinking 120 mL of ouzo and 7 h after consumption of 360 mL of ouzo, anethole levels in serum were still detectable [256]. As a note, this intake level would also allow detection of general alcohol intake biomarkers, and even for a more extended period.

Menthone occurs in four optically active stereoisomers, while menthol occurs in eight. Menthol is commonly used in toothpaste, mouthwash, and pharmaceutical preparations [260]. It has been detected also in tobacco products [261], Chinese medicinal herbs [262], and honey [263]. Menthone, isomenthone, neomenthol, and menthol have been proposed as peppermint liquor biomarkers [257]. The kinetic profiles of these compounds in the serum have been established after conducting three dose–response drinking experiments [257]. The concentration changes indicated rapid absorption, similar to the blood-alcohol concentration peak. Determination of menthone, isomenthone, neomenthol, and menthol within an approximate time frame of 30 min to 4 h in serum makes them very suitable biomarkers of recent intake of spirits containing these flavor materials such as peppermint liquor, mint liquor, and digestif bitters. However, as serum menthol and neomenthol levels may be also altered through the consumption or use of pharmaceutical and dental products, peppermint sweets, and teas, they cannot be regarded as specific individual markers. However, menthol and neomenthol may be specific in combination with alcohol intake biomarkers [257].

In a prospective, randomized, controlled cross-over trial with 61 subjects at high cardiovascular risk, comparison of markers of three different beverages (gin, red wine, and dealcoholized red wine) showed two significantly correlated (unidentified) urinary compounds following the consumption of gin. However, these unknown potential gin intake biomarkers were also present in some baseline samples, and present in all urinary metabolomes following intake of gin [238]. Others studied the effect of alcohol on urinary excretion of the disulfide, 2-thiothiazolidine-4-carboxylic acid (TTCA), among non-exposed subjects, and showed that high liquor intakes (150–250 mL) may interfere with the levels of urinary TTCA [264]. However, TTCA levels have also been proposed to reflect crucifer intake [265]. In some other studies, spirits, and distillates have been used with the aim to identify general markers for alcohol consumption (e.g., PEth, EtG, FAEE) and not for finding specific intake biomarkers of spirits or distillates [131, 266–270].

#### Marker validation

Among the candidate biomarkers of distillates and spirits, anethole has been investigated for aniseed spirits, while menthone, isomenthone, neomenthol, and menthol have been proposed as peppermint liquor intake biomarkers. Anethole seems to be a promising biomarker for this type of distillate but still needs to be validated by independent verification and by measurement in controlled cross-sectional studies to confirm its reproducibility. The potential application of menthol is currently still hampered by the lack of robustness due to the common use of this flavoring. Combining menthol with an ethanol biomarker might decrease the level of misclassification, while more research is also needed in terms of analytical performance, robustness, and reproducibility. The full validation criteria can be found in Table 1.

## Discussion

In this extensive literature review, we have used the BFIRev guidelines to cover all reports on biomarkers related to moderate alcohol intake and use of alcoholic beverages. The search resulted in more than 20,000 titles of which ~ 170 papers reported directly on biomarkers and applications in human studies. These markers include five main direct markers of alcohol intake, ethanol, EtG, EtS, FAEEs, and PEth; two main markers of wine intake, RV metabolites, and tartrate; three main groups of markers of beer intake, xanthohumol metabolites, IAA metabolites, and hordenine-related metabolites. Few of these are perfect markers, but in combinations also including some other compounds, they attain good or very good performance for assessing intakes. These results point at the necessary future work needed to identify the best biomarker combinations and to validate them according to guidelines.

Compared with other food group biomarkers, BFIs of alcoholic beverages are among the most extensively investigated and several markers of alcohol intake are in common legal use. Therefore, biomarkers for alcohol intake are a showcase for the development of BFIs in general; it illustrates the usefulness and promise of the area, as well as the caveats and limitations, and hence the need for further development of the theory and technology for this area and for biomarkers in general.

Ethanol itself is the most obvious biomarker and has been extensively used for decades. However, as ethanol is also relatively quickly eliminated from the body it has a narrow detection time window [21]. Other commonly known biomarkers for total alcohol intake include some liver enzymes, MCV, CDT, and others, which have been mainly used for testing alcohol abuse and cannot be used at moderate intake levels. Some of the best markers of alcohol intake listed here can distinguish intake levels above and below moderation at the group level but have some caveats at the individual level related to analytical background, inter-individual variability in response, and kinetics; for instance, they do not yet allow to distinguish between recent intakes, chronic intakes, and the timing since last intake. Despite the fast elimination from the body, breath ethanol remains an important marker of recent intake, especially in relation to traffic offenses. Breath ethanol is a good reflection of blood alcohol levels and of the impact of alcohol on cognitive judgment and control of motor function, but longer-term markers are needed to reveal high intakes of alcohol within the last day or two. Potentially promising makers here include EtG, EtS, PEth, and FAEE. Plasma or urine levels of acetaldehyde could also potentially be developed to serve this purpose [18, 31, 36].

EtG and EtS have considerably longer half-lives in plasma than ethanol by covering moderate alcohol intake ≥ 24 h [46, 57], but inter-individual variability may be high [58]. EtG can also be detected in hair and provide insight into longer-term average intakes, but again inter-individual variability may be high, and analysis may be disturbed by hair products and by the sampling method [67, 68]. Although the application of EtG in hair is still not fully validated at low to moderate intake levels, hair EtG analyses are already common in legal use. Hair FAEE is a promising marker used to verify hair EtG but also suffers from high analytical background levels and variable individual responses at similar intake levels. Plasma FAEE and blood or erythrocyte PEth are useful and reliable markers of alcohol abuse [86]. FAEE seems also promising as a marker of chronic alcohol abuse with apparently increased half-life after chronic high intakes, thereby potentially discriminating occasional binge drinkers from chronic abusers [60, 110].

In terms of the central theme of this review—i.e., identification of biomarkers to quantify moderate or low alcohol intakes for the purpose of dietary and nutrition studies—the four primary alcohol markers are useful, but their validation at this intake level is still not complete. The definition of what constitutes moderate alcohol intake has been changing over time with previous upper bounds of 40–60 g/day that are nowadays more commonly set at 10–30 g/day [3, 4]. At the group level, these intake ranges are relatively well studied for EtG, EtS, FAEE, and PEth, and all of them can discriminate between low, moderate, and high intakes. At the individual level, there is often some overlap between the ranges observed for each of these three intake levels, most likely due to inter-individual differences in the activities and kinetics of the enzymes involved in ethanol metabolism, and inter-individual differences in biomarker degradation and excretion. Further investigation of these factors is needed to fully validate the markers for detecting and quantifying individual low intakes.

Studies combining information from two or more of these markers indicate that improved classification of individuals’ recent intakes can be achieved [114]. None of the individual markers is able to discriminate non-consumers from subjects with sporadic or daily low alcohol intakes. Available studies indicate EtS and EtG are currently the most promising biomarkers for alcohol intake. However, it should also be noted that the fractional formation of EtS and EtG is low at intake levels below one unit [25, 51, 57]. When the alcohol is not consumed within a short time span (10–30 min), the peak blood alcohol concentration will not exceed the Km for the involved enzymes, leading to an even lower response [57]. The high Km of formation, the variability in EtG and EtS formation between subjects due to polymorphisms, and the competition for the enzymes by other substrates are all factors making it difficult to measure accurately the individual low or null intakes [58]. The Km values for FAEE and PEth formation need to be investigated but are likely much lower. PEth shows considerable promise but needs further validation, and assessing the analytical background levels in human blood samples would improve our understanding of how to use this marker at low intakes [58]. Even non-consumers (e.g., children) sometimes have non-zero levels of all alcohol biomarkers when sensitive analytical methods are used, and the cause of this apparent background exposure has not been studied in much detail [25]. Apart from non-compliance, which may not explain all the documented cases, some other factors could affect the biomarkers. One of these is the “hidden” alcohol in many common foods, e.g., in many fermented foods (bread, dairy), in fruit and fruit juices, and others; the exposure levels from these sources are low but potentially variable and high intakes of some of these foods may cause non-zero biomarker levels with sensitive analyses. Another explanation is the potential endogenous alcohol formation from human or microbial metabolism; the latter is well known to cause incidences of the auto-brewing syndrome where non-drinking victims have biomarker levels usually associated with alcohol abuse, i.e., alcohol is formed faster than it is degraded, leading to a build-up of intoxication [24]. It is unknown whether much lower levels of auto-brewing may be a common phenomenon, explaining non-zero background levels of the biomarkers. Due to the relatively high Km of the alcohol intake biomarkers, EtG and EtS, they will only be formed at trivial levels if auto-brewing would take place at half a unit an hour or less, and the ethanol production would therefore go undetected, unless measured as ethanol itself by GC–MS; FAEE and PEth may be formed with lower enzymatic Km values and for these markers, low-level auto-brewing might result in background levels that would vary considerably between subjects, as also observed for most sample types [88, 99, 113]. Other factors such as polymorphisms, environmental factors, or misreporting might also play a role [92, 95]. Endogenous background formation of alcohol would be expected to increase biomarker levels in subject groups at increased risk of gut microbial dysbiosis, e.g., subjects suffering from small bowel microbial overgrowth, diabetes, or obesity. However, more direct investigations and evidence are needed to verify whether auto-brewing plays a role in forming a background exposure to alcohol, which may also add a new perspective to the commonly observed J-shaped association between alcohol intake and risk of cardiovascular disease or diabetes [271].

Additional evidence for low or moderate intake levels may come from biomarkers specific to the beverages commonly consumed, especially from beer or wine. There are several markers for beer and wine intake related to typical constituents, e.g., IX [174] or iso-α -acid metabolites [156, 157] from wort, N-methylated tyramine metabolites from barley sprouts [181], or RV metabolites [211, 217, 222] and tartrate [241] from grapes. In addition, the yeast fermentation used to brew these beverages leads to several metabolites, including hydroxyvalerates [124] and ethyl malate [156]. These and other markers are sufficiently well validated to identify intakes with good confidence and may therefore support the alcohol markers. However, most of these markers are only useful within 24 h of intake, while no markers exist to quantify longer-term intakes. Therefore, considerable work is still needed to develop and validate combined BFIs for each exposure scenario as well as developing of additional sample types or sampling techniques to provide reliable biomarkers for shorter- and longer-term low or moderate intakes of alcoholic beverages. Since beer and wine exist in very many forms and as non-alcoholic beverages, the known biomarkers will need additional validation and development to discriminate exposures in more detail.

In a recent study, the α-glucoside of ethanol was identified in Japanese sake, wine, and beer and in human blood and urine from seven autopsy cases [273]. In red and white wine mainly the β-isomer was observed while beer had equal levels of both isomers. Sake differed from wine and beer in having only the α-isomer of ethyl glucoside. The origin of the autopsy cases was not revealed but based on very high levels of the α- (26–837 µg/mL in urine and 1.4–33 µg/mL in blood) compared with the β-isomer (0–3.2 µg/mL in urine and 0–3 µg/mL in the blood) it might be assumed they were heavy users of sake. Commercial drug-free reference samples of blood and urine were measured as reference (levels < 0.3 µg/mL in the blood and < 0.6 µug/mL in urine); however, the actual origin of the compounds in the autopsy samples and whether the compounds might exist in other fermented foods than alcoholic beverages are still unknown. However, the two ethyl glucoside isomers show potential promise as future markers to discriminate sake drinkers, wine drinkers, and beer drinkers.

The current review has used the BFIRev [20] approach in analogy with multiple previous reviews for various food groups. By this approach, some papers may have been missed or misinterpreted since only one author has been selecting the papers relevant for each biomarker. Moreover, our method of evaluation is less stringent. Specifically, the overall classification by the validation criteria is based on a judgment on whether there is any evidence that the criterion may be fulfilled under certain conditions. In the case of alcohol biomarkers, where the majority of papers are concerned with the identification of problem drinkers, this tends to make the overall evaluation of most markers more favorable while their use for estimating lower intakes may be compromised. In forensics, the interest in abstinence has increased in recent years, especially as the adverse effects of alcohol intake during pregnancy have become clearer and legislation is emerging in some countries incriminating such alcohol use in order to protect the child. Consequently, the detection of abstinence is becoming more important, and potential sources of error in estimates of null and minimal intakes have become more urgent to identify. In the current review, the critical assessment of low intakes by the available BFIs for alcohol is the major point of focus and the caveats identified here may therefore spur new research to settle uncertainties, thereby also improving the legal assessment of cases where only abstinence is accepted.

In nutrition research alcohol intake is usually ignored, i.e., the contribution of alcohol to energy intake and to nutrition-related health is rarely included in experimental studies on dietary effects. Despite the high energy density of alcohol, current evidence indicates that moderate alcohol intake does not contribute to weight gain [272], but measurements of energy intake may still be offset. In the Mediterranean diet, low-dose wine intake is included and even recommended in dietary pattern interventions [274]. The trust in dietary assessment instruments when it comes to alcohol intake is debated and it is sometimes assumed that a large proportion of subjects’ misreport, especially those who report abstinence or low drinking [10]. Reliable biomarkers to discriminate between abstinence and low or moderate intakes, both long- and short-term markers, could therefore have a considerable impact on future nutrition and health research.

## Conclusion

Biomarkers covering the intake of alcoholic beverages rank among the most well-investigated and validated biomarkers of food and beverage intake. Biomarkers of alcohol, beer, and wine intake cover recent high or moderate intakes reasonably well, while low intakes may go unnoticed. Inter-individual variation, variability in drinking patterns, and variability in the beverage production processes all contribute as factors causing quantitative uncertainty regarding intakes while qualitative methods to discriminate no intake from moderate or high intakes are generally more reliable. Classification of no intake vs. low intakes is still only fair at best, which is unfortunate since the major controversy in research on moderate alcohol intake and health is the effects of abstention vs. low intakes. Several developments in biomarkers for alcoholic beverages and their non-alcoholic counterparts are therefore still needed, especially markers sensitive to low alcohol intakes, smart biomarker combinations to discriminate different recent or longer-term intake scenarios and potentially better sampling methods to cover intermittent intakes.

## Supplementary Information

**Additional file 1:** **Supplementary Table S1.** Keyword for the primary literature research. **Supplementary Table S2.** List of studies reporting candidate biomarkers for alcoholic beverage subgroups and ethanol consumption. **Supplementary Table S3.** Summary of the excluded candidate BFIs of alcoholic beverages subgroups and ethanol consumption and reasons for exclusion.
