# Correction: Biomarkers of moderate alcohol intake and alcoholic beverages: a systematic literature review

**DOI:** 10.1186/s12263-023-00728-z

**Published:** 2023-05-15

**Authors:** Marta Trius-Soler, Giulia Praticò, Gözde Gürdeniz, Mar Garcia-Aloy, Raffaella Canali, Fausta Natella, Elske M. Brouwer-Brolsma, Cristina Andrés-Lacueva, Lars Ove Dragsted

**Affiliations:** 1 Department of Nutrition, Exercise and Sports, Faculty of Science, University of Copenhagen, 1958 Frederiksberg C, Denmark; 2 Polyphenol Research Laboratory, Department of Nutrition, Food Sciences and Gastronomy, XIA School of Pharmacy and Food Sciences, University of Barcelona, 08028 Barcelona, Spain; 3 INSA-UB, Nutrition and Food Safety Research Institute, University of Barcelona, 08921 Santa Coloma de Gramanet, Spain; 4 Centro de Investigación Biomédica en Red de Fisiopatología de La Obesidad Y Nutrición (CIBEROBN), Instituto de Salud Carlos III, 28029 Madrid, Spain; 5 Biomarker & Nutrimetabolomics Laboratory, Department of Nutrition, Food Sciences and Gastronomy, Faculty of Pharmacy and Food Sciences, University of Barcelona, 08028 Barcelona, Spain; 6 Metabolomics Unit, Research and Innovation Centre, Fondazione Edmund Mach, San Michele All’Adige, Italy; 7 Consiglio Per La Ricerca in Agricoltura E L’analisi Dell’economia Agraria (CREA) Research Centre for Food and Nutrition, Rome, Italy; 8 Division of Human Nutrition and Health, Department Agrotechnology and Food Sciences, Wageningen University and Research, P.O. Box 17, 6700 AA Wageningen, The Netherlands; 9 Centro de Investigación Biomédica en Red de Fragilidad Y Envejecimiento Saludable (CIBERFES), Instituto de Salud Carlos III, 28029 Madrid, Spain

**Correction: Genes & Nutrition 18, 7 (2023)**

**https://doi.org/10.1186/s12263-023-00726-1**

Following publication of the original article [1], it was flagged that the author Fausta Natella's name had been inverted (i.e., as Natella Fausta) in the author list. The article has since been updated and the corrected name may be seen in the author list of this erratum.
