# Supplementary material for: Biomarkers of moderate alcohol intake and alcoholic beverages: a systematic literature review
Source: Genes Nutr. 2023 Apr 19;18:7. doi: 10.1186/s12263-023-00726-1 (PMC10114415; doi:10.1186/s12263-023-00726-1)
Supplement: Supplementary file 1 — Additional file 1: Supplementary Table S1. Keyword for the primary literature research. Supplementary Table S2. List of studies reporting candidate biomarkers for alcoholic beverage subgroups and ethanol consumption. Supplementary Table S3. Summary of the excluded candidate BFIs of alcoholic beverages subgroups and ethanol consumption and reasons for exclusion. [file 12263_2023_726_MOESM1_ESM.docx]

**Biomarkers of moderate alcohol intake and alcoholic beverages: a systematic literature review**

Marta Trius-Soler^1-4^, Giulia Praticò^1^, Gözde Gürdeniz^1^, Mar Garcia-Aloy^5,6^, Raffaella Canali^7^, Natella Fausta^7^, Elske M. Brouwer-Brolsma^8^, Cristina Andrés-Lacueva^3,5,9^, Lars Ove Dragsted^1^

^1^Department of Nutrition, Exercise and Sports, Faculty of Science, University of Copenhagen, 1958 Frederiksberg C, Denmark

^2^ Polyphenol Research Laboratory, Department of Nutrition, Food Sciences and Gastronomy, XIA School of Pharmacy and Food Sciences, University of Barcelona, 08028 Barcelona, Spain; ﻿

^3^INSA-UB, Nutrition and Food Safety Research Institute, University of Barcelona, 08921 Santa Coloma de Gramanet, Spain.

^4^Centro de Investigación Biomédica en Red de Fisiopatología de la Obesidad y Nutrición (CIBEROBN), Instituto de Salud Carlos III, Madrid, 28029, Spain.

^5^Biomarker & Nutrimetabolomics Laboratory, Department of Nutrition, Food Sciences and Gastronomy, Faculty of Pharmacy and Food Sciences, University of Barcelona, 08028 Barcelona, Spain.

^6^Metabolomics Unit, Research and Innovation Centre, Fondazione Edmund Mach, San Michele all'Adige, Italy.

^7^Research Centre for Food and Nutrition, Consiglio per la ricerca in agricoltura e l’analisi dell’economia agraria (CREA), Rome, Italy

^8^Division of Human Nutrition and Health, Department Agrotechnology and Food Sciences, Wageningen University and Research, P.O. Box 17, 6700 AA Wageningen, The Netherlands

^9^Centro de Investigación Biomédica en Red de Fragilidad y Envejecimiento Saludable (CIBERFES), Instituto de Salud Carlos III, Madrid, 28029, Spain

* Corresponding author: E-mail address: ldra@nexs.ku.dk; Tel: (+45)35332

**Supplementary Table 1.** Keyword for the primary literature research

| **Operator** | **Database** | **Field** | **Keywords** |
| --- | --- | --- | --- |
| **Common keywords** | | | |
| **AND** | ***Pubmed*** | *All Fields* | biomarker OR marker OR metabolite OR biokinetics OR biotransformation |
|  | ***Web of Science*** | *Topic* | biomarker* OR marker* OR metabolite* OR biokinetics OR biotransformation |
|  | ***Scopus*** | *Article Title/ Abstract/ Keywords* |  |
| **AND** | ***Pubmed*** | *All Fields* | trial OR experiment OR study OR intervention |
|  | ***Web of Science*** | *Topic* |  |
|  | ***Scopus*** | *Article Title/ Abstract/ Keywords* |  |
| **AND** | ***Pubmed*** | *All Fields* | human OR men OR women OR patient OR volunteer OR participant |
|  | ***Web of Science*** | *Topic* | human* OR men OR women OR patient* OR volunteer* OR participant* |
|  | ***Scopus*** | *Article Title/ Abstract/ Keywords* |  |
| **AND** | ***Pubmed*** | *All Fields* | urine OR plasma OR serum OR blood OR excretion |
|  | ***Web of Science*** | *Topic* |  |
|  | ***Scopus*** | *Article Title/ Abstract/ Keywords* |  |
| **AND** | ***Pubmed*** | *All Fields* | intake OR meal OR diet OR ingestion OR consumption OR drink* OR administration |
|  | ***Web of Science*** | *Topic* |  |
|  | ***Scopus*** | *Article Title/ Abstract/ Keywords* |  |
| **Specific keywords for “alcohol”** | | | |
| **AND** | ***Pubmed*** | *Title/Abstract* | alcohol OR ethanol |
|  | ***Web of Science*** | *Topic* |  |
|  | ***Scopus*** | *Article Title/ Abstract/ Keywords* |  |
| **Specific keywords for “beer”** | | | |
| **AND** | ***Pubmed*** | *Title/Abstract* | beer OR lager |
|  | ***Web of Science*** | *Topic* |  |
|  | ***Scopus*** | *Article Title/ Abstract/ Keywords* |  |
| **NOT** | ***Pubmed*** | *All Fields* | “Beer’s Law” |
|  | ***Web of Science*** | *Topic* |  |
|  | ***Scopus*** | *Article Title/ Abstract/ Keywords* |  |
| **Specific keywords for “cider”** | | | |
| **AND** | ***Pubmed*** | *Title/Abstract* | cider OR "apple wine" OR "fermented juice apple" OR "fermented apple*” |
|  | ***Scopus*** | *Article Title/ Abstract/ Keywords* |  |
| **Specific keywords for “wine”** | | | |
| **AND** | ***Pubmed*** | *Title/Abstract* | wine |
|  | ***Web of Science*** | *Topic* |  |
|  | ***Scopus*** | *Article Title/ Abstract/ Keywords* |  |
| **Specific keywords for “sweet wine”** | | | |
| **AND** | ***Pubmed*** | *Title/Abstract All Fields* | “sweet wine” OR "dessert wine" OR "sparkling wine" |
|  | ***Scopus*** | *Article Title/ Abstract/ Keywords* |  |
| **Specific keywords for “distillates and spirits”** | | | |
| **AND** | ***Pubmed*** | *Title/Abstract* | distillate OR distilled beverage OR spirit OR liquor OR liqueur OR whiskey OR whisky OR wodka OR rum OR brandy OR cognac OR tequila OR gin OR eggnog OR schnapps OR vodka |
|  | ***Web of Science*** | *Topic* |  |

**Supplementary Table 2.** List of studies reporting candidate biomarkers for alcoholic beverage subgroups and ethanol consumption

| Dietary factor | Study design^1^ | Study population | Analytical method | Sample type | Discriminating metabolites / Candidate biomarkers | Refs. |
| --- | --- | --- | --- | --- | --- | --- |
| **Alcohol in general** |  |  |  |  |  |  |
| All alcoholic beverages | Cross-sectional study | 6705 subjects (66% males)  (median age 54.5 years) | UPC2-MS/MS  Chemical method | Serum  Serum | PEth 16.0/18.1  Ethanol | [1] |
| All alcoholic beverages | Cross-sectional (baseline in a longitudinal cohort study) | 5676 Dutch subjects (51.2% males, 53 ± 12 years) | Colorimetric method (DRI dipstick) | 24h Urine | EtG | [2] |
| All alcoholic beverages | Cross-sectional study | 4067 pregnant women; 466 were additionally tested later (18-50 years) | UPLC-MS/MS | Blood | PEth 16:0/18:1 | [3] |
| All alcoholic beverages | Cross-sectional study | 1875 traffic offenders (87% males)  (mean age 40 years, 14-81 years) | Breath analyzer  HS-GC-FID | Breath  Blood | Ethanol  Ethanol | [4] |
| All alcoholic beverages | Cross-sectional study | 1872 subjects (abstinent, low-moderate, moderate, and excessive alcohol consumption) (age n.p.) | GC-MS  LC-ESI-MS | Hair  Hair | Total FAEEs  EtG  Combined biomarker: FAEE, EtG | [5] |
| All alcoholic beverages | Cross-sectional study | 849 subjects (57% men, 62.2 ± 16.5 years) | LC-MS | Serum | EtG | [6] |
| All alcoholic beverages | Cross-sectional study | 1369 postmenopausal women (68.3 ± 5.7 years) | LC-MS | Serum | EtG | [7] |
| All alcoholic beverages | Cross-sectional study | 533 females and 506 males at increased CVD risk (55-80 years) | Colorimetric method | Morning spot urine | EtG | [8] |
| All alcoholic beverages | Cross-sectional study | 340 males and 304 females (abstinent, moderate, excessive alcohol consumption or unknown) (age n.p.) | GC-MS | Hair | Ethyl myristate, ethyl palmitate, ethyl oleate, ethyl stearate, total FAEEs | [9] |
| All alcoholic beverages | Cross-sectional study | 305 pregnant women in their first trimester coming for first examination, 188 abstainers and 117 with pre-conception alcohol intakes (mean age around 32 years) | HPLC-MS/MS | Blood | Sum of PEth 16:0/16:0, 16:0/18.1 and 18:1/18:1 | [10] |
| All alcoholic beverages | Cross-sectional study | 373 females and 231 males (18-26 years) | LC-MS/MS  LC-MS/MS | Fingernail  Hair | EtG  EtG | [11] |
| All alcoholic beverages | Cross-sectional study | 221females and 281males (65 ± 5 years) | UHPLC-MS or  GC-MS | Serum | EtG | [12] |
| All alcoholic beverages | Cross-sectional study | 509 subjects referred for evaluation of risky drinking from employment agencies (80% males) (age n.p.) | UPLC-MS/MS  UPLC-MS/MS  Colorimetric method | Blood  Serum  Serum | PEth 16:0/18:1  EtG  Ethanol | [13] |
| All alcoholic beverages | Cross-sectional study | 418 women (163 pregnant) all with alcohol intake interviews (median (IQR) age 29, 24-35 years) | LC-MS/MS | Dried blood spots | PEth (unspecified) | [14] |
| All alcoholic beverages | Cross-sectional study | 370 legal cases (sex and age n.p.) | Breath analyzer  HS-GC-FID | Breath  Blood | Ethanol  Ethanol | [15] |
| All alcoholic beverages | Cross-sectional study | 300 subjects (abstinence, moderate and excessive alcohol consumption) (50 % males) (mean age around 45 years) | LC-MS/MS | Blood | PEth 16:0/18:1, PEth 16:0/18:2 | [16] |
| All alcoholic beverages | Cross-sectional study | 97 hemorrhagic stroke cases and 180 matched referents (39% females, mean age 55 years, 25-74 years) | LC-MS/MS | Blood | PEth 16:0/18:1 | [17] |
| All alcoholic beverages | Cross-sectional study | 100 female sex workers and 100 male clients (median age 24.5, 21-29 years) | LC-MS/MS | Dried blood spots | PEth 16:0/18:1 | [18] |
| All alcoholic beverages | Cross-sectional study | 139 males, 29 females and 6 with no sex data  (0-70 years, 10 with no age data) | HS-SPME-GC-MS  LC-ESI-MS-MS  n.p. | Hair  Hair  Blood | Total FAEEs  EtG  Combined biomarker: Total FAEEs, EtG  Ethanol | [19] |
| Study 1: All alcoholic beverages  Study 2: All alcoholic beverages | Cross-sectional study  Cross-sectional study | 100 former abusers (age and sex n.p.)  12 males (8 based on suspected drinking, 6 from drinking denied, and 4 from high-risk individuals) (36-58 years) | HPLC-MS/MS  HPLC-MS/MS | Urine  Urine | EtG, ethanol  EtG, ethanol | [20] |
| All alcoholic beverages | Cross-sectional study | 92 emergency room patients, 15 chronic alcoholics and 15 episodic heavy drinkers from clinical trials (age n.p.) | HPLC  GC | Serum  Blood | Ethyl oleate, Ethyl stearate, total FAEEs  Ethanol | [21] |
| All alcoholic beverages | Cross-sectional study | 97 subjects providing hair to resolve insurance cases (sex and age n.p.) | LC-MS/MS  HS-SPME-GC-MS | Hair  Hair | EtG  Total FAEEs | [22] |
| All alcoholic beverages | Cross-sectional study | 50 abstainer subjects and 14 in withdrawal treatment (sex and age n.p.) | LC-MS/MS | Hair | EtG | [23] |
| All alcoholic beverages | Cross-sectional study | 17 teetotalers, 20 moderate social drinkers, 47 patients in withdrawal treatment and 171 death cases (sex and age n.p.) | GC-EI-MS  GC-MS  LC-MS-MS | Hair  Hair  Hair | Ethyl myristate, ethyl palmitate, ethyl oleate, ethyl stearate, total FAEEs  EtG  EtG  Combined biomarker: Total FAEEs, EtG | [24] |
| All alcoholic beverages | Cross-sectional study | 2 abstainers, 20 moderate alcohol consumers, 36 excessive alcohol consumers (sex and age n.p.) | GC-MS  GC-MS/MS | Hair  Hair | EtG  EtG | [25] |
| All alcoholic beverages | Cross-sectional study | 30 healthy males and 15 females (22-66 years) with regular drinking habits | LC-MS/MS | Blood | PEth 16:0/18:1, PEth 16:0/18:2, PEth 16:0/16:0, PEth 18:1/18:1, total PEth | [26] |
| All alcoholic beverages | Cross-sectional study | 20 men and 24 women abstainer or teetotaler (1-80 years) | HPLC-MS/MS | Hair | EtG | [27] |
| All alcoholic beverages | Cross-sectional study | 29 alcoholics (10 autopsies, 19 in withdrawal treatment, 13 moderate social drinkers and 5 teetotalers) (sex and age n.p.) | HS-SPME-GC-MS | Hair | Ethyl myristate, ethyl palmitate, ethyl oleate, ethyl stearate, total FAEEs | [28] |
| All alcoholic beverages | Cross-sectional study | 10 alcoholics in withdrawal treatment, 11 death cases, 4 moderate social drinkers 3 teetotalers (sex and age n.p.) | HS-SPME-GC MS  GC-MS | Hair  Hair | Total FAEEs  EtG | [29] |
| All alcoholic beverages | Cross-sectional study | 13 teetotalers (5 males, 8 females, 6-48 years), 16 social drinkers (7 males, 9 females, 21-77 years) and 10 death cases with known recent alcohol miscues (9 males, 1 female, 35-60 years) and 5 deaths cases without indications of alcohol misuse (5 males, 37-63 years) | HS-SPME-GC-MS | Sebum (skin surface lipids) | Total FAEEs | [30] |
| All alcoholic beverages | Cross-sectional study | 22 alcoholic fatalities, 5 moderate social drinkers, and one teetotaler (26 males, 2 females, 25-65 years) | HS-SPME-GC-MS | Hair | Ethyl myristate, ethyl palmitate, ethyl oleate, ethyl stearate, total FAEEs | [31] |
| All alcoholic beverages | Cross-sectional study | 10 non-drinkers and 10 heavy drinkers (> 80 g/d) | GC-MS/MS | Blood plasma proteins | N^ε^-Ethyl-lysine (acetaldehyde adduct with lysine) | [32] |
| All alcoholic beverages | Cross-sectional study | 18 subjects (sex and age n.p.) | GC-MS  Colorimetric method | Serum  Serum | Ethyl palmitate, ethyl stearate, ethyl oleate, total FAEEs  Ethanol | [33] |
| All alcoholic beverages | Cross-sectional study | 8 heavy drinkers (1 female and 6 males; 44-55 years), 5 social drinkers (1 female and 4 males; 20-59 years); and 7 teetotalers (4 females and 3 males; 26-50 years) | HPLC-MS/MS | Blood | PEth 16:0/18:1, PEth 16:0/18:2, PEth 16:0/16:0, PEth 18:1/18:1 | [34] |
| All alcoholic beverages | Cross-sectional study | 6 ethanol-negative blood samples; 6 ethanol-spiked samples, 6 ethanol-positive blood samples | HS-GC  UHPLC-MS/MS | Postmortem blood samples  Postmortem blood samples | Ethanol  EtG, EtS, total FAEEs, total PEth | [35] |
| All alcoholic beverages | Cross-sectional study | 3 moderate drinkers, 1 teetotaler | CE-MS/MS | Hemoglobin chains | Acetaldehyde | [36] |
| All alcoholic beverages | Cross-sectional study | n.p. | GC-MS  GC-MS | Serum lipoprotein fractions (HDL, LDL, VLDL)  Blood | Ethyl oleate, total FAEEs  Ethanol | [37] |
| All alcoholic beverages | Cross-sectional study | 213 liver transplant patients (71% previously alcohol dependent (61.3% males, 59.02 ± 10.33 years) and 29% not dependent (72.2% males, 59.26 ± 8.09 years) | LC-MS/MS | Dried blood spots | PEth 16.0/18:1 | [38] |
| All alcoholic beverages | Case-control study | 85 pregnant women with substance abuse (42 with self-reported prenatal alcohol exposure and 43 controls) (26.7 ± 4.8 years) | LC-MS/MS  LC-MS/MS | Hair  Urine | EtG  EtG, EtS, PEth (unspecified)  Combined biomarkers: hair EtG + urine EtG, hair EtG + urine EtS, hair EtG + PEth | [39] |
| All alcoholic beverages | Case-control study | 8 men and 4 women with acute intoxication (43 ± 3 years) and 15 abstaining men (24 ± 1 years) | GC-MS  GC-MS | Pre and postprandial 0-44h serum  Pre and postprandial 0-44h lipoprotein fractions (VLDL, LDL, HDL, HDL infranatant) | Ethanol, total FAEEs  Total FAEEs | [40] |
| Study 1: All alcoholic beverages  Study 2: All alcoholic beverages | Case study  Cross-sectional study | 1 positive hair pool sample (sex and age n.p.)  29 children (1-12 years) (sex n.p.) | n.p.  n.p. | Hair  Hair | Ethyl palmitate, ethyl stearate, ethyl oleate, total FAEEs  Ethyl palmitate | [41] |
| All alcoholic beverages | Cross-sectional study | 15 students (sex not n.p.)  Providing information on average intakes | LC-MS/MS  GC-MS/MS | Hair  Hair | EtG  FAEEs | [42] |
| All alcoholic beverages | Longitudinal cohort study with a cross-sectional comparator group | 9 male and 3 female withdrawal inpatients (30-58 years) with frequent blood collections over 8-33d and 38 male and 38 female healthy social drinkers (18-75 years) | LC-MS/MS | Blood | PEth 16:0/18:1, PEth 16:0/18:2, PEth 18:0/18:2, PEth 18:0/18:1, PEth 18:1/18:1, PEth 18:0/20:4, PEth 16:0/20:3, PEth 16:0/16:0, PEth 18:0/20:3, PEth 16:1/18:2, PEth 18:1/18:2, PEth 17:0/18:1, PEth 16:0/22:4, PEth 16:0/18:0, total PEth | [43] |
| Study 1: Ethanol abstention  Study 2: Ethanol (as dermal exposure from 61% (w/w) ethanol hand-sanitizer)  Study 3: Ethanol (3, 6, 12, 24 g of alcohol as vodka in water or juice) | Cross-sectional study  5d intervention repeated exposure  4 x intervention cross-over dose and time-response single exposure | 10 girls and 3 boys (<10 years) and 39 adult abstainers  6 abstainers males and 3 abstainers females  4 males social drinkers (age n.p.) | LC-MS/MS  LC-MS/MS  Colorimetric method  LC-MS/MS  Colorimetric method | Urine (first morning void in adults, children spot samples at unknown times)  Pre and postprandial 0-2 to 5d urine and an additional 7d of morning urine  Pre and postprandial 0-75h urine | EtG  EtG  Ethanol  EtG  Ethanol | [44] |
| All alcoholic beverages | 5-20w (median of 12 w) longitudinal cohort study | 25 men (25-83 years) and 11 women (32-66 years) | HPLC-MS | Blood (1 to 4 times between 5–20d time span) | PEth 16:0/18:1 | [45] |
| All alcoholic beverages | 2w longitudinal cohort study | 37 men (49.5 (23-72) years) and 12 women (48.7 (30-66) years) heavy drinkers within a detoxification process | LC-MS/MS  LC-MS/MS  Breath analyzer | Blood (once daily over 5d and then every second day until 2w)  Urine (daily during 2w)  Breath | PEth 16.0/18.1, PEth 16.0/18.2, PEth 16.0/24.0  EtG, EtS  Ethanol | [46] |
| All alcoholic beverages | 33 ± 26d (3–74d))  longitudinal cohort study | 13 males and 6 females (47 ± 12 years) | LC-MS/MS  LC-MS/MS | Urine  Dried blood (venous DBS and capillary DBS) and whole blood | EtG, EtS  PEth 16.0/18.1, PEth 16.0/18.2 | [47] |
| Study 1: Ethanol (26-32 mM/L alcohol blood concentration as 1:3 ratio vodka with fruit juice)  Study 2: All alcoholic beverages | Intervention time-response after single exposure  Cross-sectional study | 4 males and 3 females (21-23 years)  48 samples from anonymous donors (sex and age n.p.) | GC-MS  GC  GC-MS  GC | Pre and postprandial 0-24h serum and plasma  Pre and postprandial 0-24h serum  Blood  Blood | Total FAEEs  Ethanol  Total FAEEs  Ethanol | [48] |
| Study 1: Ethanol (as white wine or beer)  Study 2: All alcoholic beverages | Intervention time-response after single exposure  Cross-sectional study | 5 males and 7 females (20-41 years)  10 males and 3 females heavy intoxicated by alcohol (30-55 years) | LC-MS/MS  Breath analyzer  LC-MS/MS  Breath analyzer | Pre and postprandial 0-28.5h urine  Postprandial breath  Postprandial 36-132h urine  Postprandial breath | EtG, EtS  Ethanol  EtG, EtS  Ethanol | [49] |
| All alcoholic beverages | 5d longitudinal cohort study after acute withdraw | 24 men and 6 women heavy drinkers with acute ethanol intoxication (43 ± 7 years) and 17 healthy subjects who took part in a drinking experiment (24 ± 2 years) (sex n.p.) | GC  GC-MS | Postprandial serum (0-5d twice daily between 8:00-9:00h and between 18:00-19:00h) | Ethanol  Total FAEE | [50] |
| Ethanol (4.5 to 6 oz of alcohol as beer, wine, or liquor) | Intervention time-response after single exposure | 42 healthy males (21-60 years) | GC-MS Colorimetric method | Postprandial 0.5–2 h blood Postprandial 0.5–2 h saliva | Ethanol  Ethanol | [51] |
| Ethanol (100 mL of sparkling wine) | Intervention time-response after single exposure | 18 males and 12 females with Gilbert’s syndrome (18-70 years) | LC-MS/MS  HS-GC-FID | Pre and postprandial 0-24h urine  Postprandial 3h blood | EtG, EtS  Ethanol | [52] |
| Ethanol (64 to 184 g of alcohol as beer, spirits, or wine) | Intervention time-response after single exposure | 17 healthy males (22-29 years) | HPLC  LC-MS/MS  GC  GC-MS | Postprandial urine (0-3d, twice daily between 8:00-9:00h and between 17:00-18:00h)  Postprandial serum (0-3d, twice daily between 8:00-9:00h and between 17:00-18:00h) | 5-HTOL/5-HIAA ratio  EtG  Ethanol  Sum of ethyl palmitate and ethyl stearate | [53] |
| Ethanol (0.5-0.8 g/kg of blood alcohol concentration as white wine) | Intervention time-response after single exposure | 6 healthy females and 7 males (19–42 years) | HPLC-MS/MS  HPLC-MS/MS  GC-FID and  Colorimetric method | Pre and postprandial 0-48h urine  Pre and postprandial 0-10h serum  Pre and postprandial 0-10h urine and serum | EtG, EtS EtG, EtS  Ethanol | [54] |
| Ethanol (3.6 mL of alcohol/L of water body mass as red wine) | 2 x intervention time-response after single exposure | 8 men and 4 women (38.6 years) | Breath analyzer | Pre and postprandial 0-4h breath | Ethanol | [55] |
| Study 1: All alcoholic beverages with orange juice (0.8 g of alcohol/kg of body weight)  Study 2: Ethanol (as white wine (0.8 g of alcohol/kg of body weight) or ethanol with orange juice (0.4 g of alcohol/kg of body weight)) | Intervention time-response after single exposure  Intervention time-response after single exposure | 5 women and 5 men (23-39 years)    3 women and 5 men (23-39 years) | GC-MS  Colorimetric method  HPLC  Colorimetric method  GC-MS  Colorimetric method  HPLC  Colorimetric method | Pre and postprandial 0-48h urine  Pre and postprandial 0-8h blood  Pre and postprandial 0-24h urine  Pre and postprandial 0-7h blood | 5-HTOL  Ethanol  5-HIAA  Ethanol  5-HTOL  Ethanol  5-HIAA Ethanol | [56] |
| Ethanol (1 mL of alcohol/kg of body weight as red wine and alcohol 40%) | Intervention time-response after single exposure | 8 subjects (21-67 years) | GC  GC  HPLC | Pre and postprandial 0-2h blood  Pre and postprandial 0-2h saliva  Pre and postprandial 0-2h blood | Ethanol, methanol  Methanol  Formaldehyde | [57] |
| Ethanol (0.8 g/kg blood alcohol concentration as vodka mixed with a soft drink) | Intervention time-response after single exposure | 6 men and 2 women (19-26 years) | LC-MS/MS  HS-GC-FID | Postprandial 100 min-5h dried blood spots  Postprandial 100 min-5h serum | Ethyl myristate, ethyl palmitate, ethyl oleate, ethyl stearate, total FAEEs  Ethanol | [58] |
| Ethanol (9-18 g of alcohol as sparkling wine) | Intervention time-response after single exposure | 4 males and 4 females (21-63 years) | LC-MS/MS | Pre and postprandial 0-44h urine | EtG, EtS | [59] |
| Ethanol (0.08% blood alcohol concentration as vodka mixed with a soft drink) | Intervention time-response after single exposure | 2 females and 6 males (age n.p.) | LC-MS/MS | Pre and postprandial 0-6h blood | PEth 16.0/18.1, PEth 16.0/18.2 | [60] |
| Ethanol (25 g of alcohol as white wine) | Intervention time-response after single exposure | 4 healthy females and 3 males (37 ± 5 years) | LC-MS/MS  Colorimetric method | Pre and postprandial 0-9h urine  Pre and postprandial 0-9h urine | EtG  Ethanol | [61] |
| Study 1: Ethanol (47 g ethanol for males or 32 g ethanol for women (as vodka) and diluted by diet-Coke® up to 500 mL)  Study 2: All alcoholic beverages | Intervention time-response after single exposure  Intervention dose and time-response after repeated exposure | 3 men , 2 women (25-47 years) abstaining for 5d prior to the single dose  8 men, 4 women (19-31 years) | HPLC-ELSD  Breath analyzer  HPLC-ELSD | Pre and postprandial 0-5d blood  Pre and postprandial breath on 0d  Pre and postprandial 0-2d blood | PEth (unspecified) (undetected), ethanol  Ethanol  PEth (unspecified) (detected), ethanol | [62] |
| Ethanol (47.52 g of alcohol as beer) | Intervention time-response after repeated exposure | 17 men and 7 women drinking (24-52 years) | HS-GC-MS  LC-MS/MS | Postprandial 30 min blood  Pre and postprandial 0-12.5h urine (at 7 time points) | Ethanol  EtG, EtS | [63] |
| Study 1: Ethanol with lemon (placebo, 18g, 30g)  Study 2: Ethanol with lemon (placebo, 6, 12 g)  Study 3: Ethanol with lemon (placebo, 24, 42 g) | 3x intervention cross-over, double-blind, dose and time-response after single exposure | 12 males (20-36 years)  6 males (20-36 years)  6 males (20-36 years) | Colorimetric method  GC-MS  LC-MS | Pre and postprandial 0-6h plasma  Pre and postprandial 0-6h plasma  Pre and postprandial 0-24h  urine | Ethanol  FAEEs  EtG | [64] |
| Study 1: Ethanol (0.5, 0.7, 0.7 g) and placebo *vs*. ethanol (0.5, 0.7, 0.7 g) and 4-methylpyrazole (10, 15, 20 g)  Study 1: Ethanol and 4-methylpyrazole *vs.* placebo and 4-methylpyrazole | 6 x intervention cross-over, double-blind, dose and time- response after single exposure  3 x intervention cross-over, double-blind, dose and time- response after single exposure | 4 males (age n.p.)  4 males (age n.p.) | GC  HPLC-DAD | Pre and postprandial 0-24h blood  Pre and postprandial 0-36h urine | Ethanol  Ethanol | [65] |
| Ethanol (1, 2 and 3 standard drinks as vodka) | 3 x intervention dose and time-response after single exposure | 10 women (21-39 years) | LC-MS  Breath analyzer | Pre and postprandial 0-72h urine  Pre and postprandial breath | EtG, EtS  Combined biomarker: EtG, EtS  Ethanol | [66] |
| Ethanol (12, 24, 28 g of alcohol) | 3 x intervention dose and time-response after single exposure | 12 female (22-29 years) | LC-MS/MS  Immunological method  LC-MS/MS  Dipstick | Pre and postprandial 0-48h serum  Pre and postprandial 0-48h serum  Pre and postprandial 0-72h urine  Pre and postprandial 0-72h urine | EtG  Ethanol  EtG  EtG | [67] |
| Ethanol (0.8 alcohol/kg of body weight) *vs.* banana *vs.* control | 3 x intervention partial cross-over, time-response after single exposure | 5-9 subjects (21-45 years) | GC-MS | Urine | 5-HTOL | [68] |
| Ethanol (0.5 g alcohol/kg body weight as beer, cachaça, red wine, or whiskey) | 4 x intervention cross-over, time- response after single exposure | 10 females and 10 males (29.5 ± 3 years) | Colorimetric method | Pre and postprandial 0-6h  plasma | Ethanol | [69] |
| Ethanol ((0.3 g alcohol/kg body weight as beer, white wine, dry sherry, or whiskey) | 4 x intervention parallel, time- response after single exposure | 11 healthy males (34 ± 3 years) | Breathalyzer | Pre and postprandial 0-4h  breath | Ethanol | [70] |
| Dealcoholized red wine (750 mL, 0.2% ethanol) *vs*. mouthwash *vs*. ethanol (1.8 g of alcohol) | 3 x intervention parallel, time- response after single exposure | 4 men and 8 women (29-30 years) | UPLC-MS/MS | Pre and postprandial 0-7.5h urine | EtS | [71] |
| Ethanol (pure, with tonic water)  (0.25 or 0.5 g ethanol/kg body weight) | 2 x intervention randomized, parallel, time- response after single exposure | 14 healthy men and 13 women one-week abstaining social drinkers (28.5 ± 8 years) | HPLC-MS/MS  Breath analyzer | Pre and postprandial 0-14d blood  Pre and postprandial 0-6d breath | PEth 16:0/18:1, PEth 16:0/18:2, combined PEth  Ethanol | [72] |
| Ethanol (pure, with tonic water)  (0.4 or 0.8 g ethanol/kg body weight) | 2 x intervention randomized, parallel, dose and time-response after single exposure | 27 healthy men and 27 women one-week abstaining social drinkers (27.6 ± 6.32 years) | HPLC-MS/MS  Breath analyzer  Transdermal ankle monitors | Pre and postprandial 0-14d blood  Pre and postprandial 0-6d breath  Pre and postprandial 0-22d ancle monitor | PEth 16:0/18:1, PEth 16:0/18:2, combined PEth  Ethanol  Continuous sweat alcohol monitor | [73] |
| Ethanol (0.5 g of alcohol/ kg of body weight as beer, vodka/tonic or white wine) | 3 x intervention cross-over, time- response after single exposure | 15 healthy men (25-65 years) | HS-GC | Pre and postprandial 0-8h blood | Ethanol | [74] |
| Alcohol (as red wine, 150/300 mL/d females/males) *vs.* control | 2 x 3m intervention randomized, parallel, open, controlled, response after repeated exposure | 44 (32 females and 12 males; 33.5 ± 9 years) | LC-MS/MS  LC-MS/MS | Blood  Hair (8-25mg) | PEth 16:0/18:1  EtG | [75]  [76] |
| Study 1: Ethanol (as vodka) (20, 80, 120 mg/dL blood alcohol)  Study 2: All alcoholic beverages (abstinence by mecamylamine treatment *vs*. placebo)  Study 3: All alcoholic beverages  (on moderation by naltrexone *vs.* placebo) | 4 x 3w intervention cross-over, dose and time-response after repeated exposure  2 x 12d intervention parallel response  2 x 12d intervention parallel response | 11 healthy males and 7 females; (21-60 years)  42 males and 5 females with problem drinkers (18-60 years)  63 males and 20 females with problem drinkers (18-25 years) | HPLC-MS/MS  Breath analyzer  HP-GS  HPLC-MS/MS  HPLC-MS/MS | Pre and postprandial 0-72h urine  Pre and postprandial breath  Pre and postprandial blood  Urine sampling at baseline and at 4w  Urine sampling at baseline and at 4w | EtG, EtS  Ethanol  Ethanol  EtG, EtS  EtG, EtS | [77] |
| Ethanol (0.03%, 0.05%, 0.07% alcohol blood concentration as vodka) | 3 x 3w intervention cross-over, dose and time-response after repeated exposure | 5 women and 5 men (22-30 years) | LC-MS/MS  Breath analyzer | Pre and postprandial 0-120h oral cavity cells and white blood cells  Postprandial breath (30 min) | N2-ethylidene-dG  Ethanol | [78] |
| Ethanol (0.8 g of alcohol/kg body weight as vodka) *vs.* placebo | 2 x 15d intervention randomized, cross-over, double-blind, controlled, time-response after repeated exposure | 27 ALDH wild type men and 27 heterozygous men (25 ± 3 years) | HS-GC-FID  LC-MS/MS | Pre and postprandial 0-6h blood  Pre and postprandial 0-6h blood | Ethanol  Acetaldehyde | [79] |
| Ethanol (40 g of alcohol as red wine and gin) *vs*. control (two different standard diets) | 4 x intervention cross-over, controlled response after single exposure | 8 men (45-55 years) | Colorimetric method | Postprandial 1h blood | Ethanol | [80] |
| **Beer** |  |  |  |  |  |  |
| Beer (mentioned in case circumstances) *vs.* positive BAC but beer was not mentioned *vs.* neither beer nor alcohol was mentioned | Cross-sectional study | 92 male ad 18 female cases with varying causes of death (mean or specific age range n.p.) | UHPLC-MS/MS | Blood (after a body admitted to the mortuary and at autopsy)  Serum, vitreous humor, and urine (at autopsy) | IAAs , reduced IAAs, ethanol  IAAs , reduced IAAs | [81] |
| Study 1: Beer (2 L)  Study 2: Different kinds of beer, wines, beer plus wine or beer plus digestive (0.4-1.50 g/kg of alcohol blood concentration) | Intervention time-response after single exposure | 1 subject (one of the authors) (sex and age n.p.)  10 subjects (sex and age n.p.) | UPLC–MS/MS  UPLC–MS/MS | Postprandial 0.5-6h serum  Serum | Hordenine, ethanol  Hordenine, ethanol | [82] |
| Beer (330 mL) | Intervention response after single exposure | 10 males (21-39 years) | UPLC-MS | Spot urine | Isoxanthohumol | [83] |
| Beer (762-1000 mL) | Intervention time-response after single exposure | 3 males and 1 female (33 ± 13 years) | UHPLC-MS/MS  GC-MS  GC-MS | Pre and postprandial 0-7.5h plasma Pre and postprandial 0-7.5h plasma  Pre and postprandial 0- 24h urine | Free hordenine, hordenine-Sulf, hordenine-Glc  Ethanol  Total hordenine | [84] |
| Beer (0.05% alcohol blood concentration as 2 lagers, 1 low lager) | 3 x intervention partially cross-over (1 subject consumed only one of the lagers), time-response after single exposure | 2 males and 3 females (25-39 years) | UHPLC–MS/MS  UHPLC–MS/MS | Pre and postprandial 0-6h blood    Pre and postprandial 0-6h urine | IAAs, reduced IAAs, ethanol  IAAs, reduced IAAs | [85] |
| Beer (0.05% alcohol blood concentration as 1 high-hopped beer, 1 low-hopped beer) | 2 x intervention time-response after single exposure | 5 males (25-44 years) | UPLC-MS/MS  UPLC-MS/MS | Pre and postprandial 0-6h blood  Pre and postprandial 0-6h urine | IAAs, ethanol  IAAs | [86] |
| Beer (2 L) *vs*. control | Intervention response after single exposure | 4 subjects (25-50 years) | SPE-LC-MS/MS | Postprandial 1-2h urine | Hordenine | [87] |
| Beer (0.05% alcohol blood concentration) | Intervention time-response after single exposure | 1 subject (sex and age n.p.) | UPLC-MS | Pre and postprandial 0-6h blood | IAAs and reduced IAAs | [88] |
| Study 1: Beer (300, 660 and 990 mL for males and 330, 495 and 660 mL for females)  Study 2: Beer (600 mL/day) *vs.* non-alcoholic beer (990 mL/day) *vs.* gin (92 mL/day)  Study 3: Beer | 3 x intervention cross-over, dose-response after single exposure  3 x 4w intervention randomized, cross-over, controlled, response after repeated exposure  Cross-sectional study | 20 males and 21 females (28 ± 3 years)  33 males (at high cardiovascular risk) (61 ± 7 years)  A subset of 32 males and 14 females (63 ± 5 years) | LC-MS/MS  UHPLC-MS  UHPLC-MS | Morning spot urine  24h urine  Morning spot urine | Isoxanthohumol  Isoxanthohumol  Isoxanthohumol | [89] |
| Beer (660 mL/day) *vs*. non-alcoholic beer (990 mL/day) *vs*. gin (100 mal/day) | 3 x 4w intervention randomized, crossover, open, response after repeated exposure | 33 males (55-75 years) | HPLC-LTQ-Orbitrap-MS | 24h urine | Humulinone, oxyhumulinic acid, cohumulone, EtS, EtG, 2-phenylethanol-GlcA | [90] |
| Beer (500 mL/day) *vs.* non-alcoholic beer (500 mL/day) | 2 x 14d intervention, cross-over, response after repeated exposure | 7 males (30-65 years) | UHPLC-MS  UHPLC-MS | Fasting plasma  Morning spot urine | Isoxanthohumol  Isoxanthohumol | [91] |
| Study 1: Beer (330 mL as strong lager, 1 regular lager 1 light/non-alcoholic beer) *vs.* soft drink (330 mL)  Study 2: Beer (660 mL as high-hopped beer *vs.* low-hopped beer) | 4 x intervention randomized, cross-over, single-blinded, time-response after single exposure  2 x intervention randomized, cross-over, single-blinded, time-response after single exposure | 10 males and 9 females (24-50 years)  2 males and 2 females (28-60 years) | UPLC-QTOF  UPLC-QTOF  UPLC-QTOF | Pre and postprandial 0-3h plasma  Pre and postprandial 0-21h urine and 24h pooled urine    Pre and postprandial 0-40h urine | Iso-cohumulone, pyro-glutamyl proline  Combined biomarker: N-methyl tyramine-Sulf, IAAs, tricyclohumols, pyro-glutamyl proline, 2-ethyl malate  Combined biomarker: N-methyl tyramine-Sulf, IAAs, tricyclohumols, pyro-glutamyl proline, 2-ethyl malate | [92] |
| **Cider** |  |  |  |  |  |  |
| Cider (500 mL) | Intervention time-response after single exposure | 9 healthy subjects (21-42 years) and 5 subjects with an ileostomy (40-54 years) | HPLC-PDA-MSn  HPLC-PDA-MSn  HPLC-PDA-MSn | Pre and postprandial 0-24h plasma  Pre and postprandial 0-24h urine  Pre and postprandial 0-24h ileal fluid | Phloretin-2’-O-GlcA  Phloretin-2’-O-GlcA, phloretin-O- GlcA, phloretin-O-GlcA-O-Sulf, Total phloretin metabolites  Phloretin-2’-(2’’-*O*-xylosyl)-glucoside, phloretin-*O*-(*O*-xylosyl)hexoside, phloretin-2’-*O*-GlcA, phloretin-O-GlcA, phloretin-*O*- GlcA-*O*-Sulf, phloretin*-O*-Sulf, total phloretin metabolites | [93] |
| Cider (1.1 L) | Intervention time-response after single exposure | 4 males and 2 females (24-42 years) | HPLC | Pre and postprandial 0-24h urine | Phloretin | [94] |
| **Wine** |  |  |  |  |  |  |
| Wine and red wine | Cross-sectional study | 198 males and 277 females (50-61 years) | LC-MS/MS | 24h urine | RV | [95] |
| Red wine | Cross-sectional study | 481 (59% men; 55.3 ± 8.4 years) | UPLC-MS | 24h Urine | DHRV-GlcA | [96] |
| Wine | Cross-sectional study | 479 males and 521 females (66.6 ± 6.2 years) | LC-MS/MS | Morning spot urine | TRMs | [97,98] |
| Red wine | Cross-sectional study | 475 subjects (58% females) (33-77 years) | UPLC-MS/MS | 24h urine | RV | [99] |
| All wine, red wine | Cross-sectional study | 475 subjects (41.7 men, 53.9 ± 8.5 years) | UPLC-MS/MS | 24h urine | RV | [100] |
| Wine | Cross-sectional study | 230 females (66.9 ± 0.4 years) | LC-MS/MS | Urine | Tartaric acid | [101] |
| Wine | Cross-sectional study | 25 subjects (25-55 years) | HPLC-MS/MS | Morning fasting plasma | *Cis*-RV, *trans*-RV | [102] |
| Red wine (250 mL) *vs.* grape juice (1 L) vs. tablets of red wine extracts (10 tablets) | 3 x intervention randomized, cross-over, double-blind, time-response after single exposure | 11 males (19-24 years) | GC-MS  GC-MS | Pre and postprandial 0-24h plasma  Pre and postprandial 0-24h urine | *Trans*-RV, *cis*-RV  *Trans*-RV, *cis-*RV, DHRV | [103] |
| Red wine *vs.* water with sugar-free artificial flavoring *vs.* ethanol (0.09% alcohol blood concentration) | 3 x intervention randomized, cross-over, single-blind, time-response after single exposure | 7 males and 6 females (mean age 35 (24-47) years) | GC-MS  GC-MS  Breath analyzer | Pre and postprandial 0-6h plasma  Pre and postprandial 0-6h urine  Pre and postprandial 0-6h breath | Free-RV  Free-RV  Ethanol | [104] |
| Red wine (375 mL) *vs*. grape extract tablets (15 tablets with 400 mL of water) | 2 x intervention cross-over, controlled, time-response after single exposure | 10 males (24-35 years) | LC-MS/MS | Pre and postprandial 0-24h urine for red wine intervention/ Pre and postprandial 0-48h urine for grape extract tablets intervention  Pre and postprandial 0-24h plasma for red wine intervention/ Pre and postprandial 0-48h plasma for grape extract tablets intervention | *Trans*-RV-3-GlcA *trans*-RV-4'-GlcA, *trans*-RV-3-Sulf, *trans*-RV-4'-Sulf , *trans*-piceid*,*  *cis*-RV-4'-GlcA, *cis*-RV-3-Sulf , cis-RV-4'-Sulf , cis-RV-3-GlcA, *cis*-piceid, piceid-GlcA, piceid-Sulfs, DHRV-GlcAs, DHRV-Sulfs  *Trans*-RV-3-GlcA *trans*-RV-4'-GlcA, *trans*-piceid*, cis*-RV-3-GlcA, *cis*-RV-4'-GlcA, *cis*-piceid, DHRV-GlcAs | [105] |
| Red wine (200 mL) | Intervention response after single exposure | 5 males (20-45 years) | LC-MS/MS | Postprandial 10h urine (morning) | Tartaric acid | [106] |
| Red wine (250 mL) | Intervention response after single exposure | 5 males (25-28 years)  11 males (18-50 years) | HPLC-MS/MS  HPLC-MS/MS | Pre and postprandial 0-4h urine  Pre and postprandial 0-24h LDL | *trans*-RV-3-GlcA, *trans*-RV-4'-GlcA, *trans*-RV-3-Sulf , *trans*-RV-4'-Sulf , *cis*-RV-3-GlcA, *cis*-RV-4'-Glc, *cis*-RV-3-Sulf, *cis-*RV-4'-Sulf  *Trans*-RV, *trans*-RV-3-GlcA, *trans*-RV-4'-GlcA, *trans*-RV-3-Sulf, *trans*-RV-4'-Sulf , *cis*-RV-3-GlcA, *cis-*RV-3-Sulf, *cis-*RV-4'-Sulf | [107] |
| Wine | Intervention response after single exposure | 2 subjects (sex and age n.p.) | n.p. | Urine | Tartaric acid | [108] |
| Red wine (150 mL) *vs.* olive oil (25 mL) *vs.* combination of both | 3 x intervention randomized, cross-over, response after single exposure | 6 males and 6 females (34.0 ± 10.5 years) | HPLC-MS | Pre (2 prior the test-0h) and postprandial 0-6h urine | *Cis*-RV, *trans*-RV, DHRV | [109] |
| Dealcoholized red wine (100 mL) *vs.* dealcoholized red wine enriched with non-encapsulated (100 mL) *vs.* encapsulated phenolic extract | 3 x intervention randomized, cross-over, single-blind, controlled, time-response after single exposure | 6 females and 6 males (19-50 years) | UPLC-MS/MS  UPLC-MS/MS | Pre and postprandial 0-6h plasma Pre and postprandial 0-24h urine | RV-Sulf, RV-GlcA  RV-Sulf, RV-GlcA | [110] |
| Red wine (100, 200, 300 mL) | 3 x intervention cross-over, controlled, dose-response after single exposure | 21 males (21-50 years) | LC-MS/MS | Morning spot urine | Tartaric acid | [111] |
| Study 1: Red wine and standard meal (300 mL)  Study 2: Red wine fasting (600 mL)  Study 3: red wine and two different meals (differing in the lipid content) (600 mL) | Intervention time-response after single exposure Intervention time-response after single exposure Intervention parallel, time-response after single exposure | 10 males (25-40 years)  1 male and 4 females (24-38 years)  3 males and 7 females (24-54 years) | LC-UV-DAD  HPLC-MS/MS  HPLC-MS/MS | Pre and postprandial 0-4h serum  Pre and postprandial 0-4h serum  Pre and postprandial 0-4h serum | *Trans*-RV-3-GlcA, *trans*-RV-4'-GlcA  Free-*trans*-RV, *trans*-RV-3-GlcA, *trans*-RV-4'-GlcA  Free-*trans*-RV, *trans*-RV-3-GlcA, *trans*-RV-4'-GlcA | [112] |
| Study 1: Red wine (272 mL/day) *vs.* dealcoholized red wine (272 mL/day) *vs.* gin (100 mL/day)  Study 2: Wine | 3 x 4w intervention randomized, cross-over, controlled, response after repeated exposure  Cross-sectional study | 56 subjects (≥ 55 years)  91 (53-79 years) | ^1^H-NMR  ^1^H-NMR | 24h urine  Urine | Ethanol, tartaric acid, EtG  Combined biomarker: tartaric acid, EtG  Ethanol, tartaric acid, EtG  Combined biomarker: tartaric acid, EtG | [113] |
| Red wine (272 mL/day) *vs.* dealcoholized red wine (272 mL/day) *vs.* gin (100 mL/day) | 3 x 4w intervention randomized, cross-over, open, controlled, response after repeated exposure | 67 males (60 ± 8 years) | HPLC-MS/MS  LC-MS | 24h urine  24h urine | *Cis*-RV, *trans*-RV, Total RVs  EtG | [114–116] |
| Red wine (272 mL/day) *vs.* dealcoholized red wine (272 mL/day) *vs.* gin (100 mL/day) | 3 x 4w intervention randomized, cross-over, controlled, response after repeated exposure | 61 subjects (≥ 55 years) | ^1^H-NMR | 24h urine | Tartaric acid, ethanol | [117] |
| Study 1: Red wine (200 mL/day) *vs.* white wine (200 mL/day)  Study 2: Wine | 3 x 4w intervention randomized, cross-over, single-blinded, controlled, response after repeated exposure  Cross-sectional study | 10 females (38.1 ± 9.2 years)  30 males and 22 females (55-80 years) | LC-MS/MS  LC-MS/MS | Morning spot urine  Morning spot urine | *Trans*-RV-3-GlcA, *cis*-RV-3-GlcA, TRMs (*trans*-RV-GlcA, *cis*-RV-GlcA)  *Trans*-RV-3-GlcA, *cis*-RV-3-GlcA, TRMs (*trans*-RV-GlcA, *cis*-RV-GlcA) | [118] |
| Red wine (272 mL/day) *vs.* dealcoholized red wine (272 mL/day) *vs.* gin (100 mL/day) | 3 x 4w cross-over, controlled, RCT | 36 males (61 ± 9 years) | UPLC-MS/MS | 24h urine | TRMs, total RV microbial metabolites | [119] |
| Red wine (200 mL/day) *vs.* white wine (200 mL/day) | 2 x 4w intervention randomized, cross-over, controlled, response after repeated exposure | 35 females (20-50 years) | HPLC | Spot urine | TRMs (*trans*-RV-GlcA, *cis*-RV-GlcA) | [120] |
| Red wine (272 mL/day) *vs.* dealcoholized red wine (272 mL/day) | 2 x 4w intervention randomized, cross-over, controlled, response after repeated exposure | 59 subjects (≥ 55 years) | UPLC-MS/MS | 24h urine | *Trans*-RV-3-GlcA, *trans*-RV-4-GlcA, *trans*-RV-3-Sulf, *trans*-RV-4-Sulf, *trans*-RV-3,4-diSulf, *cis*-RV-3-GlcA, *cis*-RV-4-GlcA, *cis*-RV-3-GlcA, *cis*-RV-4-Sulf, *cis*-RV-3-Sulf, RV-Sulf-GlcA, piceid-GlcA, piceid-Sulfs, DHRV, DHRV-GlcA, DHRV-Sulf, DHRV-Sulf-GlcA, TRMs, total DHRVs | [121] |
| Red wine (250 mL/day) *vs.* control | 2 x 4w intervention randomized, parallel, controlled, response after repeated exposure | 22 females and 19 males (36 ± 11 years) | UHPLC-QTOF-MS | 24h urine | Tartaric acid, EtS | [122] |
| Red wine (270 mL/day) | 4w intervention response after repeated exposure | 6 males and 4 females (40.4 ± 4.1 years) | UHPLC-MS/MS | Fasting plasma | *Cis*-RV-4-Sulf, DHRV-3-Sulf | [123] |
| Aged white wine (255 mL/day) *vs.* gin (92 mL/day) | 2 x 3w intervention randomized, cross-over, open, controlled, response after repeated exposure | 38 males (55-80 years) | LC-ESMS/MS | 24h urine | Tartaric acid | [124,125] |
| Red wine (272 mL/day) *vs.* dealcoholized red wine (272 mL/day) | 3 x 20d intervention randomized, cross-over, controlled, response after repeated exposure | 10 males (48 ± 2 years) | HPLC-MS/MS | 24h Urine | TRMs, total DHRV metabolites, total metabolites (RV+DHRV metabolites) | [126,127] |
| Red wine (300 mL/day) *vs.* white wine (300 mL/day)  *vs.* control | 15d intervention randomized, parallel, controlled, response after repeated exposure | 9 males and 11 females (around 40 years) | HPLC | Fasting plasma | RV | [128,129] |
| **Spirits and distillates** |  |  |  |  |  |  |
| Study 1: aniseed spirit (Helenas Ouzu) (120, 200, 360 mL)  Study 2: aniseed spirit (Ouzo, Raki or Küstennebel) | Intervention dose and time-response after single exposure Cross-sectional study | 1 male (22 years)  10 females and 40 males (17-57 years) drivers | HS-SPME-GC-MS  HS-SPME-GC-MS | Pre and postprandial 0-24h serum  Serum | Anethole  Anethole, ethanol | [130] |
| Study 1: peppermint liquor (160, 320 and 560 mL)  Study 2: peppermint liquor | Intervention dose and time-response after single exposure  Cross-sectional study | 1 male (29 years)  5 females and 95 males (18-66 years) | HS-SPME-GC-MS  HS-SPME-GC-MS | Pre and postprandial 0-24h serum  Serum | Menthone, isomenthone, neomenthol, menthol  Menthone, isomenthone, neomenthol, menthol, ethanol | [131] |

Abbreviatures: ^1^H-NMR, Proton Nuclear Magnetic Resonance; 5-HIAA, 5-hydroxyindoleacetic acid; 5-HTOL, 5-hydroxytryptophol; DHRV, dihydroresveratrol; d, days; ELSD, Evaporative Light-Scattering Detection; EtG, Ethyl Glucuronide; EtS. Ethyl Sulfate; FAEEs, Free Acids Ethyl Esters; GC-MS, Gas Chromatography–Mass Spectrometry; GlcA, Glucuronide; h; hours; HDL, High Density Lipoprotein; HPLC-MS/MS, High Performance Liquid Chromatography-Tandem Mass Spectrometry; HPLC-PDA-MSn, High Performance Liquid Chromatography-Photodiode Array-Mass Spectrometry; HS-GC, Headspace Gas Chromatography; HS-GC-FID, Headspace Gas Chromatography with Flame Ionization Detection; HS-SPME-GC-MS,  Headspace Solid-phase Microextraction Gas Chromatography; IAAs, Iso-α-Acids; LC-ESI-MS/M, Liquid Chromatography- Electrospray Ionization-Tandem Mass Spectrometry LC-MS/MS, Liquid Chromatography-Tandem Mass Spectrometry; LC-UV-DAD, Liquid Chromatography-Ultraviolet-Diode Array Detection; LDL, Low Density Lipoprotein; n.p., not provided; PEths, Phosphatidylethanols; RCT, Randomized Clinical Trial; RV, resveratrol; TRMs, Total Resveratrol Metabolites; Sulf, sulfate; Sulf-GlcA, sulfoglucuronide; UHPLC, Ultra-High-Performance Liquid Chromatography; UHPLC-QTOF- MS, Ultrahigh-Performance Liquid Chromatography−Time-of-Flight Mass Spectrometry; UPC2-MS/MS, Ultra Performance Convergence chromatography-Tandem Mass Spectrometry; VLDL, Very Low-Density Lipoprotein; w, weeks.

^1^Study design listed as the way it was analyzed (not necessarily using the design of the primary study), e.g., prospective studies are typically analyzed cross-sectionally at baseline.

**Supplementary Table 3.** Summary of the excluded candidate BFIs of alcoholic beverages subgroups and ethanol consumption and reasons for exclusion.

| Dietary factor | Metabolites | Biofluid locations | Reason for inclusion and exclusion | Reference |
| --- | --- | --- | --- | --- |
| **Alcohol** | 5-HTOL and related metabolites | Blood/Urine | Possibly unspecific. Only investigated as a marker of alcohol abuse | [56] |
|  | Propanol | Blood/Urine | Formed from several sources (not robust) | [132] |
|  | GGT, ALT, AST | Blood | Unspecific, possible marker of effect | [133] |
|  | MCV, CDT, SIJ | Blood | Unspecific, possible marker of effect | [134] |
|  | EDAC | Blood | Only validated as a categorical marker for problem drinking | [135] |
|  | HDL and related markers | Blood | Unspecific at the individual level but sensitive above ~1 drink a day at the group level. Possible marker of effect | [136] |
| **Beer** | 8-Prenylnaringenine | Urine/Plasma | High inter-individual variability | [91,137] |
|  | Sphingomyelin | Serum | Possible biomarker of effect | [138] |
|  | Free tyrosol and hydroxytyrosol | Urine | Not specific as beer intake biomarker (e.g., olive oil and wine) | [139] |
|  | Proline Betaine | Urine | Much lower concentration than citrus fruits | [140] |
|  | 16-Hydroxypalmitate | Serum | Possible biomarker of effect | [12] |
|  | 5-Hydroxymethylfurfural and 5-hydroxymethylfurfural-2-furoic acid | Urine | Not specific as beer intake biomarker (e.g., coffee, and dried fruits) | [141] |
|  | Mevalonic acid | Urine | Possible biomarker of effect | [142] |
|  | l-Methyl-l,2,3,4-tetrahydro-β-carboline | Urine | Not specific as beer intake biomarker (e.g., wine) | [143] |
|  | l,2,3,4-tetrahydro-β-carboline | Urine | Not specific as beer intake biomarker (e.g., wine, banana) | [143] |
| **Cider** | Isorhamnetin (3’-methyl quercetin) | Plasma | Not specific as cider intake biomarker (e.g., grapefruit, orange juice, cranberry juice, almond extract, onion, sea buckthorn, tomato puree) | [94] |
|  | Tamarixetin (4’-methyl quercetin) | Plasma | Not specific as cider intake biomarker (e.g., onion) | [94] |
|  | Caffeic Acid | Plasma | Not specific as cider intake biomarker (e.g., coffee, olive oil, tomato, wine, cocoa, artichoke, berry) | [94] |
|  | Hippuric Acid | Urine | Not specific as cider intake biomarker (e.g., gut microbial fermentation product after consumption of tea, chamomile, wine, coffee, fruit juice) | [94] |
| **Wine** |  |  |  |  |
| Tyrosols | Free tyrosol, hydroxytyrosol and derivatives | Urine/Plasma | Not specific as wine intake biomarker (e.g., olive oil and beer) | [96,109,110,144,145] |
| Anthocyanins | Anthocyanins^1^ | Urine/Plasma | Not specific as wine intake biomarker (e.g., berries) | [110,120,146–149] |
| Flavanols | Catechin and derivatives | Urine/Plasma | Not specific as wine intake biomarker (e.g., apple, apricot, black tea, cocoa) | [99,104,110,119,128,150–155] |
|  | Epicatechin and derivatives | Urine/Plasma | Not specific as wine intake biomarker (e.g., apple, black tea, green tea, cocoa) | [110,119,150,152,156–158] |
| Flavonols | Isorhamnetin | Urine/Plasma | Not specific as wine intake biomarker (e.g., onion, berries) | [159] |
|  | Kaempferol | Urine | Not specific as wine intake biomarker (e.g., berries, tea, spices) | [159] |
|  | Quercetin | Urine/Plasma | Not specific as wine intake biomarker (e.g., onion, green tea, chocolate) | [159] |
| Hydroxyphenylacetic acids | Di- and hydroxyphenyl acetic acid and derivatives | Urine/Feces | Not specific as wine intake biomarker (e.g., oat, maize, olive oil) | [99,110,117,119,150,160–162] |
| Hydroxybenzoic acids | Hippuric acid | Urine | Not specific as wine intake biomarker (e.g., tea, fruit juices, whole grain) | [117,150] |
|  | Syringic acid and derivatives | Urine/Plasma  /Feces | Not specific as wine intake biomarker (e.g., walnuts, olive, date) | [96,110,119,150,161–163] |
|  | Gallic acid and derivatives | Urine | Not specific as wine intake biomarker (e.g., cocoa, coffee) | [96,99,110,119,151,164] |
|  | 4-O-methylgallic acid | Urine/Plasma | Not specific as wine intake biomarker (e.g., tea, grapes) | [96,151,164–167] |
|  | Hydroxybenzoic acid derivatives | Urine/Feces | Not specific as wine intake biomarker (e.g., berries, grapefruit, date, cereals, beer, coconut) | [119,150,162,163] |
| Hydroxycinnamic acid | Sinapic acid | Urine | Not specific as wine intake biomarker (e.g., strawberry guava, ryes, cauliflower) | [150] |
|  | Caffeic acid and derivatives | Urine/Plasma | Not specific as wine intake biomarker (e.g., berries, dried fruits, seeds, olive, potato) | [110,119,128,150,151,164,165,168–170] |
|  | m-Coumaric acid and derivatives | Urine | Not specific as wine intake biomarker (e.g., olive, corns, beer, whole grain) | [96,150] |
|  | p-Coumaric acid | Urine/Plasma  /Feces | Not specific as wine intake biomarker (e.g., coriander, peanut, date) | [99,119,150,163,168] |
|  | Ferulic acid and derivatives | Urine/Plasma | Not specific as wine intake biomarker (e.g., cocoa, dried fruits, cereal products) | [110,119,150,166,168] |
|  | Caftaric acid | Plasma | Very low or undetectable concentration | [168] |
|  | Fertaric acid | Plasma | undetectable concentration | [168] |
| Other class of compounds | Malic acid | Urine | Not specific as wine intake biomarker (e.g., apple, apricots, berries, plums, cherries) and produced endogenous | [171] |
|  | Succinic acid | Urine | Endogenous metabolite | [171] |
|  | Scyllo-inositol | Serum | Not specific as wine intake biomarker (e.g., coconut, citrus fruits) | [12] |
| **Sweet wine** | *Cis*-resveratrol-3-*O*-glucuronides | Urine | Not specific as sweet or sparkling wine intake biomarker (e.g., all wine) | [118] |
|  | *Trans*-resveratrol-3-*O*-glucuronides | Urine | Not specific as sweet or sparkling wine intake biomarker (e.g.., all wine) | [118] |
| **Spirits and distillates** | TTCA | Urine | Not specific as liquor intake biomarker (e.g., environment, cruciferous vegetables) | [172] |

Abbreviations: 5-HTOL, 5-hydroxytryptophol; ALT, ALanine aminoTransferase; AST, ASpartate aminoTransferase; CDT, Carbohydrate Deficient Transferrin; EDAC, Early Detection od Alcohol Cunsumption (a combined marker); GGT, Gamma Glutamyl Transferase; HDL, High Density Lipoprotein (also subfractions and apolipoprotein A1); MCV, Mean Corpuscular Volume of erythrocytes; SIJ, Sialic acid Index of apolipoprotein J; TTCA, 2-thiothiazolidine-4-carboxylic acid.

^1^ Cyanidin-3-glucoside, delphinidin-3-glucoside, malvidin-3-glucoside, peonidin-3-glucoside, petunidin-3-glucoside, total anthocyanins.

**Bibliography**

1. Årving A, Høiseth G, Hilberg T, Trydal T, Husa A, Djordjevic A, Kabashi S, Vindenes V, Bogstrand ST. Comparison of the Diagnostic Value of Phosphatidylethanol and Carbohydrate-Deficient Transferrin as Biomarkers of Alcohol Consumption. Alcohol Clin Exp Res. 2021; 45: 153–62. doi: 10.1111/acer.14503.

2. van de Luitgaarden IAT, Schrieks IC, Kieneker LM, Touw DJ, van Ballegooijen AJ, van Oort S, Grobbee DE, Mukamal KJ, Kootstra-Ros JE, Kobold ACM, Bakker SJL, Beulens JWJ. Urinary ethyl glucuronide as measure of alcohol consumption and risk of cardiovascular disease: A population-based cohort study. J Am Heart Assoc. 2020; 9. doi: 10.1161/jaha.119.014324.

3. Finanger T, Spigset O, Gråwe RW, Andreassen TN, Løkken TN, Aamo TO, Bratt GE, Tømmervik K, Langaas VS, Finserås K, Salvesen KÅB, Skråstad RB. Phosphatidylethanol as Blood Biomarker of Alcohol Consumption in Early Pregnancy: An Observational Study in 4,067 Pregnant Women. Alcohol Clin Exp Res. 2021; 45: 886–92. doi: 10.1111/acer.14577.

4. Kriikku P, Wilhelm L, Jenckel S, Rintatalo J, Hurme J, Kramer J, Wayne Jones A, Ojanperä I. Comparison of breath-alcohol screening test results with venous blood alcohol concentration in suspected drunken drivers. Forensic Sci Int. 2014; 239: 57–61. doi: 10.1016/j.forsciint.2014.03.019.

5. Suesse S, Pragst F, Mieczkowski T, Selavka CM, Elian A, Sachs H, Hastedt M, Rothe M, Campbell J. Practical experiences in application of hair fatty acid ethyl esters and ethyl glucuronide for detection of chronic alcohol abuse in forensic cases. Forensic Sci Int. 2012; 218: 82–91. doi: 10.1016/j.forsciint.2011.10.006.

6. Langenau J, Oluwagbemigun K, Brachem C, Lieb W, di Giuseppe R, Artati A, Kastenmüller G, Weinhold L, Schmid M, Nöthlings U. Blood metabolomic profiling confirms and identifies biomarkers of food intake. Metabolites. 2020; 10: 1–17. doi: 10.3390/metabo10110468.

7. Wang Y, Gapstur SM, Carter BD, Hartman TJ, Stevens VL, Gaudet MM, McCullough ML. Untargeted Metabolomics Identifies Novel Potential Biomarkers of Habitual Food Intake in a Cross-Sectional Study of Postmenopausal Women. J Nutr. 2018; 148: 932–43. doi: 10.1093/jn/nxy027.

8. Schröder H, de La Torre R, Estruch R, Corella D, Martínez-González MA, Salas-Salvadó J, Ros E, Arós F, Flores G, Civit E, Farré M, Fiol M, Vila J, et al. Alcohol consumption is associated with high concentrations of urinary hydroxytyrosol. A J Clin Nutr. 2009; 90: 1329–35. doi: 10.3945/ajcn.2009.27718.

9. Süße S, Selavka CM, Mieczkowski T, Pragst F. Fatty acid ethyl ester concentrations in hair and self-reported alcohol consumption in 644 cases from different origin. Forensic Sci Int. 2010; 196: 111–7. doi: 10.1016/j.forsciint.2009.12.029.

10. Kwak H-S, Han J-Y, Choi J-S, Ahn H-K, Ryu H-M, Chung H-J, Cho D-H, Shin C-Y, Velazquez-Armenta EY, Nava-Ocampo AA. Characterization of phosphatidylethanol blood concentrations for screening alcohol consumption in early pregnancy. Clin Toxicol. 2014; 52: 25–31. doi: 10.3109/15563650.2013.859263.

11. Jones J, Jones M, Plate C, Lewis D, Fendrich M, Berger L, Fuhrmann D. Liquid Chromatography-Tandem Mass Spectrometry Assay to Detect Ethyl Glucuronide in Human Fingernail: Comparison to Hair and Gender Differences. Am J Analyt Chem. 2012; 03: 83–91. doi: 10.4236/ajac.2012.31012.

12. Guertin KA, Moore SC, Sampson JN, Huang WY, Xiao Q, Stolzenberg-Solomon RZ, Sinha R, Cross AJ. Metabolomics in nutritional epidemiology: Identifying metabolites associated with diet and quantifying their potential to uncover diet-disease relations in populations. Am J Clin Nutr. 2014; 100: 208–17. doi: 10.3945/ajcn.113.078758.

13. Neumann J, Beck O, Helander A, Böttcher M. Performance of PEth Compared with Other Alcohol Biomarkers in Subjects Presenting for Occupational and Pre-Employment Medical Examination. Alcohol Alcohol. 2020; 55: 401–8. doi: 10.1093/alcalc/agaa027.

14. Raggio GA, Psaros C, Fatch R, Goodman G, Matthews LT, Magidson JF, Amanyire G, Cross A, Asiimwe S, Hahn JA, Haberer JE. High Rates of Biomarker-Confirmed Alcohol Use among Pregnant Women Living with HIV in South Africa and Uganda. J Acquir Immune Defic Syndr. 2019; 82: 443–51. doi: 10.1097/qai.0000000000002156.

15. Zuba D. Accuracy and reliability of breath alcohol testing by handheld electrochemical analysers. Forensic Sci Int. 2008; 178: e29–33. doi: 10.1016/j.forsciint.2008.03.002.

16. Schröck A, Wurst FM, Thon N, Weinmann W. Assessing phosphatidylethanol (PEth) levels reflecting different drinking habits in comparison to the alcohol use disorders identification test – C (AUDIT-C). Drug Alcohol Depend. 2017; 178: 80–6. doi: 10.1016/j.drugalcdep.2017.04.026.

17. Johansson K, Johansson L, Pennlert J, Söderberg S, Jansson JH, Lind MM. Phosphatidylethanol Levels, As a Marker of Alcohol Consumption, Are Associated with Risk of Intracerebral Hemorrhage. Stroke. 2020; 51: 2148–52. doi: 10.1161/strokeaha.120.029630.

18. Couture MC, Page K, Sansothy N, Stein E, Vun MC, Hahn JA. High prevalence of unhealthy alcohol use and comparison of self-reported alcohol consumption to phosphatidylethanol among women engaged in sex work and their male clients in Cambodia. Drug Alcohol Depend. 2016; 165: 29–37. doi: 10.1016/j.drugalcdep.2016.05.011.

19. Pragst F, Rothe M, Moench B, Hastedt M, Herre S, Simmert D. Combined use of fatty acid ethyl esters and ethyl glucuronide in hair for diagnosis of alcohol abuse: Interpretation and advantages. Forensic Sci Int. 2010; 196: 101–10. doi: 10.1016/j.forsciint.2009.12.028.

20. Skipper GE, Weinmann W, Thierauf A, Schaefer P, Wiesbeck G, Allen JP, Miller M, Wurst FM. Ethyl glucuronide: A biomarker to identify alcohol use by health professionals recovering from substance use disorders. Alcohol Alcohol. 2004; 39: 445–9. doi: 10.1093/alcalc/agh078.

21. Soderberg BL, Salem, MS RO, Best, MS CA, Cluette-Brown, MS JE, Laposata, MD, PhD M. Fatty Acid Ethyl Esters: Ethanol Metabolites That Reflect Ethanol Intake. Path Patterns Rev. 2003; 119: 94–9. doi: 10.1309/6f39ear2l4gyx5g6.

22. Kintz P, Nicholson D. Testing for ethanol markers in hair: Discrepancies after simultaneous quantification of ethyl glucuronide and fatty acid ethyl esters. Forensic Sci Int. 2014; 243: 44–6. doi: 10.1016/j.forsciint.2014.03.012.

23. Albermann ME, Musshoff F, Madea B. A fully validated high-performance liquid chromatography-tandem mass spectrometry method for the determination of ethyl glucuronide in hair for the proof of strict alcohol abstinence. Anal Bioanal Chem. 2010; 396: 2441–7. doi: 10.1007/s00216-009-3388-2.

24. Pragst F, Yegles M. Determination of fatty acid ethyl esters (FAEE) and ethyl glucuronide (EtG) in hair: A promising way for retrospective detection of alcohol abuse during pregnancy? Ther Drug Monit. 2008; 30: 255–63. doi: 10.1097/ftd.0b013e318167d602.

25. Cappelle D, Neels H, Yegles M, Paulus J, van Nuijs ALN, Covaci A, Crunelle CL. Gas chromatographic determination of ethyl glucuronide in hair: Comparison between tandem mass spectrometry and single quadrupole mass spectrometry. Forensic Sci Int. 2015; 249: 20–4. doi: 10.1016/j.forsciint.2014.11.022.

26. Wang S, Yang R, Ji F, Li H, Dong J, Chen W. Sensitive and precise monitoring of phosphatidylethanol in human blood as a biomarker for alcohol intake by ultrasound-assisted dispersive liquid-liquid microextraction combined with liquid chromatography tandem mass spectrometry. Talanta. 2017; 166: 315–20. doi: 10.1016/j.talanta.2017.01.083.

27. Pirro V, di Corcia D, Seganti F, Salomone A, Vincenti M. Determination of ethyl glucuronide levels in hair for the assessment of alcohol abstinence. Forensic Sci Int. 2013; 232: 229–36. doi: 10.1016/j.forsciint.2013.07.024.

28. Auwärter V, Sporkert F, Hartwig S, Pragst F, Vater H, Diefenbacher A. Fatty acid ethyl esters in hair as markers of alcohol consumption. Segmental hair analysis of alcoholics, social drinkers, and teetotalers. Clin Chem. 2001; 47: 2114–23. doi: 10.1093/clinchem/47.12.2114.

29. Yegles M, Labarthe A, Auwärter V, Hartwig S, Vater H, Wennig R, Pragst F. Comparison of ethyl glucuronide and fatty acid ethyl ester concentrations in hair of alcoholics, social drinkers and teetotallers. Forensic Sci Int. 2004; 145: 167–73. doi: 10.1016/j.forsciint.2004.04.032.

30. Pragst F, Auwärter V, Kießling B, Dyes C. Wipe-test and patch-test for alcohol misuse based on the concentration ratio of fatty acid ethyl esters and squalene CFAEE/CSQ in skin surface lipids. Forensic Sci Int. 2004; 143: 77–86. doi: 10.1016/j.forsciint.2004.02.041.

31. Hartwig S, Auwärter V, Pragst F. Fatty acid ethyl esters in scalp, pubic, axillary, beard and body hair as markers for alcohol misuse. Alcohol Alcohol. 2003; 38: 163–7. doi: 10.1093/alcalc/agg046.

32. Mabuchi R, Kurita A, Miyoshi N, Yokoyama A, Furuta T, Goda T, Suwa Y, Kan T, Amagai T, Ohshima H. Analysis of Nε-Ethyllysine in Human Plasma Proteins by Gas Chromatography–Negative Ion Chemical Ionization/Mass Spectrometry as a Biomarker for Exposure to Acetaldehyde and Alcohol. Alcohol Clin Exp Res. 2012; 36: 1013–20. doi: 10.1111/j.1530-0277.2011.01705.x.

33. Morfin JP, Kulig C, Everson G, Beresford T. Controlling for serum albumin level improves the correlation between serum fatty acid ethyl esters and blood ethanol level. Alcohol Clin Exp Res. 2007; 31: 265–8. doi: 10.1111/j.1530-0277.2006.00302.x.

34. Casati S, Ravelli A, Angeli I, Durello R, Minoli M, Orioli M. An automated sample preparation approach for routine liquid chromatography tandem-mass spectrometry measurement of the alcohol biomarkers phosphatidylethanol 16:0/18:1, 16:0/16:0 and 18:1/18:1. J Chromatogr A. 2019; 1589: 1–9. doi: 10.1016/j.chroma.2018.12.048.

35. Liu Y, Zhang X, Li J, Huang Z, Lin Z, Wang J, Zhang C, Rao Y. Stability of ethyl glucuronide, ethyl sulfate, phosphatidylethanols and fatty acid ethyl esters in postmortem human blood. J Anal Toxicol. 2018; 42: 346–52. doi: 10.1093/jat/bky010.

36. de Benedetto GE, Fanigiliulo M. A new CE-ESI-MS method for the detection of stable hemoglobin acetaldehyde adducts, potential biomarkers of alcohol abuse. Electrophoresis. 2009; 30: 1798–807. doi: 10.1002/elps.200800379.

37. Bird DA, Kabakibi A, Laposata M. The Distribution of Fatty Acid Ethyl Esters among Lipoproteins and Albumin in Human Serum. Alcohol Clin Exp Res. 1997; 21: 602–5. doi: 10.1111/j.1530-0277.1997.tb03809.x.

38. Fleming MF, Smith MJ, Oslakovic E, Lucey MR, Vue JX, Al-Saden P, Levitsky J. Phosphatidylethanol Detects Moderate-to-Heavy Alcohol Use in Liver Transplant Recipients. Alcohol Clin and Expe Res. 2017; 41: 857–62. doi: 10.1111/acer.13353.

39. Gutierrez HL, Hund L, Shrestha S, Rayburn WF, Leeman L, Savage DD, Bakhireva LN. Ethylglucuronide in maternal hair as a biomarker of prenatal alcohol exposure. Alcohol. 2015; 49: 617–23. doi: 10.1016/j.alcohol.2015.06.002.

40. Borucki K, Kunstmann S, Dierkes J, Westphal S, Diekmann S, Bogerts B, Luley C. In heavy drinkers fatty acid ethyl esters in the serum are increased for 44 hr after ethanol consumption. Alcohol Clin Exp Res. 2004; 28: 1102–6. doi: 10.1097/01.alc.0000130791.20186.4d.

41. Pragst F, Suesse S, Salomone A, Vincenti M, Cirimele V, Hazon J, Tsanaclis L, Kingston R, Sporkert F, Baumgartner MR. Commentary on current changes of the SoHT 2016 consensus on alcohol markers in hair and further background information. Forensic Sci Int. 2017; 278: 326–33. doi: 10.1016/j.forsciint.2017.07.023.

42. Oppolzer D, Barroso M, Passarinha L, Gallardo E. Determination of ethyl glucuronide and fatty acid ethyl esters in hair samples. Biomed Chromatogr. 2017; 31: 1–12. doi: 10.1002/bmc.3858.

43. Gnann H, Thierauf A, Hagenbuch F, Röhr B, Weinmann W. Time Dependence of Elimination of Different PEth Homologues in Alcoholics in Comparison with Social Drinkers. Alcohol Clin Exp Res. 2014; 38: 322–6. doi: 10.1111/acer.12277.

44. Rosano TG, Lin J. Ethyl glucuronide excretion in humans following oral administration of and dermal exposure to ethanol. J Anal Toxicol. 2008; 32: 594–600. doi: 10.1093/jat/32.8.594.

45. Helander A, Hermansson U, Beck O. Dose-Response Characteristics of the Alcohol Biomarker Phosphatidylethanol (PEth)-A Study of Outpatients in Treatment for Reduced Drinking. Alcohol Alcohol. 2019; 54: 567–73. doi: 10.1093/alcalc/agz064.

46. Helander A, Böttcher M, Dahmen N, Beck O. Elimination Characteristics of the Alcohol Biomarker Phosphatidylethanol (PEth) in Blood during Alcohol Detoxification. Alcohol Alcohol. 2019; 54: 251–7. doi: 10.1093/alcalc/agz027.

47. Luginbühl M, Weinmann W, Butzke I, Pfeifer P. Monitoring of direct alcohol markers in alcohol use disorder patients during withdrawal treatment and successive rehabilitation. Drug Test  Anal. 2019; 11: 859–69. doi: 10.1002/dta.2567.

48. Doyle KM, Cluette-Brown JE, Dube DM, Bernhardt TG, Morse CR, Laposata M. Fatty acid ethyl esters in the blood as markers for ethanol intake. J Am Med Assoc. 1996; 276: 1152–6. doi: 10.1001/jama.276.14.1152.

49. Albermann ME, Musshoff F, Doberentz E, Heese P, Banger M, Madea B. Preliminary investigations on ethyl glucuronide and ethyl sulfate cutoffs for detecting alcohol consumption on the basis of an ingestion experiment and on data from withdrawal treatment. Int J Legal Med. 2012; 126: 757–64. doi: 10.1007/s00414-012-0725-3.

50. Borucki K, Dierkes J, Wartberg J, Westphal S, Genz A, Luley C. In heavy drinkers, fatty acid ethyl esters remain elevated for up to 99 hours. Alcohol Clin Exp Res. 2007; 31: 423–7. doi: 10.1111/j.1530-0277.2006.00323.x.

51. Christopher TA, Zeccardi JA. Evaluation of the Q.E.D.^TM^ saliva alcohol test: A new, rapid, accurate device for measuring ethanol in saliva. Ann Emerg Med. Mosby; 1992; 21: 1135–7. doi: 10.1016/s0196-0644(05)80659-6.

52. Huppertz LM, Gunsilius L, Lardi C, Weinmann W, Thierauf-Emberger A. Influence of Gilbert’s syndrome on the formation of ethyl glucuronide. Int J Legal Med. 2015; 129: 1005–10. doi: 10.1007/s00414-015-1157-7.

53. Borucki K, Schreiner R, Dierkes J, Jachau K, Krause D, Westphal S, Wurst FM, Luley C, Schmidt-Gayk H. Detection of recent ethanol intake with new markers: Comparison of fatty acid ethyl esters in serum and of ethyl glucuronide and the ratio of 5-hydroxytryptophol to 5-hydroxyindole acetic acid in urine. Alcohol Clin Exp Res. 2005; 29: 781–7. doi: 10.1097/01.alc.0000164372.67018.ea.

54. Halter CC, Dresen S, Auwaerter V, Wurst FM, Weinmann W. Kinetics in serum and urinary excretion of ethyl sulfate and ethyl glucuronide after medium dose ethanol intake. Int J Legal Med. 2008; 122: 123–8. doi: 10.1007/s00414-007-0180-8.

55. Maluenda F, Csendes A, de Aretxabala X, Poniachik J, Salvo K, Delgado I, Rodriguez P. Alcohol absorption modification after a laparoscopic sleeve gastrectomy due to obesity. Obes Surg. 2010; 20: 744–8. doi: 10.1007/s11695-010-0136-9.

56. Helander A, Beck O, Jacobsson G, Löwenmo C, Wikström T. Time course of ethanol-induced changes in serotonin metabolism. Life Sci. 1993; 53: 847–55. doi: 10.1016/0024-3205(93)90507-y.

57. Shindyapina A v., Petrunia I v., Komarova T v., Sheshukova E v., Kosorukov VS, Kiryanov GI, Dorokhov YL. Dietary methanol regulates human gene activity. PLoS One. 2014; 9: e102837. doi: 10.1371/journal.pone.0102837.

58. Luginbühl M, Schröck A, König S, Schürch S, Weinmann W. Determination of fatty acid ethyl esters in dried blood spots by LC–MS/MS as markers for ethanol intake: application in a drinking study. Anal Bioanal Chem. 2016; 408: 3503–9. doi: 10.1007/s00216-016-9426-y.

59. Dresen S, Weinmann W, Wurst FM. Forensic confirmatory analysis of ethyl sulfate—A new marker for alcohol consumption—by liquid-chromatography/electrospray ionization/tandem mass spectrometry. J Am Soc Mass Spectrom. 2004; 15: 1644–8. doi: 10.1016/j.jasms.2004.08.004.

60. Schröck A, Henzi A, Bütikofer P, König S, Weinmann W. Determination of the formation rate of phosphatidylethanol by phospholipase D (PLD) in blood and test of two selective PLD inhibitors. Alcohol. 2018; 73: 1–7. doi: 10.1016/j.alcohol.2018.03.003.

61. Goll M, Schmitt G, Ganßmann B, Aderjan RE. Excretion profiles of ethyl glucuronide in human urine after internal dilution. J Anal Toxicol. 2002; 26: 262–6. doi: 10.1093/jat/26.5.262.

62. Varga A, Hansson P, Lundqvist C, Alling C. Phosphatidylethanol in blood as a marker of ethanol consumption in healthy volunteers: Comparison with other markers. Alcohol Clin Exp Res. 1998; 22: 1832–7. doi: 10.1111/j.1530-0277.1998.tb03989.x.

63. Mercurio I, Politi P, Mezzetti E, Agostinelli F, Troiano G, Pellegrino A, Gili A, Melai P, Rettagliata G, Mercurio U, Sannicandro D, Lancia M, Bacci M. Ethyl Glucuronide and Ethyl Sulphate in Urine: Caution in their use as markers of recent alcohol use. Alcohol Alcohol. 2021; 56: 201–9. doi: 10.1093/alcalc/agaa113.

64. Pérez-Mañá C, Farré M, Pastor A, Fonseca F, Torrens M, Menoyo E, Pujadas M, Frias S, Langohr K, de la Torre R. Non-linear formation of EtG and FAEEs after controlled administration of low to moderate doses of ethanol. Alcohol Alcohol. 2017; 52: 587–94. doi: 10.1093/alcalc/agx033.

65. Jacobsen D, Sebastian CS, Dies DF, Breau RL, Spann EG, Barron SK, McMartin KE. Kinetic Interactions Between 4-Methylpyrazole and Ethanol in Healthy Humans. Alcohol Clin Exp Res. 1996; 20: 804–9. doi: 10.1111/j.1530-0277.1996.tb05255.x.

66. Graham AE, Beatty JR, Rosano TG, Sokol RJ, Ondersma SJ. Utility of commercial ethyl glucuronide (EtG) and ethyl sulfate (EtS) testing for detection of lighter drinking among women of childbearing years. J Stud Alcohol Drugs. 2017; 78: 945–8. doi: 10.15288/jsad.2017.78.945.

67. Rausgaard NLK, Ravn P, Ibsen IO, Fruekilde PBN, Nohr EA, Damkier P. Clinical usefulness of a urine dipstick to detect ethyl glucuronide (EtG): A quantitative clinical study in healthy young female volunteers. Basic Clin Pharmacol Toxicol. 128: 709–15. doi: 10.1111/bcpt.13558.

68. Helander A, Beck O, Boysen L. 5-Hydroxytryptophol conjugation in man: Influence of alcohol consumption and altered serotonin turnover. Life Sci. 1995; 56: 1529–34. doi: 10.1016/0024-3205(95)00115-m.

69. Nogueira LC, Couri S, Trugo NF, Lollo PCB. The effect of different alcoholic beverages on blood alcohol levels, plasma insulin and plasma glucose in humans. Food Chem. 2014; 158: 527–33. doi: 10.1016/j.foodchem.2014.02.097.

70. Roine RP, Gentry RT, Lim RT, Helkkonen E, Salaspuro M, Lieber CS. Comparison of Blood Alcohol Concentrations After Beer and Whiskey. Alcohol Clin Exp Res. 1993; 17: 709–11. doi: 10.1111/j.1530-0277.1993.tb00824.x.

71. Høiseth G, Yttredal B, Karinen R, Gjerde H, Christophersen A. Levels of ethyl glucuronide and ethyl sulfate in oral fluid, blood, and urine after use of mouthwash and ingestion of nonalcoholic wine. J Anal Toxicol. 2010; 34: 84–8. doi: 10.1093/jat/34.2.84.

72. Javors MA, Hill-Kapturczak N, Roache JD, Karns-Wright TE, Dougherty DM. Characterization of the Pharmacokinetics of Phosphatidylethanol 16:0/18:1 and 16:0/18:2 in Human Whole Blood After Alcohol Consumption in a Clinical Laboratory Study. Alcohol Clin Exp Res. 2016; 40: 1228–34. doi: 10.1111/acer.13062.

73. Hill-Kapturczak N, Dougherty DM, Roache JD, Karns-Wright TE, Javors MA. Differences in the Synthesis and Elimination of Phosphatidylethanol 16:0/18:1 and 16:0/18:2 After Acute Doses of Alcohol. Alcohol Clin Exp Res. 2018; 42: 851–60. doi: 10.1111/acer.13620.

74. Mitchell MC, Teigen EL, Ramchandani VA. Absorption and peak blood alcohol concentration after drinking beer, wine, or spirits. Alcohol Clin Exp Res. 2014; 38: 1200–4. doi: 10.1111/acer.12355.

75. Kechagias S, Dernroth DN, Blomgren A, Hansson T, Isaksson A, Walther L, Kronstrand R, Kågedal B, Nystrom FH. Phosphatidylethanol compared with other blood tests as a biomarker of moderate alcohol consumption in healthy volunteers: A prospective randomized study. Alcohol Alcohol. 2015; 50: 399–406. doi: 10.1093/alcalc/agv038.

76. Kronstrand R, Brinkhagen L, Nyström FH. Ethyl glucuronide in human hair after daily consumption of 16 or 32g of ethanol for 3 months. Forensic Sci Int. 2012; 215: 51–5. doi: 10.1016/j.forsciint.2011.01.044.

77. Jatlow PI, Agro A, Wu R, Nadim H, Toll BA, Ralevski E, Nogueira C, Shi J, Dziura JD, Petrakis IL, O’Malley SS. Ethyl glucuronide and ethyl sulfate assays in clinical trials, interpretation, and limitations: Results of a dose ranging alcohol challenge study and 2 clinical trials. Alcohol Clin Exp Res. 2014; 38: 2056–65. doi: 10.1111/acer.12407.

78. Balbo S, Meng L, Bliss RL, Jensen JA, Hatsukami DK, Hecht SS. Time course of DNA adduct formation in peripheral blood granulocytes and lymphocytes after drinking alcohol. Mutagenesis. 2012; 27: 485–90. doi: 10.1093/mutage/ges008.

79. Jung SJ, Hwang JH, Park EO, Lee SO, Chung YJ, Chung MJ, Lim S, Lim TJ, Ha Y, Park BH, Chae SW. Regulation of alcohol and acetaldehyde metabolism by a mixture of lactobacillus and bifidobacterium species in human. Nutrients. 2021; 13: 1875. doi: 10.3390/nu13061875.

80. Veenstra J, van de Pol H, Schaafsma G. Moderate alcohol consumption and platelet aggregation in healthy middle-aged men. Alcohol. 1990; 7: 547–9. doi: 10.1016/0741-8329(90)90046-f.

81. Rodda LN, Gerostamoulos D, Drummer OH. Detection of iso-a-acids to confirm beer consumption in postmortem specimens. Drug Test Anal. 2015; 7: 65–74. doi: 10.1002/dta.1749.

82. Steiner I, Brauers G, Temme O, Daldrup T. A sensitive method for the determination of hordenine in human serum by ESI+ UPLC-MS/MS for forensic toxicological applications. Anal Bioanal Chem. 2016; 408: 2285–92. doi: 10.1007/s00216-016-9324-3.

83. Quifer-Rada P, Martínez-Hueíamo M, Jaúregui O, Chiva-Blanch G, Estruch R, Lamuela-Ravento RM. Analytical Condition Setting a Crucial Step in the Quantification of Unstable Polyphenols in Acidic Conditions: Analyzing Prenylflavanoids in Biological Samples by Liquid Chromatography− Electrospray Ionization Triple Quadruple Mass Spectrometry. Anal Chem. 2013; 85: 5547–54. doi: 10.1021/ac4007733.

84. Sommer T, Göen T, Budnik N, Pischetsrieder M. Absorption, Biokinetics, and Metabolism of the Dopamine D2 Receptor Agonist Hordenine (N , N -Dimethyltyramine) after Beer Consumption in Humans. J Agric Food Chem. 2020; 68: 1998–2006. doi: 10.1021/acs.jafc.9b06029.

85. Rodda LN, Gerostamoulos D, Drummer OH. Pharmacokinetics of reduced iso-α-acids in volunteers following clear bottled beer consumption. Forensic Sci Int. 2015; 250: 37–43. doi: 10.1016/j.forsciint.2015.01.039.

86. Rodda LN, Gerostamoulos D, Drummer OH. Pharmacokinetics of iso-α-acids in volunteers following the consumption of beer. J Anal Toxicol. 2014; 38: 354–9. doi: 10.1093/jat/bku038.

87. Sobiech M, Giebułtowicz J, Luliński P. Theoretical and experimental proof for selective response of imprinted sorbent – analysis of hordenine in human urine. J Chromatogr A. 2020; 1613: 460677. doi: 10.1016/j.chroma.2019.460677.

88. Rodda LN, Gerostamoulos D, Drummer OH. The rapid identification and quantification of iso-α-acids and reduced iso-α-acids in blood using UHPLC-MS/MS: Validation of a novel marker for beer consumption. Anal Bioanal Chem. 2013; 405: 9755–67. doi: 10.1007/s00216-013-7413-0.

89. Quifer-Rada P, Martínez-Huélamo M, Chiva-Blanch G, Jáuregui O, Estruch R, Lamuela-Raventós RM. Urinary isoxanthohumol is a specific and accurate biomarker of beer consumption. J Nutr. 2014; 144: 484–8. doi: 10.3945/jn.113.185199.

90. Quifer-Rada P, Chiva-Blanch G, Jáuregui O, Estruch R, Lamuela-Raventós RM. A discovery-driven approach to elucidate urinary metabolome changes after a regular and moderate consumption of beer and nonalcoholic beer in subjects at high cardiovascular risk. Mol Nutr Food Res. 2017; 61: 1600980. doi: 10.1002/mnfr.201600980.

91. Daimiel L, Micó V, Díez-Ricote L, Ruiz-Valderrey P, Istas G, Rodríguez-Mateos A, Ordovás JM. Alcoholic and Non-Alcoholic Beer Modulate Plasma and Macrophage microRNAs Differently in a Pilot Intervention in Humans with Cardiovascular Risk. Nutrients. 2020; 13: 69. doi: 10.3390/nu13010069.

92. Gürdeniz G, Jensen MG, Meier S, Bech L, Lund E, Dragsted LO. Detecting Beer Intake by Unique Metabolite Patterns. J Proteome Res. 2016; 15: 4544–56. doi: 10.1021/acs.jproteome.6b00635.

93. Marks SC, Mullen W, Borges G, Crozier A. Absorption, Metabolism, and Excretion of Cider Dihydrochalcones in Healthy Humans and Subjects with an Ileostomy. J Agric Food Chem. 2009; 57: 2009–15.

94. DuPont MS, Bennett RN, Mellon FA, Williamson G. Polyphenols from alcoholic apple cider are absorbed, metabolized and excreted by humans. J Nutr. 2002; 132: 172–5. doi: 10.1093/jn/132.2.172.

95. Zamora-Ros R, Rothwell JA, Achaintre D, Ferrari P, Boutron-Ruault M-C, Mancini FR, Affret A, Kühn T, Katzke V, Boeing H, Küppel S, Trichopoulou A, Lagiou P, et al. Evaluation of urinary resveratrol as a biomarker of dietary resveratrol intake in the European Prospective Investigation into Cancer and Nutrition (EPIC) study. Br J Nutr. 2017; 117: 1596–602. doi: 10.1017/s0007114517001465.

96. Edmands WMB, Ferrari P, Rothwell JA, Rinaldi S, Slimani N, Barupal DK, Biessy C, Jenab M, Clavel-Chapelon F, Fagherazzi G, Boutron-Ruault MC, Katzke VA, Kühn T, et al. Polyphenol metabolome in human urine and its association with intake of polyphenol-rich foods across European countries. Am J Clin Nutr. 2015; 102: 905–13. doi: 10.3945/ajcn.114.101881.

97. Zamora-Ros R, Urpí-Sardà M, Lamuela-Raventós RM, Estruch R, Martínez-González MÁ, Bulló M, Arós F, Cherubini A, Andres-Lacueva C. Resveratrol metabolites in urine as a biomarker of wine intake in free-living subjects: The PREDIMED Study. Free Radic Biol Med. 2009; 46: 1562–6. doi: 10.1016/j.freeradbiomed.2008.12.023.

98. Zamora-Ros R, Urpi-Sarda M, Lamuela-Raventós RM, Martínez-González MÁ, Salas-Salvadó J, Arós F, Fitó M, Lapetra J, Estruch R, Andres-Lacueva C. High urinary levels of resveratrol metabolites are associated with a reduction in the prevalence of cardiovascular risk factors in high-risk patients. Pharmacol Res. 2012; 65: 615–20. doi: 10.1016/j.phrs.2012.03.009.

99. Zamora-Ros R, Achaintre D, Rothwell JA, Rinaldi S, Assi N, Ferrari P, Leitzmann M, Boutron-Ruault M-C, Fagherazzi G, Auffret A, Kühn T, Katzke V, Boeing H, et al. Urinary excretions of 34 dietary polyphenols and their associations with lifestyle factors in the EPIC cohort study. Sci Rep. 2016; 6: 1–9. doi: 10.1038/srep26905.

100. Noh H, Freisling H, Assi N, Zamora-Ros R, Achaintre D, Affret A, Mancini F, Boutron-Ruault M-C, Flögel A, Boeing H, Kühn T, Schübel R, Trichopoulou A, et al. Identification of Urinary Polyphenol Metabolite Patterns Associated with Polyphenol-Rich Food Intake in Adults from Four European Countries. Nutrients. 2017; 9: 796. doi: 10.3390/nu9080796.

101. Domínguez-López I, Parilli-Moser I, Arancibia-Riveros C, Tresserra-Rimbau A, Martínez-González MA, Ortega-Azorín C, Salas-Salvadó J, Castañer O, Lapetra J, Arós F, Fiol M, Serra-Majem L, Pintó X, et al. Urinary tartaric acid, a biomarker of wine intake, correlates with lower total and ldl cholesterol. Nutrients. 2021; 13. doi: 10.3390/nu13082883.

102. Regal P, Porto-Arias JJ, Lamas A, Paz L, Barreiro F, Cepeda A. LC-MS as a tool to overcome the limitations of self-reported dietary assessments in the determination of wine intake. Separations. 2017; 4: 1–7. doi: 10.3390/separations4020017.

103. Ortuño J, Covas MI, Farre M, Pujadas M, Fito M, Khymenets O, Andres-Lacueva C, Roset P, Joglar J, Lamuela-Raventós RM, Torre R de la. Matrix effects on the bioavailability of resveratrol in humans. Food Chem. 2010; 120: 1123–30. doi: 10.1016/j.foodchem.2009.11.032.

104. Spaak J, Merlocco AC, Soleas GJ, Tomlinson G, Morris BL, Picton P, Notarius CF, Chan CT, Floras JS. Dose-related effects of red wine and alcohol on hemodynamics, sympathetic nerve activity, and arterial diameter. Am J Physiol Heart Circ Physiol. 2008; 294: 605–12. doi: 10.1152/ajpheart.01162.2007.

105. Rotches-Ribalta M, Andres-Lacueva C, Estruch R, Escribano E, Urpi-Sarda M. Pharmacokinetics of resveratrol metabolic profile in healthy humans after moderate consumption of red wine and grape extract tablets. Pharmacol Res. 2012; 66: 375–82. doi: 10.1016/j.phrs.2012.08.001.

106. Regueiro J, Vallverdú-Queralt A, Simal-Gándara J, Estruch R, Lamuela-Raventós R. Development of a LC-ESI-MS/MS approach for the rapid quantification of main wine organic acids in human urine. J Agric Food Chem. 2013; 61: 6763–8. doi: 10.1021/jf401839g.

107. Urpi-Sarda M, Zamora-Ros R, Lamuela-Raventos R, Cherubini A, Jauregui O, De La Torre R, Covas MI, Estruch R, Jaeger W, Andres-Lacueva C. HPLC-tandem mass spectrometric method to characterize resveratrol metabolism in humans. Clin Chem. 2007; 53: 292–9. doi: 10.1373/clinchem.2006.071936.

108. Lord RS, Burdette CK, Bralley A. Urinary Markers of Yeast Overgrowth. Integr Med. 2004; 3: 24–9.

109. Boronat A, Martínez-Huélamo M, Cobos A, de la Torre R. Wine and Olive Oil Phenolic Compounds Interaction in Humans. Diseases. 2018; 6: 76. doi: 10.3390/diseases6030076.

110. Motilva MJ, Macià A, Romero MP, Rubió L, Mercader M, González-Ferrero C. Human bioavailability and metabolism of phenolic compounds from red wine enriched with free or nano-encapsulated phenolic extract. J Funct Foods. 2016; 25: 80–93. doi: 10.1016/j.jff.2016.05.013.

111. Regueiro J, Vallverdú-Queralt A, Simal-Gándara J, Estruch R, Lamuela-Raventós RM. Urinary tartaric acid as a potential biomarker for the dietary assessment of moderate wine consumption: A randomised controlled trial. Br J Nutr. 2014; 111: 1680–5. doi: 10.1017/s0007114513004108.

112. Vitaglione P, Sforza S, Galaverna G, Ghidini C, Caporaso N, Vescovi PP, Fogliano V, Marchelli R. Bioavailability of trans-resveratrol from red wine in humans. Mol Nutr Food Res. 2005; 49: 495–504. doi: 10.1002/mnfr.200500002.

113. Vázquez-Fresno R, Llorach R, Urpi-Sarda M, Khymenets O, Bulló M, Corella D, Fitó M, Martínez-González MA, Estruch R, Andres-Lacueva C. An NMR metabolomics approach reveals a combined-biomarkers model in a wine interventional trial with validation in free-living individuals of the PREDIMED study. Metabolomics. 2015; 11: 797–806. doi: 10.1007/s11306-014-0735-x.

114. Chiva-Blanch G, Urpi-Sarda M, Ros E, Arranz S, Valderas-Martínez P, Casas R, Sacanella E, Llorach R, Lamuela-Raventos RM, Andres-Lacueva C, Estruch R. Dealcoholized red wine decreases systolic and diastolic blood pressure and increases plasma nitric oxide: Short communication. Circ Res. 2012; 111: 1065–8. doi: 10.1161/circresaha.112.275636.

115. Chiva-Blanch G, Urpi-Sarda M, Ros E, Valderas-Martinez P, Casas R, Arranz S, Guillén M, Lamuela-Raventós RM, Llorach R, Andres-Lacueva C, Estruch R. Effects of red wine polyphenols and alcohol on glucose metabolism and the lipid profile: A randomized clinical trial. Clin Nutr. 2013; 32: 200–6. doi: 10.1016/j.clnu.2012.08.022.

116. Chiva-Blanch G, Urpi-Sarda M, Llorach R, Rotches-Ribalta M, Guilleń M, Casas R, Arranz S, Valderas-Martinez P, Portoles O, Corella D, Tinahones F, Lamuela-Raventos RM, Andres-Lacueva C, et al. Differential effects of polyphenols and alcohol of red wine on the expression of adhesion molecules and inflammatory cytokines related to atherosclerosis: A randomized clinical trial. Am J Clin Nutr. 2012; 95: 326–34. doi: 10.3945/ajcn.111.022889.

117. Vázquez-Fresno R, Llorach R, Alcaro F, Rodríguez MÁ, Vinaixa M, Chiva-Blanch G, Estruch R, Correig X, Andrés-Lacueva C. 1H-NMR-based metabolomic analysis of the effect of moderate wine consumption on subjects with cardiovascular risk factors. Electrophoresis. 2012; 33: 2345–54. doi: 10.1002/elps.201100646.

118. Zamora-Ros R, Urpí-Sardà M, Lamuela-Raventós RM, Estruch R, Vázquez-Agell M, Serrano-Martínez M, Jaeger W, Andres-Lacueva C. Diagnostic performance of urinary resveratrol metabolites as a biomarker of moderate wine consumption. Clin Chem. 2006; 52: 1373–80. doi: 10.1373/clinchem.2005.065870.

119. Urpi-Sarda M, Boto-Ordóñez M, Queipo-Ortuño MI, Tulipani S, Corella D, Estruch R, Tinahones FJ, Andres-Lacueva C. Phenolic and microbial-targeted metabolomics to discovering and evaluating wine intake biomarkers in human urine and plasma. Electrophoresis. 2015; 36: 2259–68. doi: 10.1002/elps.201400506.

120. Sacanella E, Vázquez-Agell M, Mena MP, Antúnez E, Fernández-Solá J, Nicolás JM, Lamuela-Raventós RM, Ros E, Estruch R. Down-regulation of adhesion molecules and other inflammatory biomarkers after moderate wine consumption in healthy women: A randomized trial. Am J Clin Nutr. 2007; 86: 1463–9. doi: 10.1093/ajcn/86.5.1463.

121. Rotches-Ribalta M, Urpi-Sarda M, Llorach R, Boto-Ordoñez M, Jauregui O, Chiva-Blanch G, Perez-Garcia L, Jaeger W, Guillen M, Corella D, Tinahones FJ, Estruch R, Andres-Lacueva C. Gut and microbial resveratrol metabolite profiling after moderate long-term consumption of red wine versus dealcoholized red wine in humans by an optimized ultra-high-pressure liquid chromatography tandem mass spectrometry method. J Chromatogr A. 2012; 1265: 105–13. doi: 10.1016/j.chroma.2012.09.093.

122. Esteban-Fernández A, Ibañez C, Simó C, Bartolomé B, Moreno-Arribas MV. An Ultrahigh-Performance Liquid Chromatography-Time-of-Flight Mass Spectrometry Metabolomic Approach to Studying the Impact of Moderate Red-Wine Consumption on Urinary Metabolome. J Proteome Res. 2018; 17: 1624–35. doi: 10.1021/acs.jproteome.7b00904.

123. González-Domínguez R, Jáuregui O, Mena P, Hanhineva K, Tinahones FJ, Angelino D, Andrés-Lacueva C. Quantifying the human diet in the crosstalk between nutrition and health by multi-targeted metabolomics of food and microbiota-derived metabolites. Int J Obes. 2020; 44: 2372–81. doi: 10.1038/s41366-020-0628-1.

124. Roth I, Casas R, Ribó-Coll M, Estruch R. Consumption of aged white wine under a veil of flor reduces blood pressure-increasing plasma nitric oxide in men at high cardiovascular risk. Nutrients. 2019; 11: 1266. doi: 10.3390/nu11061266.

125. Roth I, Casas R, Medina-Remón A, Lamuela-Raventós RM, Estruch R. Consumption of aged white wine modulates cardiovascular risk factors via circulating endothelial progenitor cells and inflammatory biomarkers. Clin Nutr. 2019; 38: 1036–44. doi: 10.1016/j.clnu.2018.06.001.

126. Queipo-Ortuño MI, Boto-Ordóñez M, Murri M, Gomez-Zumaquero JM, Clemente-Postigo M, Estruch R, Cardona Diaz F, Andrés-Lacueva C, Tinahones FJ. Influence of red wine polyphenols and ethanol on the gut microbiota ecology and biochemical biomarkers. Am J Clin Nutr. 2012; 95: 1323–34. doi: 10.3945/ajcn.111.027847.

127. Clemente-Postigo M, Queipo-Ortuno MI, Boto-Ordonez M, Coin-Araguez L, del Mar Roca-Rodriguez M, Delgado-Lista J, Cardona F, Andres-Lacueva C, Tinahones FJ. Effect of acute and chronic red wine consumption on lipopolysaccharide concentrations1-3. Am J Clin Nutr. 201; 97: 1053–61. doi: 10.3945/ajcn.112.051128.

128. Pignatelli P, Ghiselli A, Buchetti B, Carnevale R, Natella F, Germanò G, Fimognari F, Di Santo S, Lenti L, Violi F. Polyphenols synergistically inhibit oxidative stress in subjects given red and white wine. Atherosclerosis. 2006; 188: 77–83. doi: 10.1016/j.atherosclerosis.2005.10.025.

129. Gresele P, Pignatelli P, Guglielmini G, Carnevale R, Mezzasoma AM, Ghiselli A, Momi S, Violi F. Resveratrol, at concentrations attainable with moderate wine consumption, stimulates human platelet nitric oxide production. J Nutr. 2008; 138: 1602–8. doi: 10.1093/jn/138.9.1602.

130. Schulz K, Schlenz K, Metasch R, Malt S, Römhild W, Dreßler J. Determination of anethole in serum samples by headspace solid-phase microextraction-gas chromatography–mass spectrometry for congener analysis. J Chromatogr A. 2008; 1200: 235–41. doi: 10.1016/j.chroma.2008.05.066.

131. Schulz K, Bertau M, Schlenz K, Malt S, Dreßler J, Lachenmeier DW. Headspace solid-phase microextraction–gas chromatography–mass spectrometry determination of the characteristic flavourings menthone, isomenthone, neomenthol and menthol in serum samples with and without enzymatic cleavage to validate post-offence alcohol . Anal Chim Acta. 2009; 646: 128–40. doi: 10.1016/j.aca.2009.05.010.

132. Oliphant K, Allen-Vercoe E. Macronutrient metabolism by the human gut microbiome: Major fermentation by-products and their impact on host health. Microbiome. 2019; 7. doi: 10.1186/s40168-019-0704-8.

133. Kok EE, Wielders JPM, Jong PCMP, Defourny H, Ronde SJA, Wiel A van de. Biomarkers of excessive alcohol intake in alcohol addicts. Ned Tijdschr Klin Chem Labgeneesk. 2014; 39: 185–8.

134. Peterson K. Biomarkers for alcohol use and abuse: A summary. Alcohol Res Health. 2004; 28: 30–7.

135. Bean P, Harasymiw J, Peterson CM, Javors M. Innovative technologies for the diagnosis of alcohol abuse and monitoring abstinence. Alcohol Clin Expe Res. 2001; 25: 309–16. doi: 10.1111/j.1530-0277.2001.tb02214.x.

136. Wilkens TL, Tranæs K, Eriksen JN, Dragsted LO. Moderate alcohol consumption and lipoprotein subfractions: a systematic review of intervention and observational studies. Nutr Rev. 2022; 80: 1311–39. doi: 10.1093/nutrit/nuab102.

137. Quifer-Rada P, Vallverdú-Queralt A, Martínez-Huélamo M, Chiva-Blanch G, Jáuregui O, Estruch R, Lamuela-Raventós R. A comprehensive characterisation of beer polyphenols by high resolution mass spectrometry (LC–ESI-LTQ-Orbitrap-MS). Food Chem. 2015; 169: 336–43. doi: 10.1016/j.foodchem.2014.07.154.

138. Mazzilli KM, McClain KM, Lipworth L, Playdon MC, Sampson JN, Clish CB, Gerszten RE, Freedman ND, Moore SC. Identification of 102 Correlations between Serum Metabolites and Habitual Diet in a Metabolomics Study of the Prostate, Lung, Colorectal, and Ovarian Cancer Trial. J Nutr. 2020; 150: 694–703. doi: 10.1093/jn/nxz300.

139. Soldevila-Domenech N, Boronat A, Mateus J, Diaz-Pellicer P, Matilla I, Pérez-Otero M, Aldea-Perona A, de la Torre R. Generation of the Antioxidant Hydroxytyrosol from Tyrosol Present in Beer and Red Wine in a Randomized Clinical Trial. Nutrients. 2019; 11: 2241. doi: 10.3390/nu11092241.

140. Lang R, Lang T, Bader M, Beusch A, Schlagbauer V, Hofmann T. High-Throughput Quantitation of Proline Betaine in Foods and Suitability as a Valid Biomarker for Citrus Consumption. J Agric Food Chem. 2017; 65: 1613–9. doi: 10.1021/acs.jafc.6b05824.

141. Husøy T, Haugen M, Murkovic M, Jöbstl D, Stølen LH, Bjellaas T, Rønningborg C, Glatt H, Alexander J. Dietary exposure to 5-hydroxymethylfurfural from Norwegian food and correlations with urine metabolites of short-term exposure. Food Chem Toxicol. 2008; 46: 3697–702. doi: 10.1016/j.fct.2008.09.048.

142. Lindenthal B, Von Bergmann K. Urinary excretion and serum concentration of mevalonic acid during acute intake of alcohol. Metabolism. 2000; 49: 62–6. doi: 10.1016/s0026-0495(00)90713-3.

143. Tsuchiya H, Yamada K, Tajima K, Hayashi T. Urinary excretion of tetrahydro-β-carbolines relating to ingestion of alcoholic beverages. Alcohol Alcohol. 1996; 31: 197–203. doi: 10.1093/oxfordjournals.alcalc.a008132.

144. De La Torre R, Covas MI, Pujadas MA, Fitó M, Farré M. Is dopamine behind the health benefits of red wine? Eur J Nutr. 2006; 45: 307–10. doi: 10.1007/s00394-006-0596-9.

145. Boronat A, Mateus J, Soldevila-Domenech N, Guerra M, Rodríguez-Morató J, Varon C, Muñoz D, Barbosa F, Morales JC, Gaedigk A, Langohr K, Covas M-I, Pérez-Mañá C, et al. Cardiovascular benefits of tyrosol and its endogenous conversion into hydroxytyrosol in humans. A randomized, controlled trial. Free Radic Biol Med. 2019; 143: 471–81. doi: 10.1016/j.freeradbiomed.2019.08.032.

146. Rosa MB, Fernandes M dos S, Bonjardim LR, Gavião MBD, Calixto LA, Castelo PM. Evaluation of oral mechanical and gustatory sensitivities and salivary cotinine levels in adult smokers. Acta Odontol Scand. 2020; 78: 256–64. doi: 10.1080/00016357.2019.1694978.

147. Bitsch R, Netzel M, Frank T, Strass G, Bitsch I. Bioavailability and Biokinetics of Anthocyanins From Red Grape Juice and Red Wine. J Biomed Biotechnol. 2004; 2004: 293–8. doi: 10.1155/s1110724304403106.

148. Frank T, Netzel M, Strass G, Bitsch R, Bitsch I. Bioavailability of anthocyanidin-3-glucosides following consumption of red wine and red grape juice. Can J Physiol Pharmacol. 2003; 81: 423–35. doi: 10.1139/y03-038.

149. Lapidot T, Harel S, Granit R, Kanner J. Bioavailability of Red Wine Anthocyanins as Detected in Human Urine. J Agric Food Chem. 1998; 46: 4297–302. doi: 10.1021/jf980007o.

150. Boto-Ordóñez M, Urpi-Sarda M, Queipo-Ortuño MI, Corella D, Tinahones FJ, Estruch R, Andres-Lacueva C. Microbial metabolomic fingerprinting in urine after regular dealcoholized red wine consumption in humans. J Agric Food Chem. 2013; 61: 9166–75. doi: 10.1021/jf402394c.

151. Cartron E, Fouret G, Carbonneau M-A, Lauret C, Michel F, Monnier L, Descomps B, Léger CL. Red-wine Beneficial Long-term Effect on Lipids but not on Antioxidant Characteristics in Plasma in a Study Comparing Three Types of Wine—Description of two O-methylated Derivatives of Gallic Acid in Humans. Free Radic Res. 2003; 37: 1021–35. doi: 10.1080/10715760310001598097.

152. Tsang C, Higgins S, Duthie GG, Duthie SJ, Howie M, Mullen W, Lean MEJ, Crozier A, Albert CM, Manson JE, Cook NR, Ajani UA, Gaziano JM, et al. The influence of moderate red wine consumption on antioxidant status and indices of oxidative stress associated with CHD in healthy volunteers. Br J Nutr. 2005; 93: 233. doi: 10.1079/bjn20041311.

153. Donovan JL, Kasim-Karakas S, German JB, Waterhouse AL, Ameer B, Weintraub RA, Johnson J V., Yost RA, Rouseff RL, Arts ICW, Hollman PCH, Feskens EJM, Bueno de Mesquita HB, et al. Urinary excretion of catechin metabolites by human subjects after red wine consumption. Br J Nutr. 2002; 87: 31. doi: 10.1079/bjn2001482.

154. Bell JR, Donovan JL, Wong R, Waterhouse AL, German JB, Walzem RL, Kasim-Karakas SE. (+)-Catechin in human plasma after ingestion of a single serving of reconstituted red wine. Am J Clin Nutr. 2000; 71: 103–8. doi: 10.1093/ajcn/71.1.103.

155. Donovan JL, Bell JR, Kasim-Karakas S, German JB, Walzern RL, Hansen RJ, Waterhouse AL. Catechin is present as metabolites in human plasma after consumption of red wine. J Nutr. 1999; 129: 1662–8. doi: 10.1093/jn/129.9.1662.

156. Badía E, Sacanella E, Fernández-Solá J, Nicolás JM, Antúnez E, Rotilio D, de Gaetano G, Urbano-Márquez A, Estruch R. Decreased tumor necrosis factor-induced adhesion of human monocytes to endothelial cells after moderate alcohol consumption. Am J Clin Nutr. 2004; 80: 225–30. doi: 10.1093/ajcn/80.1.225.

157. Estruch R, Sacanella E, Badia E, Antúnez E, Nicolás JM, Fernández-Solá J, Rotilio D, de Gaetano G, Rubin E, Urbano-Márquez A. Different effects of red wine and gin consumption on inflammatory biomarkers of atherosclerosis: a prospective randomized crossover trial: Effects of wine on inflammatory markers. Atherosclerosis. 2004; 175: 117–23. doi: 10.1016/j.atherosclerosis.2004.03.006.

158. Estruch R, Sacanella E, Mota F, Chiva-Blanch G, Antúneza E, Casals E, Deulofeu R, Rotilio D, Andres-Lacueva C, Lamuela-Raventos RM, de Gaetano G, Urbano-Marquez A. Moderate consumption of red wine, but not gin, decreases erythrocyte superoxide dismutase activity: A randomised cross-over trial. Nutr Metab Cardiovasc Dis. 2011; 21: 46–53. doi: 10.1016/j.numecd.2009.07.006.

159. de Vries JH, Hollman PC, van Amersfoort I, Olthof MR, Katan MB. Red wine is a poor source of bioavailable flavonols in men. J Nutr. 2001; 131: 745–8.

160. Vázquez-Fresno R, Llorach R, Perera A, Mandal R, Feliz M, Tinahones FJ, Wishart DS, Andres-Lacueva C. Clinical phenotype clustering in cardiovascular risk patients for the identification of responsive metabotypes after red wine polyphenol intake. J Nutr Biochem. 2016; 28: 114–20. doi: 10.1016/j.jnutbio.2015.10.002.

161. Gutiérrez-Díaz I, Fernández-Navarro T, Salazar N, Bartolomé B, Moreno-Arribas MV, De Andres-Galiana EJ, Fernández-Martínez JL, De Los Reyes-Gavilán CG, Gueimonde M, González S. Adherence to a mediterranean diet influences the fecal metabolic profile of microbial-derived phenolics in a Spanish cohort of middle-age and older people. J Agric Food Chem. 2017; 65: 586–95. doi: 10.1021/acs.jafc.6b04408.

162. Muñoz-González I, Jiménez-Girón A, Martín-Álvarez PJ, Bartolomé B, Moreno-Arribas MV. Profiling of Microbial-Derived Phenolic Metabolites in Human Feces after Moderate Red Wine Intake. J Agric Food Chem. 2013; 61: 9470–9. doi: 10.1021/jf4025135.

163. Jiménez-Girón A, Queipo-Ortuño MI, Boto-Ordóñez M, Muñoz-González I, Sánchez-Patán F, Monagas M, Martín-Álvarez PJ, Murri M, Tinahones FJ, Andrés-Lacueva C, Bartolomé B, Moreno-Arribas MV. Comparative Study of Microbial-Derived Phenolic Metabolites in Human Feces after Intake of Gin, Red Wine, and Dealcoholized Red Wine. J Agric Food Chem. 2013; 61: 3909–15. doi: 10.1021/jf400678d.

164. Mennen LI, Sapinho D, Ito H, Bertrais S, Galan P, Hercberg S, Scalbert A. Urinary flavonoids and phenolic acids as biomarkers of intake for polyphenol-rich foods. Br J Nutr. 2006; 96: 191. doi: 10.1079/bjn20061808.

165. Caccetta RA, Croft KD, Beilin LJ, Puddey IB. Ingestion of red wine significantly increases plasma phenolic acid concentrations but does not acutely affect ex vivo lipoprotein oxidizability. Am J Clin Nutr. 2000; 71: 67–74.

166. Abu-Amsha Caccetta R, Burke V, Mori TA, Beilin LJ, Puddey IB, Croft KD. Red wine polyphenols, in the absence of alcohol, reduce lipid peroxidative stress in smoking subjects. Free Radic Biol Med. 2001; 30: 636–42. doi: 10.1016/s0891-5849(00)00497-4.

167. Barden A, Shinde S, Phillips M, Beilin L, Mas E, Hodgson JM, Puddey I, Mori TA. The effects of alcohol on plasma lipid mediators of inflammation resolution in patients with Type 2 diabetes mellitus. Prostaglandins Leukot Essent Fatty Acids. 2018; 133: 29–34. doi: 10.1016/j.plefa.2018.04.004.

168. Nardini M, Forte M, Vrhovsek U, Mattivi F, Viola R, Scaccini C. White Wine Phenolics Are Absorbed and Extensively Metabolized in Humans. J Agric Food Chem. 2009; 57: 2711–8. doi: 10.1021/jf8034463.

169. Simonetti P, Gardana C, Pietta P. Caffeic acid as biomarker of red wine intake. Meth Enzymol. 2001; 335: 122–30. doi: 10.1016/s0076-6879(01)35237-0.

170. Simonetti P, Gardana C, Pietta P. Plasma Levels of Caffeic Acid and Antioxidant Status after Red Wine Intake. J Agric Food Chem. 2001; 49: 5964-68. doi: 10.1021/jf010546k.

171. Regueiro J, Vallverdú-Queralt A, Simal-Gándara J, Estruch R, Lamuela-Raventós RM. Urinary tartaric acid as a potential biomarker for the dietary assessment of moderate wine consumption: A randomised controlled trial. Br J Nutr. 2014; 111: 1680–5. doi: 10.1017/s0007114513004108.

172. Jian L. Alcohol and urinary 2-thiothiazolidine-4-carboxylic acid. Toxicol Lett. 2002; 134: 277–83. doi: 10.1016/s0378-4274(02)00177-7.
